# Development of quality outcome indicators to improve the quality of urinary and faecal continence care

**DOI:** 10.1007/s00192-018-3768-2

**Published:** 2018-10-16

**Authors:** Adrian Wagg, Dianne Gove, Kai Leichsenring, Joan Ostaszkiewicz

**Affiliations:** 1 Department of Medicine, University of Alberta, Edmonton, AB Canada; 2 Division of Geriatric Medicine, University of Alberta, 1-198, Clinical Sciences Building, 11350 - 83 Avenue, Edmonton, Alberta T6G 2P4 Canada; 3 Alzheimer Europe, Luxembourg City, Luxembourg; 4 European Centre for Social Welfare Policy and Research Vienna, Vienna, Austria; 5 Centre for Quality and Patient Safety Research, Deakin University, Geelong, Australia

**Keywords:** Urinary incontinence, Fecal incontinence, Outcome measures, Performance indicators, Toileting and containment

## Abstract

**Introduction and hypothesis:**

Despite the range of treatment options available, relatively few people with incontinence find a total cure. The importance of daily management with toileting and containment cannot be underestimated. To our knowledge, there are no outcome measures to benchmark good care. The aim of this study was to create a set of key performance indicators (KPIs) to measure outcomes for toileting and containment.

**Methods:**

An expert panel (EP) defined a set of KPIs using evidence from a scoping review, stakeholder engagement, and expert consensus. Peer reviewed articles, high-quality grey literature and international and national standards were reviewed to identify existing measures for management. These findings were augmented by an exercise involving patients, caregivers, nurses, clinicians, payers, policy makers and care providers to prioritise the findings and identify additional areas of interest.

**Results:**

The final set of 14 KPIs includes quality indicators of process and outcome for those managed with a toileting and containment strategy and is relevant for both care-independent and -dependent persons. Rates of assessment, days waiting for specialist assessment, rates of return to work and those rating their quality of life as good or acceptable are captured. An indicator of well-being for caregivers and the economic costs of poor care are also defined.

**Conclusions:**

The set of KPIs to measure outcomes from toileting and containment strategies describes the components of each to encourage integration into existing quality frameworks. Each KPI has been refined and detailed to encourage this. If implemented, resulting benchmarking data will facilitate care quality improvement and inform value-based care procurement and provision of toileting and containment strategies.

**Electronic supplementary material:**

The online version of this article (10.1007/s00192-018-3768-2) contains supplementary material, which is available to authorized users

## Introduction

Urinary incontinence (UI) is a common, distressing condition for many, particularly in later life. Current estimates suggest that UI affects the lives of an estimated 400 million people worldwide [1], and is more common in women than men [1, 2]. The prevalence of fecal incontinence (FI) among community dwelling adults in the USA was recently estimated at 8.39%, with FI more common in women with UI [3]. The prevalence of both UI [1] and FI [3] increases with age.

Urinary incontinence has a profound impact on a person’s quality of life and social functioning in addition to being associated with adverse health outcomes such as depression, falls and urinary tract infection [4].

There are many examples of national and international guidelines that offer recommendations for the care of people with both urinary and fecal incontinence [5, 6]. Most guidelines note that conservative therapies that promote self-management with toileting strategies may result in an acceptable outcome from care. There have been few attempts to systematically measure the quality of care provision for incontinence. A national clinical effectiveness project in England and Wales reported the variability of care and that quality care provision was in the hands of committed individuals, rather than a programmatic provision of services [7]. Aside from product guidelines on absorbent aids [8], there is a marked lack of auditable quality standards for toileting strategies and containment products [9]. With increased attention to value-based healthcare, a suite of outcome measures for overactive bladder has been developed for incorporation into administrative data sets [10]. This project was undertaken to complement continence-related key performance indicators (KPIs) by identifying and defining a set of indicators applicable to people with bladder and bowel problems who manage their care with a combination of toileting and containment with the intent of attaining contained, social continence [11].

## Materials and methods

**Fig. 1:**
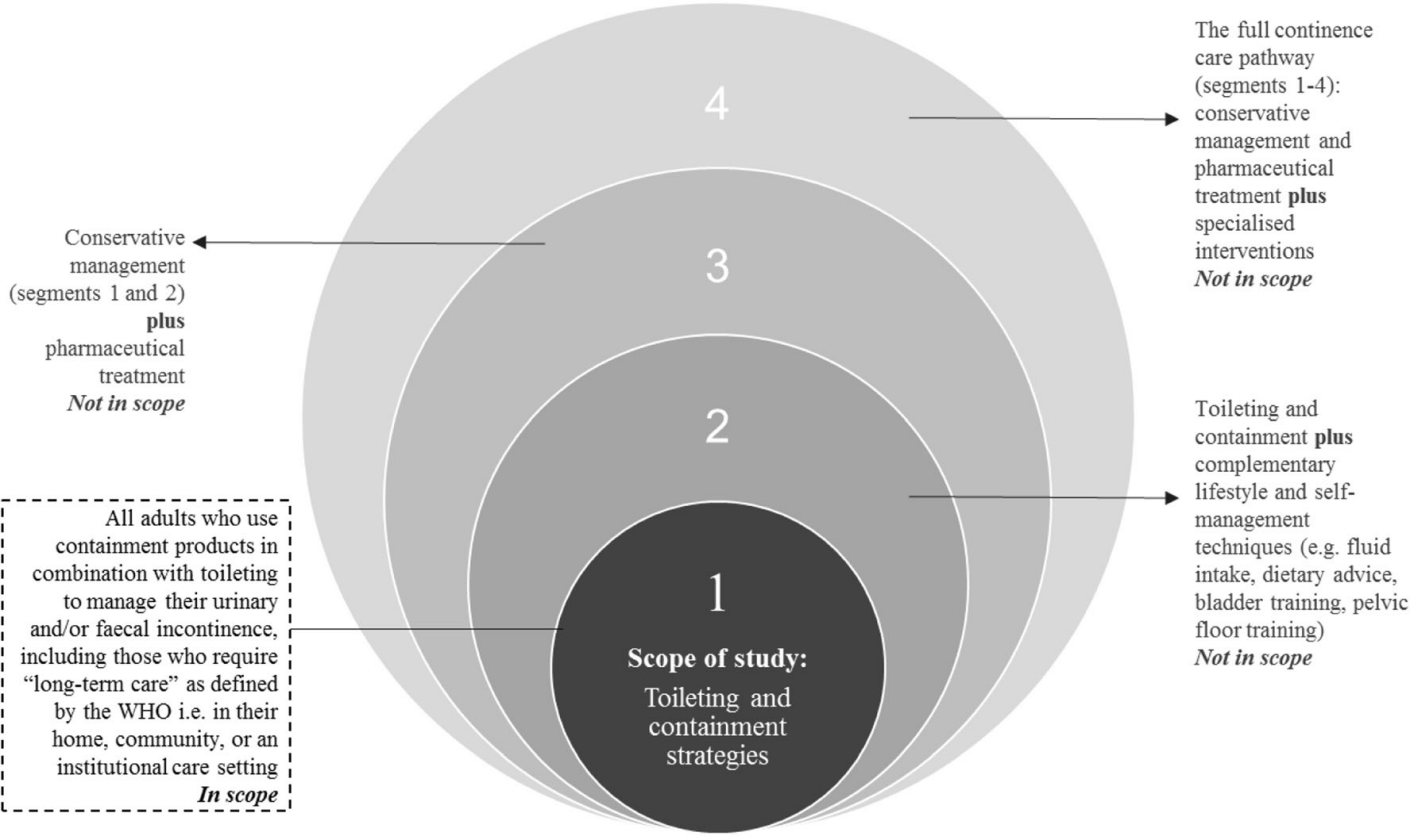
Scope of study

A multi-disciplinary international expert panel (EP) was convened to define a set of outcome, process and structure KPIs to measure toileting and containment strategies for all adults to manage their UI and/or FI (Fig. 1). The EP consisted of a geriatrician, a nurse, a payer, a social scientist, and a patient and caregiving representative from the European and North American regions. Using a consensus-driven method, the panel aimed to define a suite of KPIs that took into account the different needs of people with incontinence and different stakeholders in care delivery by following the applicability matrix shown in Table 1.

**Table 1:** Applicability matrix of the key performance indicators (KPIs)

Target population	KPI type	Care setting	Domain
Care-independent persons with incontinence	Outcome	Institutional^a^	Clinical
Care-dependent persons with incontinence who can express themselves	Process	Community^a^	Quality of life
Care-dependent persons with incontinence who cannot express themselves	Structure	Home^a^	Economic

^a^As defined by the WHO (WHO Secretariat, 2016)

The project comprised three phases: evidence-gathering, evidence synthesis, and KPI generation and validation. The panel felt that there was no requirement to differentiate KPI based upon the nature of the incontinence, given the relative frequency of combined bladder and bowel dysfunction in the populations likely to be managed by toileting and containment.

### Ethical approval

The study was approved by the Health Research Ethics Board of the University of Alberta (Pro00073594). All participants in any stage of the study gave informed consent to take part.

### Study applicability

To ensure wide applicability, the EP considered different types of persons with incontinence, care settings and intended users throughout the study.

The KPIs were designed to be applicable and relevant to three types of persons with incontinence:

1. Care-independent, defined as independent users of toileting and containment products
2. Care-dependent able to indicate the need to toilet, defined as care-dependent users who are able to indicate the need to toilet and manage their continence with containment products
3. Care-dependent unable to indicate the need to toilet, defined as care-dependent users of containment products who are unable to indicate the need to toilet and have their continence managed with containment products (information or choice may be given to or data may be gathered via a proxy such as a caregiving relative or professional caregiver)

The EP considered care-dependent persons as those who needed assistance of another person to achieve successful, social continence and additionally defined those people who could not indicate the need to toilet as those who had cognitive impairment to a degree that significantly interfered with the capacity to make decisions or indicate their need for care/made the person dependent upon the opinions/support of others when care needs were assessed.

In addition, three care settings as defined by the World Health Organisation [12] were considered to be applicable to the study:

1. Institutional, services provided in residential long-term care settings
2. Community services, those provided in the community, e.g. GP practice and other primary care continence centres
3. Services provided by a healthcare professional in a person’s home

The KPIs were developed to be applicable to various users, covering 12 stakeholder types across the spectrum of continence care provision: general practitioners, specialist physicians, physiotherapists, nurses with continence care skills and training, care team leaders, continence service providers, professional carers, caregiving relatives, organisations that represent persons with incontinence, organisations that represent caregiving relatives, payers and policy makers.

### Evidence gathering

This study used a scoping review method, which is typically used to gather evidence where there is a paucity of data, to identify all available evidence, regardless of quality, to synthesise the available knowledge and identify gaps in that knowledge [13]. In addition to academic articles, grey literature (materials and research produced by organisations outside traditional academic publishing and distribution channels) was also searched to capture guidelines and relevant policy documents. The time allotted for grey literature search was 1 h or until saturation was reached, whichever came first. Saturation was defined as not identifying any new literature to include in the analysis for 30 min or 5 consecutive search pages, whichever came first. The predefined time limit/saturation was set as a pragmatic limit, while allowing a comprehensive search to be performed. Data were supplemented by qualitative and quantitative findings through a stakeholder engagement exercise to provide an insight into potential gaps in the literature.

#### Scoping review

**Fig. 2:**
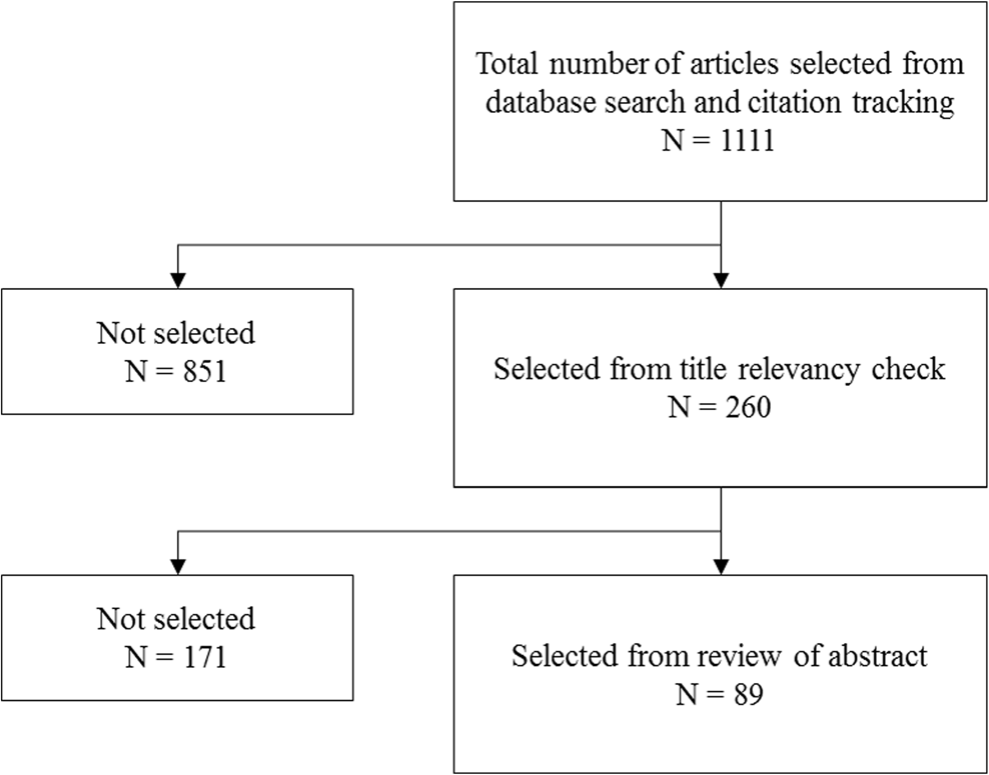
Studies identified in the scoping review

A search for articles relating to KPIs to measure daily continence management (PubMed, The Cochrane Library, Centre for Review and Dissemination), and search engines using a combination of search terms prioritised by the EP (Table 2, Fig. 2) was conducted. In line with the scoping method, evidence from randomised trials, quasi-experimental studies and other reports describing any KPIs utilised to measure outcomes in urinary and/or faecal daily continence management was gathered. Further articles were retrieved through citation-tracking of original articles, in addition to investigation of grey literature via the application of EP prioritised search terms in search engines. Non-English language references were excluded unless there was sufficient explanatory text in English. Articles had to be relevant to daily continence management with toileting and containment strategies provided for adults, including care independent and care dependent persons. Articles relating to conservative management of incontinence, pharmaceutical treatments, surgical intervention, treatment and condition management, and children were excluded (Fig. 1).

**Table 2:** Prioritised search terms

High priority search term / group of search terms selected by the expert panel	
Containment / Containment management / Management for containment / containment strategy	
Outcome measure(s) / Measuring quality	
Self-management / independence	
Quality of life	
Key Performance Indicator / KPI	
Indicator / Quality indicator	
Incontinence associated dermatitis / Skin health / Skin damage / Pressure ulcers	
Patient / user / consumer	
Incontinence services / Incontinence / Continence / Continence services	
Patient reported outcomes / PRO(s) / PROM(s)	
Cost / Costs / Total cost	
Value / economic evaluation/analysis/assessment/study/studies / health technology assessment / HTA	
Effectiveness / Efficacy	
Toileting skills	
Care / Carer / Informal carer / Family carer / Care giving relative	
Elderly	
Cognitively impaired / cognitive damage / neurologically impaired / neurological damage / mentally impaired / mental damage / mobility impaired / visually impaired / eyesight damage / hand dexterity impaired

#### Prioritisation criteria

Each expert panellist prioritised from among the long list of potential KPIs using four criteria:

1. Scope: KPI is within the scope of research (Fig. 1)
2. Relevant: KPI is relevant to:
  - Patients
  - Care providers
  - Payers
3. Measurable: KPI is linked to a process that can be measured
4. Robust: KPI is supported by validated endpoints

Each KPI was ranked high, medium or low priority based on prioritisation criteria agreed a priori (Supplementary Fig. A, Supplementary Table A) and these were then collectively reviewed and discussed by the EP to reach a consensus prioritisation through a Delphi process. KPI titles identified as high priority were further reviewed and revised by the EP to form a short list of KPI titles to test in the stakeholder engagement exercise.

#### Stakeholder engagement

A total of 58 participants with knowledge of continence management across North America and Europe engaged in a 60-min online virtual exercise moderated by telephone. Participants included: physicians, nurses, formal caregivers, caregiving relatives, care managers, physiotherapists, containment product users and user representatives, payers, health economists, policy makers and academics. Participants, a convenience sample, were identified through the literature search and from the recommendations of the EP. The objective was to sense check the expert panel-reviewed short list of KPI titles and to identify further areas of interest. The participants were asked a mix of 17 quantitative and qualitative questions and were asked to rate each potential KPI on a scale of 1 to 10, where 1 was not valuable at all and 10 was invaluable. Findings were reviewed and discussed with the EP in a workshop (Supplementary Table B).

### Evidence synthesis

Synthesis of evidence and drafting of the KPI followed an iterative process where the EP reviewed and discussed the findings throughout the study. For each step, the EP reached a consensus on structure and content. Where there was disagreement regarding the inclusion/exclusion of a title, a majority opinion was required for resolution. The five members of the panel and the research team met once face-to-face for the EP workshop and nine times by teleconference to synthesise the evidence and validate the findings.

#### Expert panel workshop

Following the stakeholder engagement exercise, a face-to-face workshop was held with a focus on: key themes, KPI title ratings per domain category, and additional suggested titles. Each title was discussed alongside the Prioritisation Criteria ranking and stakeholder engagement rating. Suggested additional KPIs were also considered. The consensus method was used to determine whether each was included, rephrased or removed. The workshop led to the development of a refined short list of KPIs across clinical, quality of life and economic domains based on the evidence gathered via the scoping review and stakeholder engagement exercise (Supplementary Table C).

#### Selection of core KPI

To identify and develop in detail a set of core KPIs, the EP, via the Delphi panel, selected a set of titles using the applicability matrix (Table 1). The EP developed a set of criteria to select which KPI titles should be taken forward as core KPIs and developed in detail. These included the possibility of measurement, operationalisation, that the time and cost of measurement should be justifiable, and that the set of KPIs should include both objective and subjective measures.

### KPI generation and validation

Each of the KPIs was developed in detail and validated by the EP (Table 3). The EP collectively reviewed, refined and validated each detailed core KPI (Table 4). The consensus method was used before applying EP recommendations. Where there was a disagreement regarding the detail in a core KPI, a majority opinion was required for resolution. For each core KPI, the EP considered and recorded the applicability according to the type of person with incontinence, the care setting(s) and the intended user(s) following the KPI template (Supplementary Data D). The applicability of local/national frameworks for KPI implementation, such as the LPZ, an annual independent measurement tool of care quality in the Dutch healthcare sector [14], and the RAI-MDS, an assessment tool utilised in long-term care homes and care at home in over 35 countries [15], are suggested for each KPI . The use of local/national frameworks was suggested as a means of facilitating KPI implementation and integration into continence care ecosystems.

**Table 3:** Core KPI template

KPI template	
KPI title	- Exact title of the KPI
Description	- Description of the KPI
Target population	- Description of who the KPI is relevant for
Rationale	- Indication of the rationale for measuring the KPI, including potential impacts to patient care
Care setting(s)	- Indication of the applicable care setting(s) to which this KPI would be most applicable according to the WHO long-term care definition
Intended user	- Indication of the stakeholder for whom the KPI would be the most useful, i.e. physician, nurse, caregiver, person with incontinence, payer etc.
Monitoring	- Indication of who will monitor the KPI, how often and whether the KPI is incentivised
Reporting	- Indication of how often the KPI will be reported and by whom, including their level of involvement in / knowledge of toileting and containment strategies
Definition of success	- Indication of the definition of success for the KPI to inform progress towards a best standard of care
Ease of measurement	- Indication of the feasibility of measuring this KPI in a defined setting (High, Medium, Low)
Scale	- Indication of the scale at which the KPI can be implemented and used i.e. at the local care unit, regional, national, multi-national or global level
Data source(s)	- Indication of what data should be collected to measure and report the KPI, including from which date source(s)

**Table 4:** Selected core KPIs

User types				
Type of KPI		Care independent	Care dependent can express	Care dependent cannot express
Structure	Clinical	Proportion of staff with the skills to perform a continence assessment and prescribe a toileting and containment strategy		
Process	Clinical	Proportion of persons with incontinence in receipt of pads with a documented assessment and formulation of a toileting and containment strategy		
		Mean number of days from referral to assessment for persons with incontinence who require a toileting and containment strategy		
		Proportion of persons whose toileting and containment strategy is reviewed		
		Proportion of persons with incontinence who receive education on toileting and containment strategies*		
	QoL	Proportion of persons with incontinence deemed eligible for a toileting and containment strategy who are offered a choice of product type following assessment of incontinence*		
Outcome	Clinical		Proportion of care dependent persons with incontinence in receipt of a toileting and containment strategy who are able to independently manage their incontinence	
			Proportion of persons with incontinence and Incontinence Associated Dermatitis (IAD) who receive a toileting and containment strategy	
		Proportion of persons with incontinence with an indwelling catheter to manage incontinence		
		Proportion of persons with incontinence managed with a toileting and containment strategy who report "good" or "acceptable" levels of access and support to toilet facilities in their daily life		
	QoL	Persons with incontinence managed with a toileting and containment strategy who report sustained or improved emotional well-being*		
		Proportion of persons managing incontinence with a toileting and containment strategy who are either able to remain in work or take up work		
			Proportion of caregiving relatives of persons with incontinence who report an acceptable level of emotional well-being	
	Economic	Cost of hospital admissions and re-admissions related to poor management with toileting and containment strategies for incontinence		

**Key for user applicability selection**

Care independent = KPI can be applied to measure care for independent users of toileting and management with containment products

Care dependent CAN express themselves = KPI can be applied to measure care for care dependent users who can express the need to toilet and manage containment product

Care dependent CAN NOT express themselves = KPI can be applied to measure care for care dependent users who cannot express the need to toilet and manage containment product ( * information or choice might be given to or data may be gathered via a proxy, i.e. family relative, carer, etc.)

## Results

### Scoping review

From the searches of academic and grey literature, 1,111 studies were returned, of which 260 were selected following a title relevancy check. Of the 260 studies, 89 were selected following a review of the abstract and the full article was investigated. A total of 158 potential KPI titles were identified from the full review of 89 academic and grey literature sources (Supplementary Fig. A).

### Prioritisation criteria

The EP applied the prioritisation criteria to the long list of 158 KPI titles and then collectively reviewed these to reach a consensus on a prioritised short list of 41 KPI titles (Supplementary Fig. A).

### Stakeholder engagement

Of the 17 quantitative and qualitative questions, 56 KPI titles (18 clinical, 23 QoL and 15 economic) were suggested as additional KPI titles by the 58 participants (Supplementary Fig. B). These additional KPIs were considered during the EP workshop for inclusion into the final suite of KPIs.

### Expert panel workshop

During the EP face-to-face workshop, the short list of 41 KPI titles and the suggested additional 56 KPI titles were considered and discussed to form a refined short list of 35 KPI titles (Supplementary Figs. A, B).

### Delphi panel selection of core KPIs

The EP selected 14 KPIs from the refined short list of 35 KPI titles based on the selection criteria (Supplementary Fig. A).

### Validation of the core KPIs

Each of the 14 core KPIs was drafted in detail according to the KPI template and validated by the EP (Tables 3 and 4).

### Core KPIs arising from the scoping review, stakeholder engagement and moderated panel discussion

The EP considered the results of the scoping review and stakeholder engagement exercise as an integrated body of evidence. Discussions concentrated on considering all results regardless of their original source. The resulting core KPIs are outlined below, categorised by domain and KPI type (Table 1). Outcome KPI measured the success of a specific aspect of a toileting and containment strategy, and process/structure KPIs measure elements of a process or structure that supported a toileting and containment strategy (Supplementary Data D).

#### Clinical domain

##### Proportion of staff with the skills to perform a continence assessment and prescribe a toileting and containment strategy

A measure of the proportion of staff within a defined care setting with the skills, as specified by any relevant national competency standards, to provide a continence assessment and prescribe a toileting and containment strategy (structure).

##### Proportion of persons with incontinence in receipt of pads with a documented assessment and formulation of a toileting and containment strategy

A measure of the number of persons with incontinence who have obtained a documented continence assessment and formulated toileting and containment strategy within a timeframe that adheres to local protocols, if available, or a recommended timeframe of 4 weeks, expressed as a proportion of all eligible patients with bladder/bowel incontinence within the service setting (process).

##### Mean number of days from referral to assessment for persons with incontinence who require a toileting and containment strategy

A measure of the mean number of days from referral to assessment for persons with incontinence who require a toileting and containment strategy, for a given population and point in time (process).

##### Proportion of persons whose toileting and containment strategy is reviewed

A measure of the number of persons with incontinence who receive a review of their toileting and containment strategy (e.g. face-to-face for initial assessment or face-to-face or call/online for follow up/review), divided by the number of persons with incontinence who do not receive a review for more than a year (process).

##### Proportion of persons with incontinence who receive education on toileting and containment strategies

A patient-reported outcome measure of the proportion of persons with incontinence who receive education on toileting and containment strategies through educational materials, training and/or guidance, for a given population and point in time (process).

##### Proportion of persons with incontinence with an indwelling catheter to manage incontinence

A measure of the number of persons with incontinence with indwelling catheters, divided by the total population in that care setting. Note that this KPI was recognised by the EP as an indicator of poor continence care (outcome).

##### Proportion of care-dependent persons with incontinence managed with a toileting and containment strategy who are able to independently manage their incontinence

A measure of the number of care-dependent persons with incontinence managed with a toileting and containment strategy able to independently manage their continence with limited oversight by a clinician or caregiver, divided by the number of care-dependent persons with incontinence managed with a toileting and containment strategy for a given population and point in time (outcome).

##### Proportion of persons with incontinence and incontinence-associated dermatitis who receive a toileting and containment strategy

A measure of the persons with incontinence managed with a combination of toileting and containment products who have incontinence-associated dermatitis, for a given population and a point in time expressed as a proportion of all persons with incontinence managed with toileting and containment products (outcome).

##### Proportion of persons with incontinence managed with a toileting and containment strategy who report “good” or “acceptable” levels of access and support to toilet facilities in their daily life

A measure of persons with incontinence who report “good” or “acceptable” levels of access to toilet facilities, or support from caregivers, clinicians and family members to access toilet facilities in their daily life, for a given setting and population expressed as a proportion of the total number of patients/residents managed with toileting and containment products (outcome).

#### Quality of life

##### Proportion of persons with incontinence deemed eligible for a toileting and containment strategy who are offered a choice of product type following assessment of incontinence

A measure of the proportion of persons with incontinence, eligible for a toileting and containment strategy, who are provided with information on available containment product types and given the opportunity to state a preference following assessment (process).

##### Persons with incontinence managed with a toileting and containment strategy who report sustained or improved emotional well-being

A measure of the proportion of persons with incontinence who report sustained or improved emotional well-being captured by a validated quality of life questionnaire, the requirements of which are detailed in Supplementary Data D, expressed as the number of persons reporting emotional well-being above a threshold as defined by the organisation implementing the KPI. Domains of intent include: ability to maintain relationships with family and friends, comfort with sexuality, ability to travel, ability to wear preferred clothing, satisfaction with caregiver relationship, ability to preserve self-dignity, ability to manage incontinence with confidence (outcome).

##### Proportion of persons managing incontinence with a toileting and containment strategy who are able to either remain in work or take up work

A measure of persons with incontinence managed with a toileting and containment strategy who are able to either remain in employment or take up employment (full time or part time) for a given population and point in time expressed as a proportion of all persons with incontinence managed with a toileting and containment strategy who are eligible/desire to be in work or voluntary work (outcome).

##### Proportion of caregiving relatives of persons with incontinence who report an acceptable level of emotional well-being

A measure of caregiving relatives of care-dependent persons with incontinence and self-reported emotional well-being, captured through a questionnaire, the requirements of which are detailed in Supplementary Data D. Domains of interest include: the ability to maintain relationships with family and friends, the ability to cope as a caregiver, satisfaction with the level of support available, the impact of caregiving on physical health (outcome).

#### Economic

##### Cost of hospital admissions and re-admissions related to poor management with toileting and containment strategies for incontinence

A measure of the cost of hospital admissions and re-admissions to acute care emergency services related to poor toileting and containment strategy management (e.g. indwelling catheters, pressure ulcers, urinary tract infections, incontinence-associated dermatitis, harmful falls) expressed as an annual figure per service provider (outcome).

## Discussion

This study describes the development of a set of KPIs designed to measure outcomes for the management of urinary and faecal incontinence based upon toileting and containment strategies. Following a scoping review and broad stakeholder engagement, a long list of potential KPIs was refined into a set of 14 core KPIs. Each KPI was carefully detailed through expert consensus. Each KPI requires integration into existing national, local and provider-based quality frameworks for effective utilisation. Recognising the importance of high-quality continence care across the clinical, QoL and economic domains, each aspect was considered in conjunction with the KPI applicability matrix (Table 1). The resulting KPIs are intended to be broadly applicable to all adults with bladder or bowel incontinence and be suitable for incorporation into national or local quality frameworks. Development of the KPI underwent a rigorous process, informed by available evidence and reinforced by patient and caregiver input in an attempt to measure what truly matters to patients [16]. The resulting set of 14 KPIs cover Donabedian’s domains of structure, process and outcome [17] and, by their nature vary in terms of ease of incorporation and measurement. The wider set of 35 KPI (Supplementary Table C) are intended to supplement the core set, according to the needs of the commissioning provider or payer. As with any measures, these will only be effective if tested in the field and used. Implementation of performance measures in the area of continence care has, to date been patchy, with some surgical registries reporting on surgical outcome [18], but, to the best of our knowledge, there has been no systematic attempt to measure the quality of care other than that from 2006 to 2010 in England and Wales [9, 19]. It is hoped that these KPIs will prove of utility and value in quality assurance and, by the use of audit and feedback, promote quality improvement.

It is challenging to create a suite of KPIs that are internationally applicable to user types, care settings and multiple stakeholders with an interest in continence management. The study addressed this difficulty with its mixed methods approach, using the results of a scoping review of academic and grey literature, prioritising the results with the EP by consensus and augmenting findings with a stakeholder engagement exercise. Not all KPIs are directly relevant or applicable to all of the three defined user types, care settings and all stakeholders, and will require the user’s discretion in tailoring to specific needs. To improve continence care delivery by measuring toileting and containment strategy the KPI require the adaptation of existing local and national frameworks to facilitate KPI implementation tailored to the needs of a variety of care settings and stakeholders, including payers, providers, professionals, and patients and their caregivers.

There are limitations to this study. First, in the scoping review, non-English references were excluded. Therefore, it is possible that insights and findings that could have informed the development of the KPIs were missed. Second, although KPIs were informed by published evidence, this was often lacking, and expert consensus had to serve as a replacement where this was the case. Of the 12 identified stakeholder groups of KPI users, only 5 were represented on the EP, although the subsequent stakeholder engagement exercise included a broad swathe of participants involved at all levels of continence care. Finally, there is a scarcity of literature on continence care in developing countries. The balance of evidence largely came from North America and Europe and this may in itself have led to unavoidable bias.

## Conclusions

This study, using robust methods, has defined 14 core KPIs for integration into quality frameworks to facilitate delivery of high-quality continence care. Implementation via existing national, local and provider-based quality frameworks is encouraged to embed the core KPI into continence care provision. Core KPIs should, where possible, be monitored, reported and incentivised to measure and improve high-quality continence care. If implemented, the resulting benchmarking data will facilitate improvement of care delivery and has the potential to inform value-based procurement and provision of continence services.

## Electronic supplementary material

Supplementary Fig. A Prioritisation criteria (XLSX 48 kb)

Supplementary Fig. B Participants in the broad stakeholder engagement exercise (PPTX 3090 kb)

Table A Prioritised list of 158 KPI titles (DOCX 13.7 kb)

Table B Broad stakeholder engagement exercise key themes. C. Refined short list of 35 KPI titles. D. Detailed core KPI (PPTX 5.54 MB)
