# Supplementary material for: Development of quality outcome indicators to improve the quality of urinary and faecal continence care
Source: Int Urogynecol J. 2018 Oct 16;30(1):23–32. doi: 10.1007/s00192-018-3768-2 (PMC6514083; doi:10.1007/s00192-018-3768-2)
Supplement: Supplementary file 2 — Participants in the broad stakeholder engagement exercise (PPTX 3090 kb) [file 192_2018_3768_MOESM2_ESM.pptx]

## Slide 1
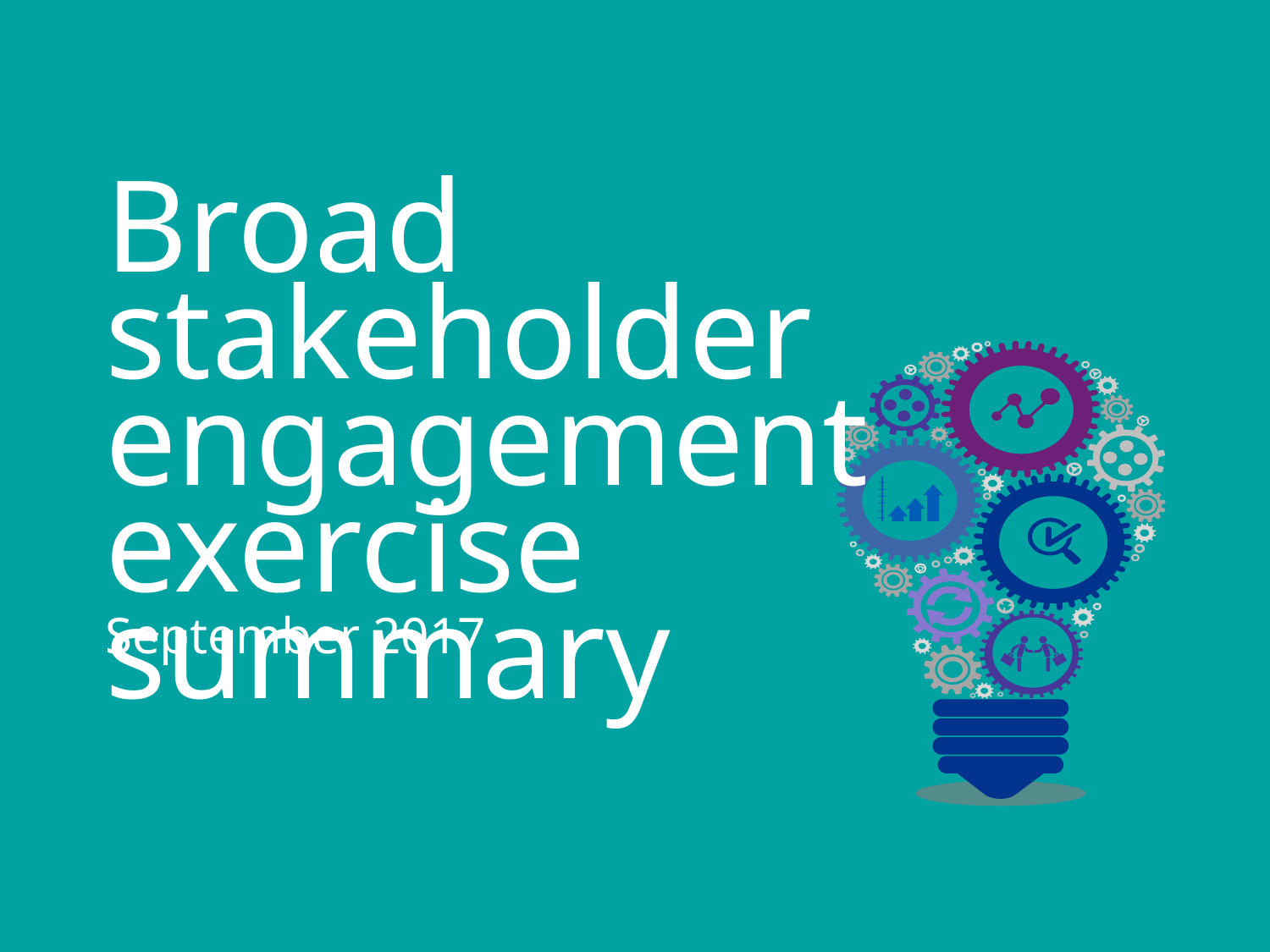

# Broad stakeholder engagement exercise summary
September 2017

## Slide 2
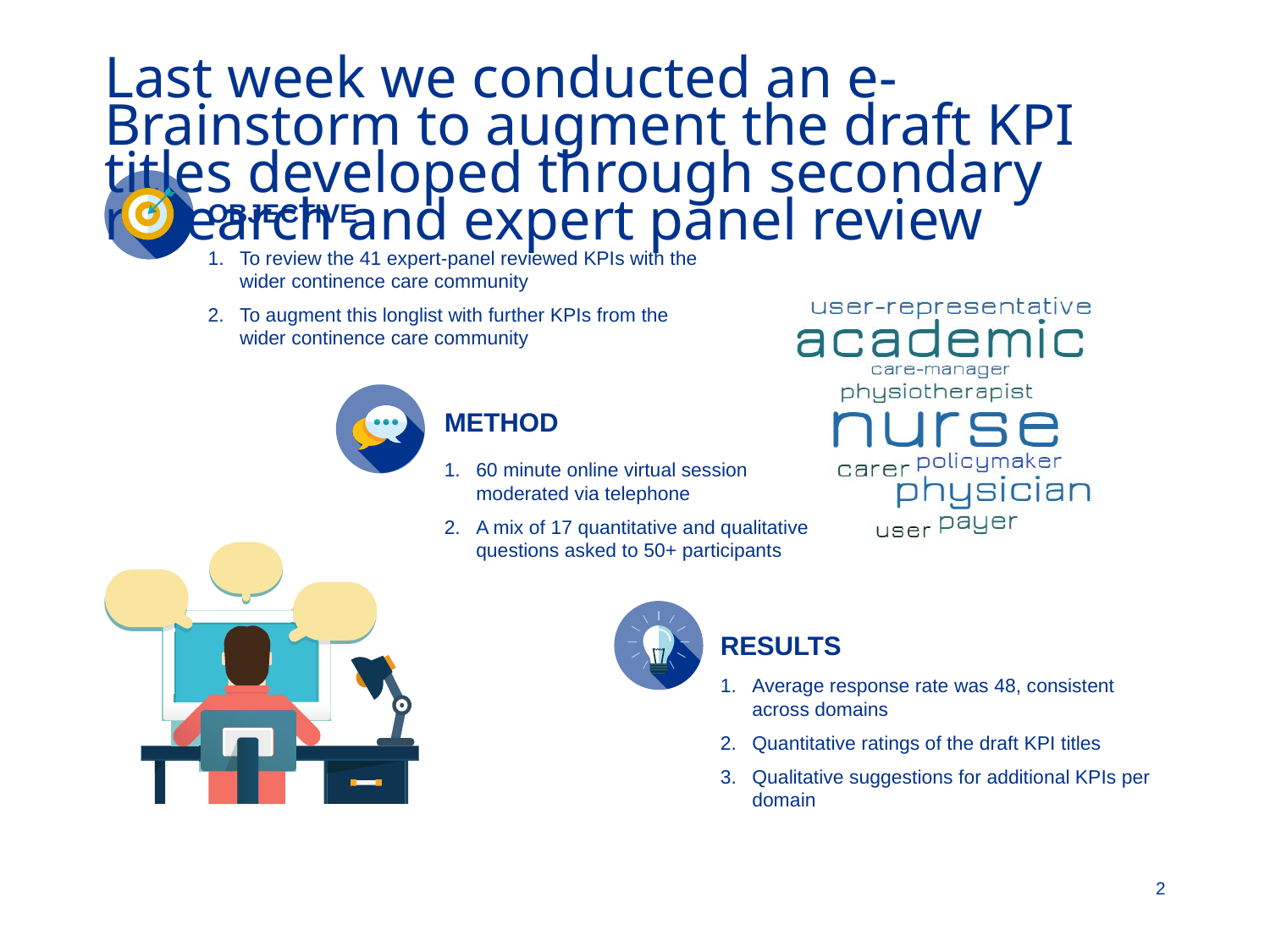

# Last week we conducted an e-Brainstorm to augment the draft KPI titles developed through secondary research and expert panel review
OBJECTIVE
To review the 41 expert-panel reviewed KPIs with the wider continence care community
To augment this longlist with further KPIs from the wider continence care community
METHOD
60 minute online virtual session moderated via telephone
A mix of 17 quantitative and qualitative questions asked to 50+ participants
RESULTS
Average response rate was 48, consistent across domains
Quantitative ratings of the draft KPI titles
Qualitative suggestions for additional KPIs per domain

## Slide 3
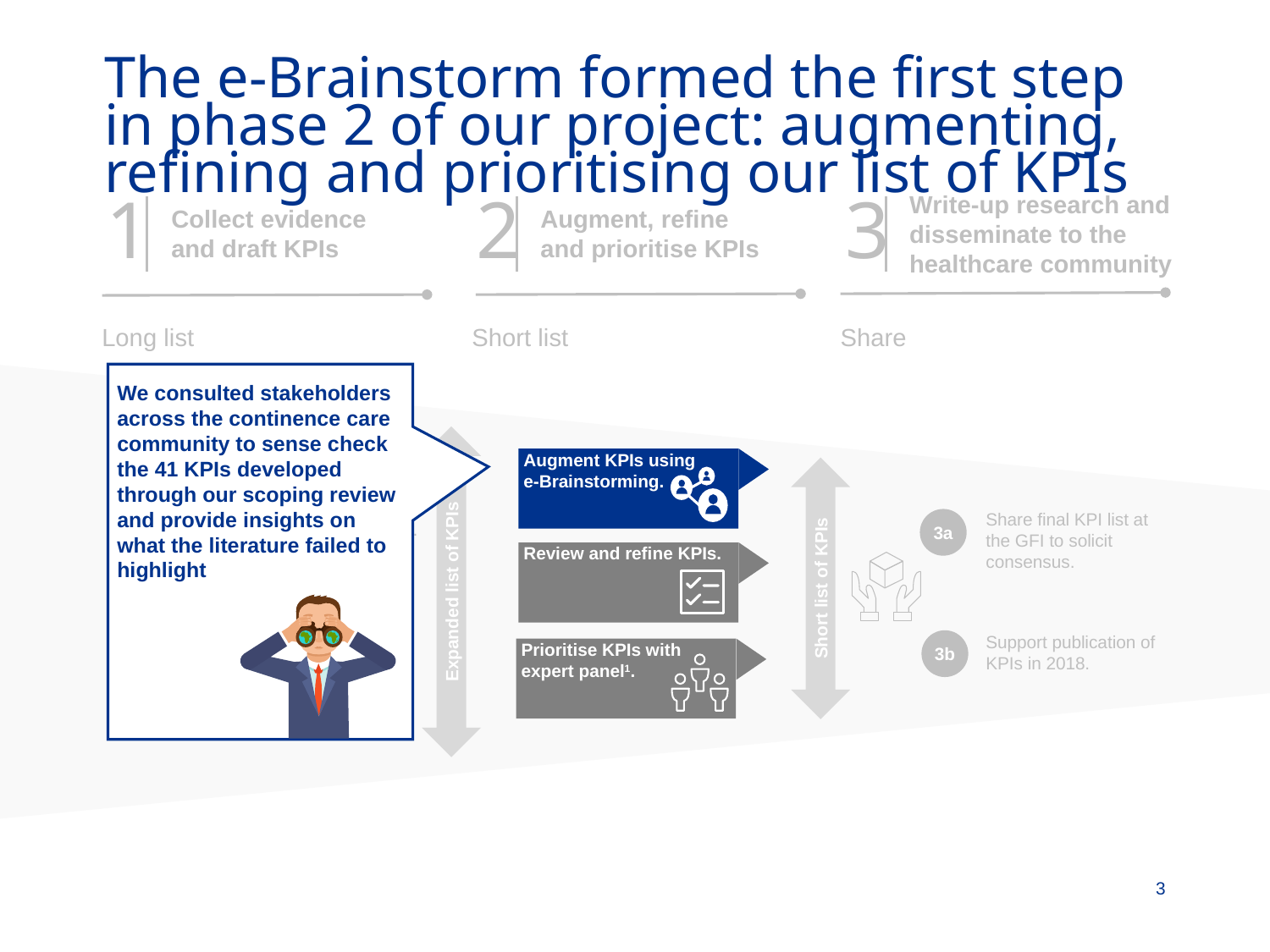

# The e-Brainstorm formed the first step in phase 2 of our project: augmenting, refining and prioritising our list of KPIs
2
3
1
Collect evidence and draft KPIs
Augment, refine and prioritise KPIs
Write-up research and disseminate to the healthcare community
Long list
Short list
Share
We consulted stakeholders across the continence care community to sense check the 41 KPIs developed through our scoping review and provide insights on what the literature failed to highlight
Expanded list of KPIs
Review literature to identify existing performance indicators to measure outcomes for the management of containment / containment strategy.
Expand on identified KPIs with the expert panel.
Draft list of KPIs to measure outcomes for the management of containment / containment strategy per patient profile.
Augment KPIs using e-Brainstorming.
Short list of KPIs
Share final KPI list at the GFI to solicit consensus.
3a
Review and refine KPIs.
Support publication of KPIs in 2018.
3b
Prioritise KPIs with expert panel1.

## Slide 4
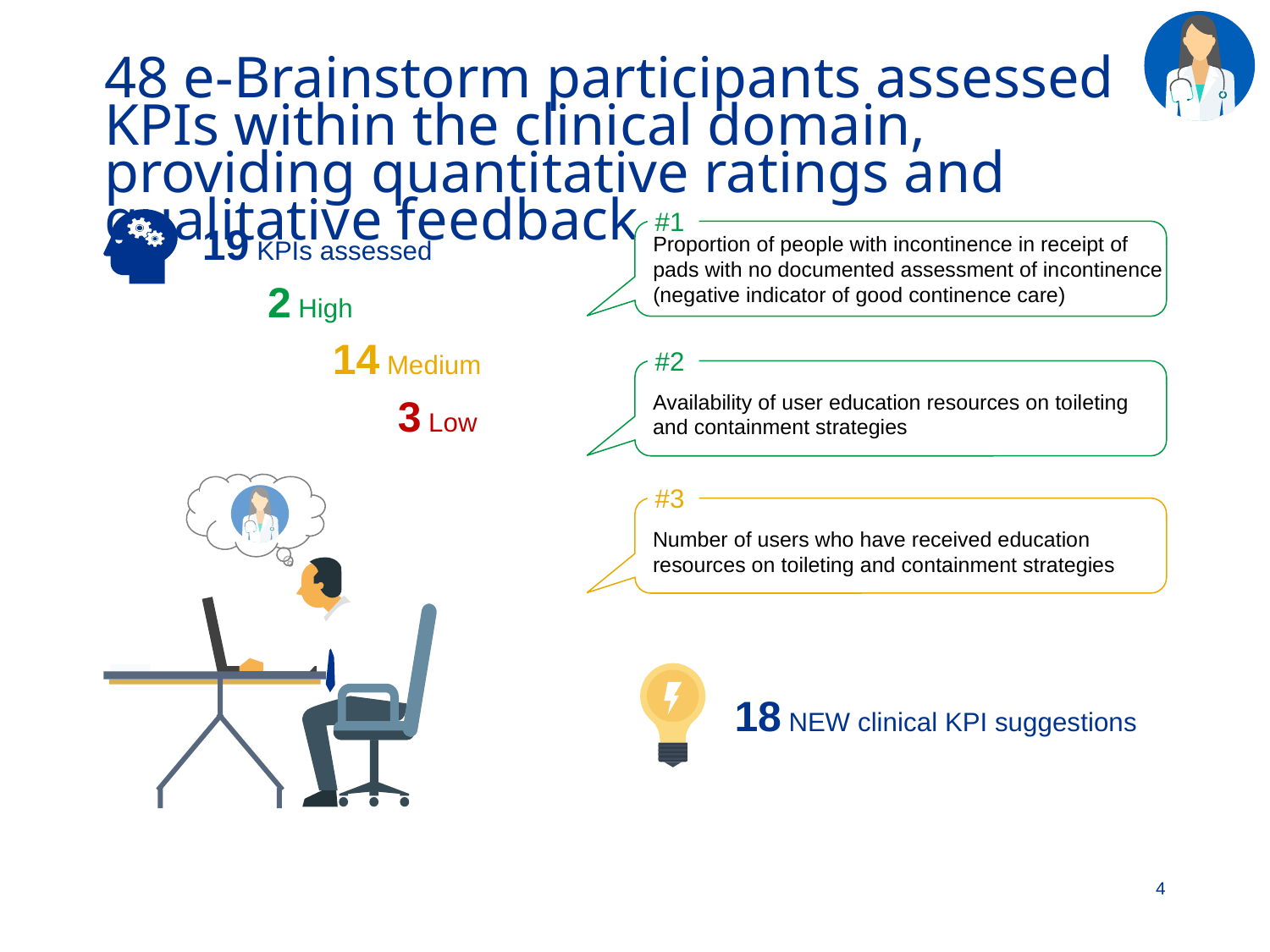

# 48 e-Brainstorm participants assessed KPIs within the clinical domain, providing quantitative ratings and qualitative feedback
#1
Proportion of people with incontinence in receipt of pads with no documented assessment of incontinence (negative indicator of good continence care)
19 KPIs assessed
2 High
14 Medium
#2
Availability of user education resources on toileting and containment strategies
3 Low
#3
Number of users who have received education resources on toileting and containment strategies
18 NEW clinical KPI suggestions

## Slide 5
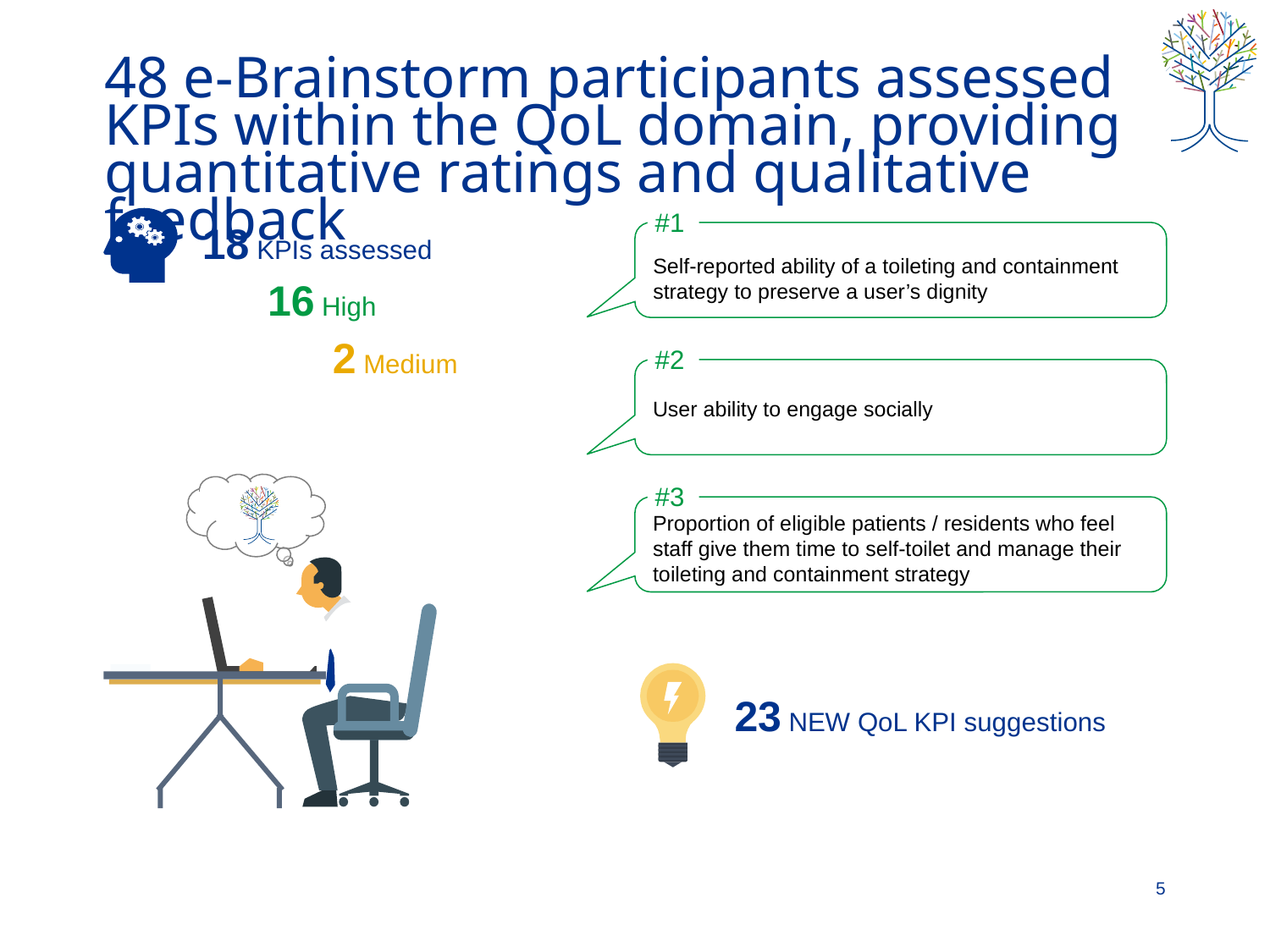

# 48 e-Brainstorm participants assessed KPIs within the QoL domain, providing quantitative ratings and qualitative feedback
#1
Self-reported ability of a toileting and containment strategy to preserve a user’s dignity
18 KPIs assessed
16 High
2 Medium
#2
User ability to engage socially
#3
Proportion of eligible patients / residents who feel staff give them time to self-toilet and manage their toileting and containment strategy
23 NEW QoL KPI suggestions

## Slide 6
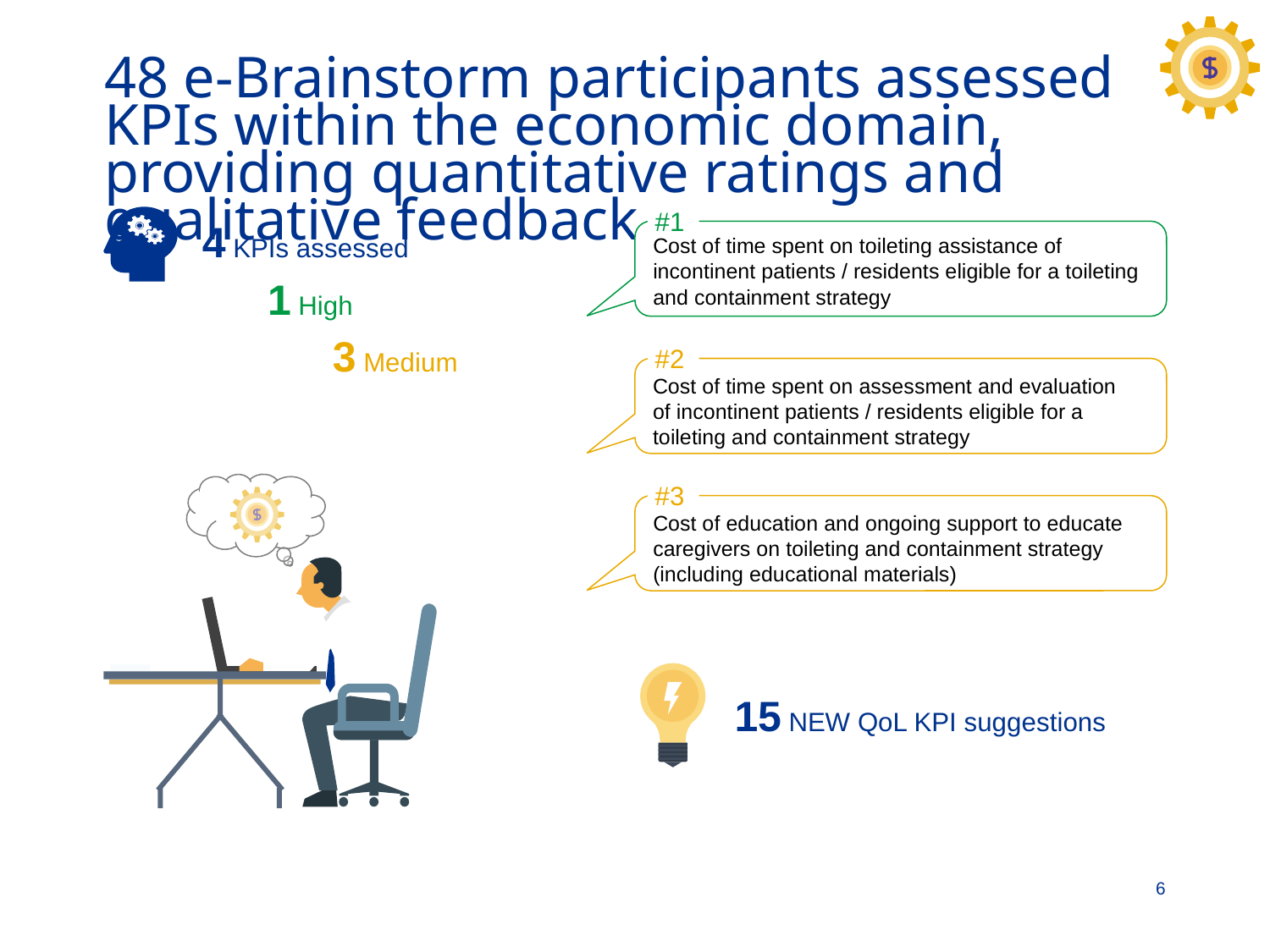

# 48 e-Brainstorm participants assessed KPIs within the economic domain, providing quantitative ratings and qualitative feedback
#1
Cost of time spent on toileting assistance of incontinent patients / residents eligible for a toileting and containment strategy
4 KPIs assessed
1 High
3 Medium
#2
Cost of time spent on assessment and evaluation of incontinent patients / residents eligible for a toileting and containment strategy
#3
Cost of education and ongoing support to educate caregivers on toileting and containment strategy (including educational materials)
15 NEW QoL KPI suggestions

## Slide 7
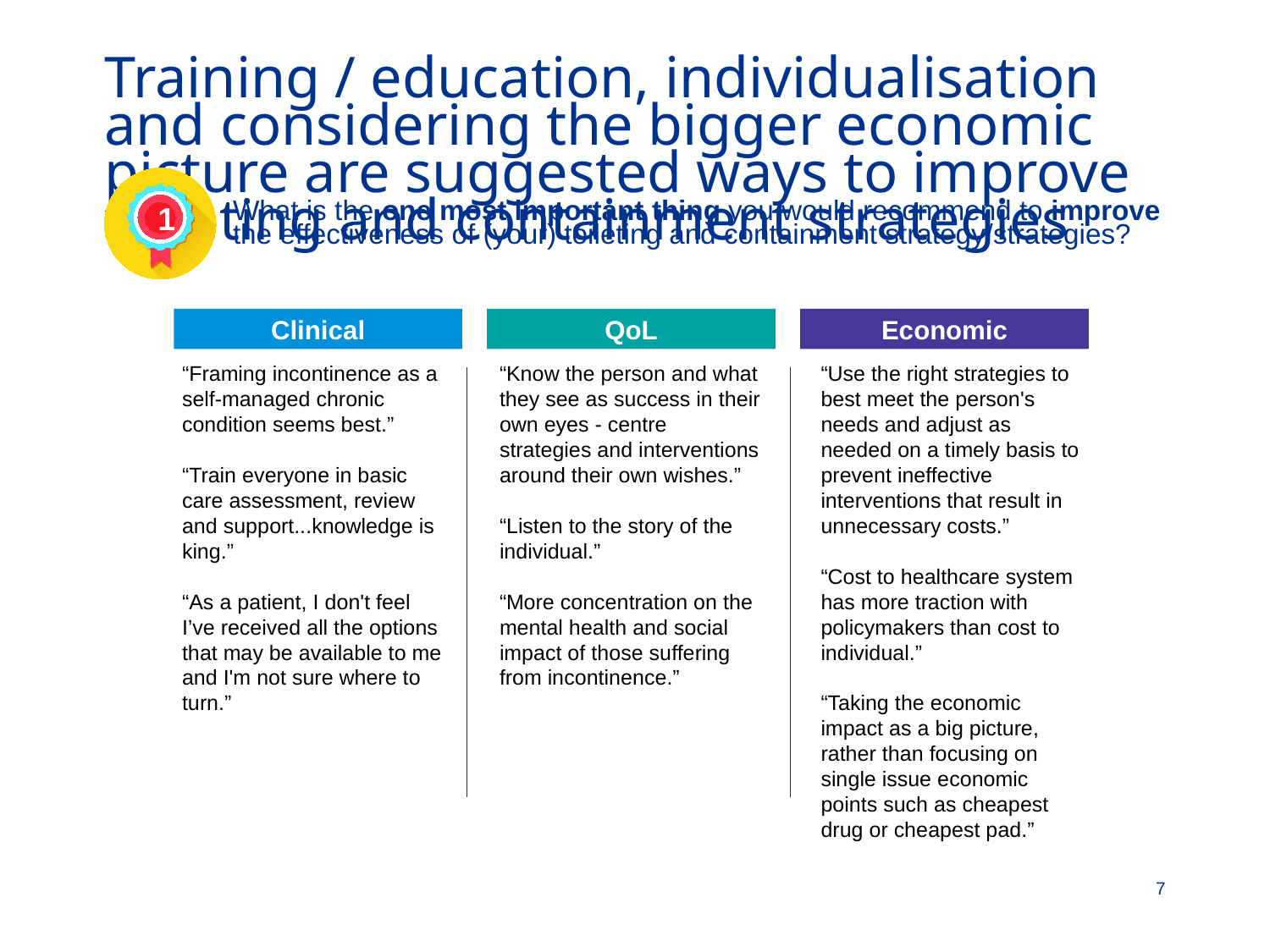

# Training / education, individualisation and considering the bigger economic picture are suggested ways to improve toileting and containment strategies
1
What is the one most important thing you would recommend to improve the effectiveness of (your) toileting and containment strategy/strategies?
Economic
Clinical
QoL
“Framing incontinence as a self-managed chronic condition seems best.”
“Train everyone in basic care assessment, review and support...knowledge is king.”
“As a patient, I don't feel I’ve received all the options that may be available to me and I'm not sure where to turn.”
“Know the person and what they see as success in their own eyes - centre strategies and interventions around their own wishes.”
“Listen to the story of the individual.”
“More concentration on the mental health and social impact of those suffering from incontinence.”
“Use the right strategies to best meet the person's needs and adjust as needed on a timely basis to prevent ineffective interventions that result in unnecessary costs.”
“Cost to healthcare system has more traction with policymakers than cost to individual.”
“Taking the economic impact as a big picture, rather than focusing on single issue economic points such as cheapest drug or cheapest pad.”

## Slide 8
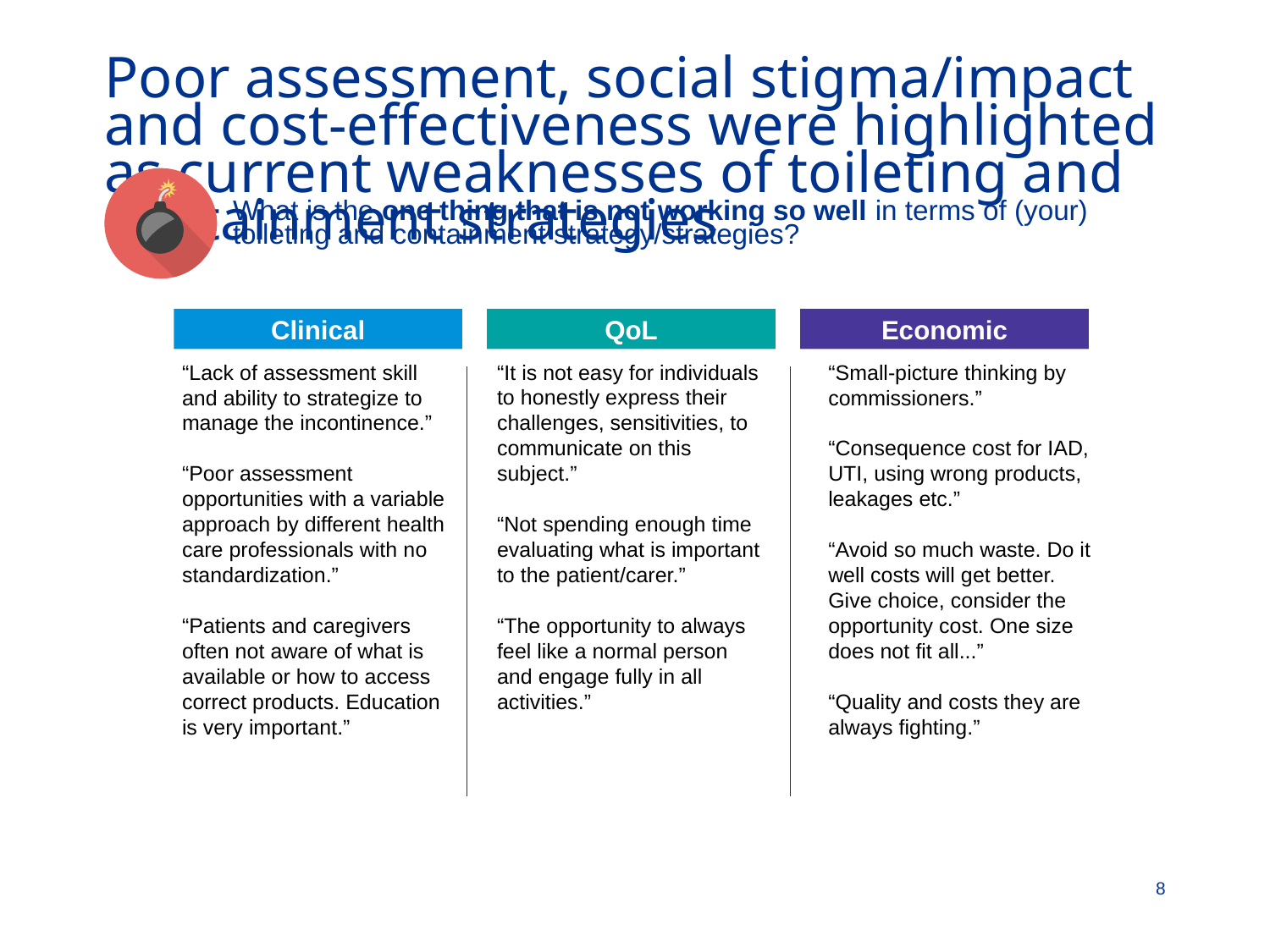

# Poor assessment, social stigma/impact and cost-effectiveness were highlighted as current weaknesses of toileting and containment strategies
What is the one thing that is not working so well in terms of (your) toileting and containment strategy/strategies?
Economic
Clinical
QoL
“Lack of assessment skill and ability to strategize to manage the incontinence.”
“Poor assessment opportunities with a variable approach by different health care professionals with no standardization.”
“Patients and caregivers often not aware of what is available or how to access correct products. Education is very important.”
“It is not easy for individuals to honestly express their challenges, sensitivities, to communicate on this subject.”
“Not spending enough time evaluating what is important to the patient/carer.”
“The opportunity to always feel like a normal person and engage fully in all activities.”
“Small-picture thinking by commissioners.”
“Consequence cost for IAD, UTI, using wrong products, leakages etc.”
“Avoid so much waste. Do it well costs will get better. Give choice, consider the opportunity cost. One size does not fit all...”
“Quality and costs they are always fighting.”

## Slide 9
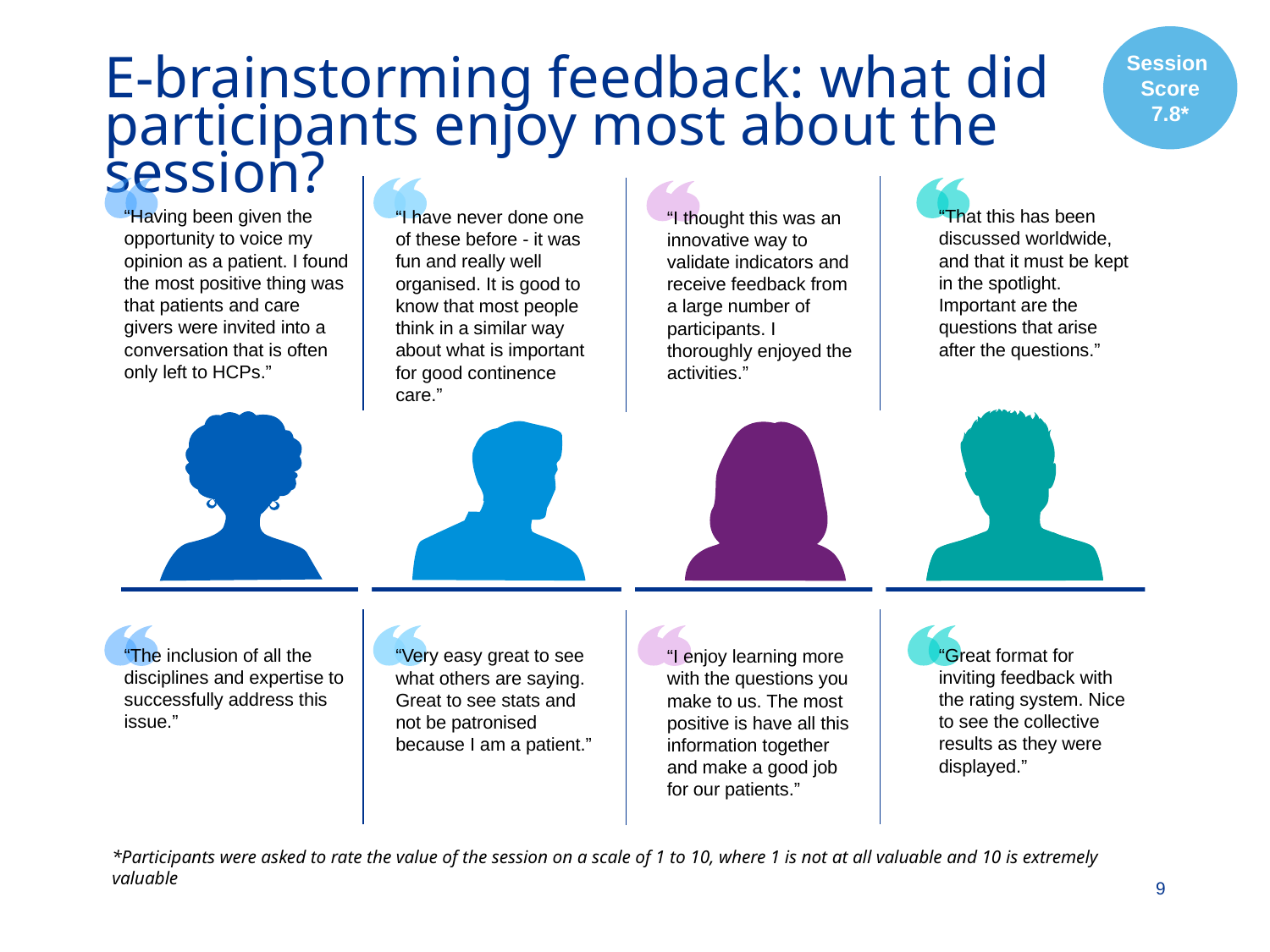

Session
Score
7.8*
# E-brainstorming feedback: what did participants enjoy most about the session?
“Having been given the opportunity to voice my opinion as a patient. I found the most positive thing was that patients and care givers were invited into a conversation that is often only left to HCPs.”
“That this has been discussed worldwide, and that it must be kept in the spotlight. Important are the questions that arise after the questions.”
“I have never done one of these before - it was fun and really well organised. It is good to know that most people think in a similar way about what is important for good continence care.”
“I thought this was an innovative way to validate indicators and receive feedback from a large number of participants. I thoroughly enjoyed the activities.”
“The inclusion of all the disciplines and expertise to successfully address this issue.”
“Great format for inviting feedback with the rating system. Nice to see the collective results as they were displayed.”
“Very easy great to see what others are saying. Great to see stats and not be patronised because I am a patient.”
“I enjoy learning more with the questions you make to us. The most positive is have all this information together and make a good job for our patients.”
*Participants were asked to rate the value of the session on a scale of 1 to 10, where 1 is not at all valuable and 10 is extremely valuable
