# Supplementary material for: Development of quality outcome indicators to improve the quality of urinary and faecal continence care
Source: Int Urogynecol J. 2018 Oct 16;30(1):23–32. doi: 10.1007/s00192-018-3768-2 (PMC6514083; doi:10.1007/s00192-018-3768-2)
Supplement: Supplementary file 3 — Prioritised list of 158 KPI titles (DOCX 13.7 kb) [file 192_2018_3768_MOESM3_ESM.docx]

| **Refined short list of 35 KPI titles** |
| --- |
| **DOMAIN: CLINICAL** |
| Proportion of people with incontinence in receipt of pads with no documented initial assessment of continence (negative indicator of good continence care) |
| Availability of user education resources on toileting and containment strategies |
| Proportion of users who have received education resources on toileting and containment strategies |
| Proportion of people managed by a toileting and containment strategy who report "good" or “acceptable” levels of access and support to toilet facilities in their daily life |
| Proportion of patients / residents with incontinence who are assessed as appropriate to receive a toileting and containment strategy |
| Proportion of patients / residents in receipt of a toileting and containment strategy who are able to independently manage their continence |
| Proportion of patients / residents measured at regular intervals with Incontinence Associated Dermatitis (IAD) who are managed with a combination of toileting and containment products |
| Proportion of patients / residents with an indwelling catheter to manage incontinence (negative indicator of good continence care) |
| Number of leakage accidents with products for containment of urinary and faecal incontinence |
| Proportion of people with incontinence in receipt of pads with no documented formulation of a toileting and containment strategy (negative indicator of good continence care) |
| Proportion of people managed by a toileting and containment strategy with access to holistic care when required (i.e. physiotherapist, occupational therapist, nurse) |
| Proportion of staff with the requisite skills to perform a continence assessment and provide a toileting and containment strategy |
| Proportion of patients / residents who receive regular face to face review of their toileting and containment strategy |
| Proportion of patients / residents who adhere to their toileting and containment strategy |
| Proportion of patients / residents measured at regular intervals managed by a toileting and containment strategy who experience Urinary Tract Infections (UTIs) |
| Mean number of days from referral to assessment for patients / residents with incontinence who require a toileting and containment strategy |
| Number of falls in patients / residents in receipt of toileting and containment strategies over a time period |
| Proportion of people admitted to institutional care every six months who receive a combination of toileting and containment products |
| Number of completed toiletings divided by total number of expected toiletings during the specified time period for care dependent patients / residents |
| **DOMAIN: QUALITY OF LIFE** |
| Proportion of people that report that their toileting and containment strategy preserves their dignity |
| Proportion of eligible patients / residents who feel staff give them time to self-toilet and manage their toileting and containment strategy |
| Self-reported emotional wellbeing measured by an updated PAD-PROM questionnaire (i.e. relationships, sexuality, ability to exercise, ability to travel, ability to wear preferred clothing, satisfaction with caregiver relationship) |
| Proportion of caregivers of patients / residents in receipt of a toileting and containment strategy who feel involved in toileting and containment strategy decision-making, where relevant |
| Proportion of patients / residents satisfied with toileting assistance and containment strategy |
| Proportion of people managing incontinence with a toileting and containment strategy who are either able to remain in work or take up work |
| A measure of the attainment of patient / resident specified outcome goals, within a population receiving toileting and containment strategies to manage continence |
| Proportion of patients / residents deemed eligible for a toileting and containment strategy who are offered a choice of product type following assessment of continence |
| Self-reported user ability to manage one’s continence with confidence |
| Proportion of cognitively impaired residents who exhibit responsive behaviours when attempting to deliver a toileting and containment strategy for maintenance of continence |
| Proportion of people who report that their toileting and containment strategy supports a good level of sleep |
| **DOMAIN: ECONOMIC** |
| Cost of toileting assistance of incontinent patients / residents eligible for a toileting and containment strategy |
| Cost of time spent on assessment and evaluation of incontinent patients / residents eligible for a toileting and containment strategy |
| Cost of personnel time spent to provide toileting and containment management (to include assessment, follow-up, bed changing and other daily management tasks) |
| Cost of hospital admissions for a particular clinical concern related to poor toileting and containment strategy management (i.e. UTI, IAD, harmful falls, blocked indwelling catheters, ulcers) |
| Saving cost of keeping people at home rather than in institutional care |
