# Supplementary material for: Development of quality outcome indicators to improve the quality of urinary and faecal continence care
Source: Int Urogynecol J. 2018 Oct 16;30(1):23–32. doi: 10.1007/s00192-018-3768-2 (PMC6514083; doi:10.1007/s00192-018-3768-2)

## Slide 1
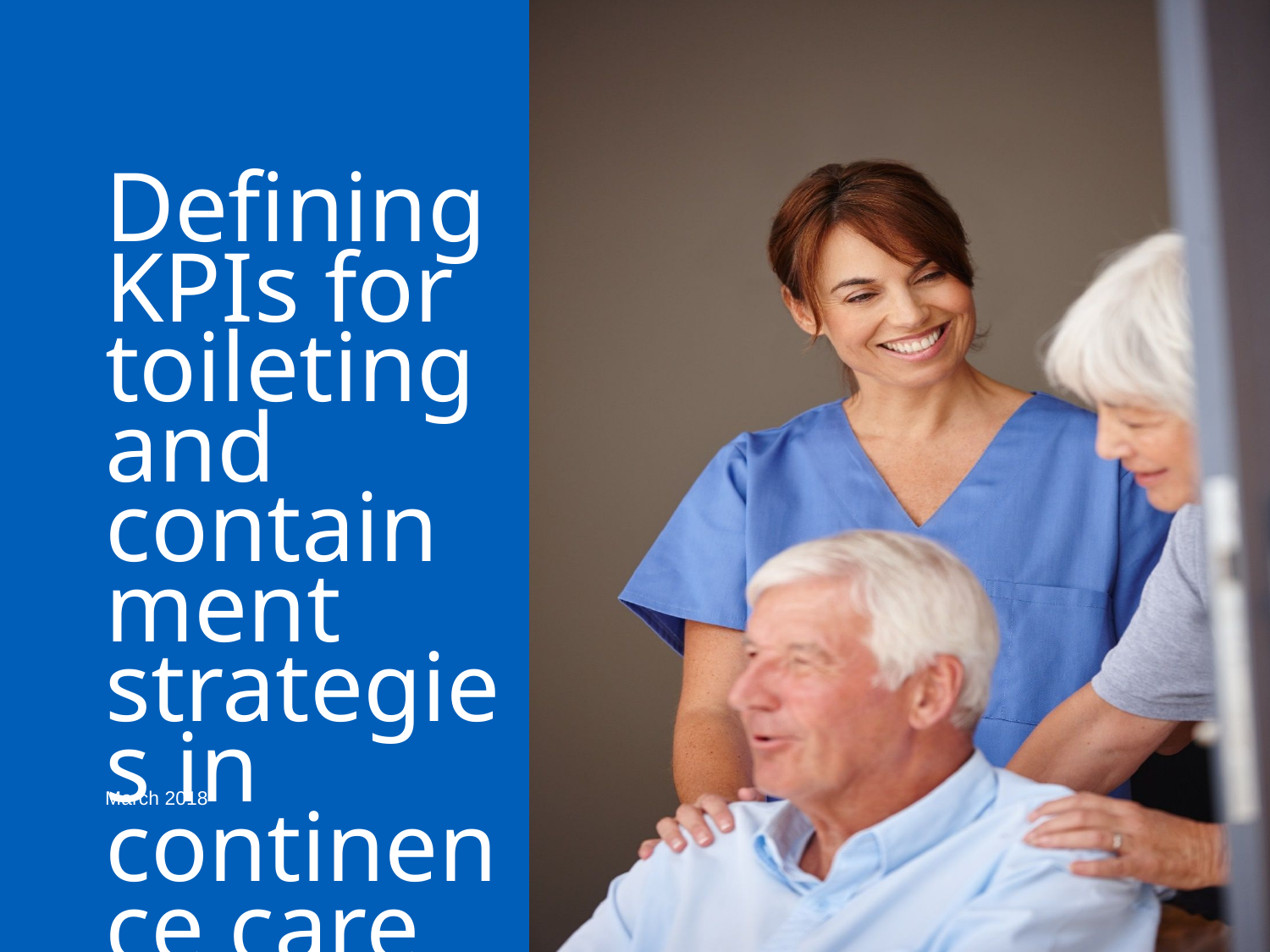

# Defining KPIs for toileting and containment strategies in continence care
March 2018

## Slide 2
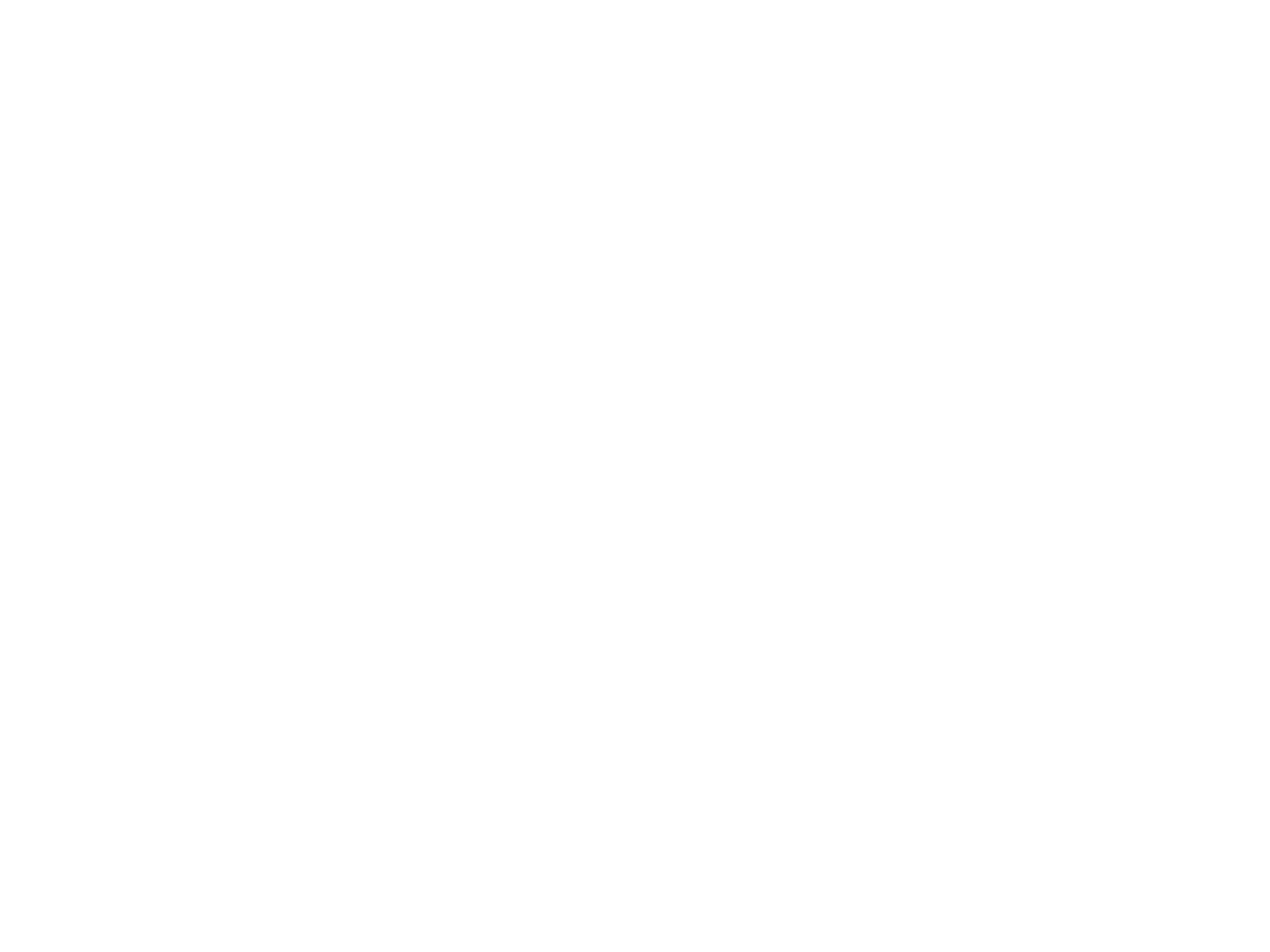

## Slide 3
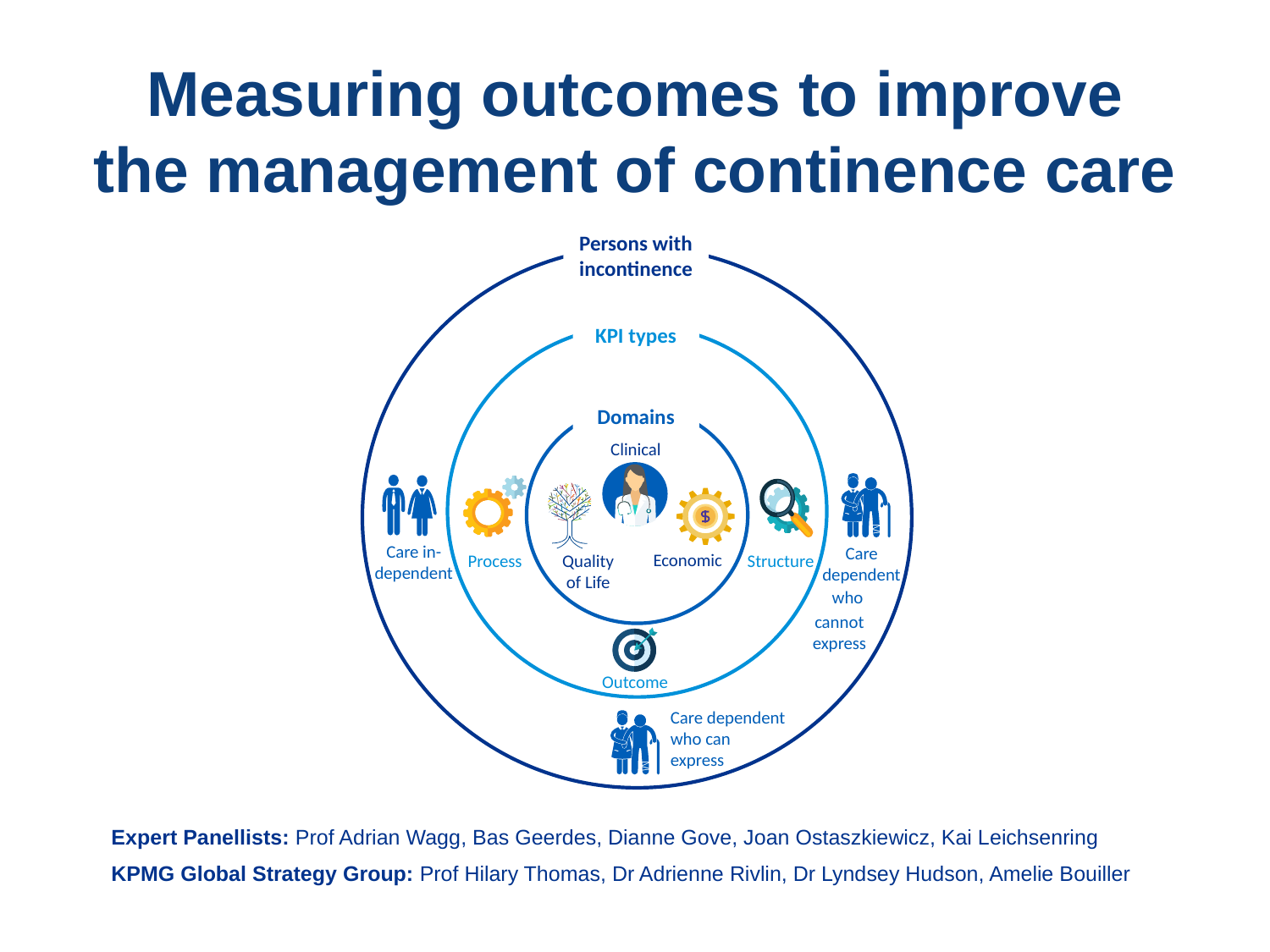

Measuring outcomes to improve the management of continence care
Persons with incontinence
KPI types
Domains
Clinical
Care in-dependent
Care dependent
Economic
Quality of Life
Process
Structure
who
cannot express
Outcome
Care dependent who can express
Expert Panellists: Prof Adrian Wagg, Bas Geerdes, Dianne Gove, Joan Ostaszkiewicz, Kai Leichsenring
KPMG Global Strategy Group: Prof Hilary Thomas, Dr Adrienne Rivlin, Dr Lyndsey Hudson, Amelie Bouiller

## Slide 4
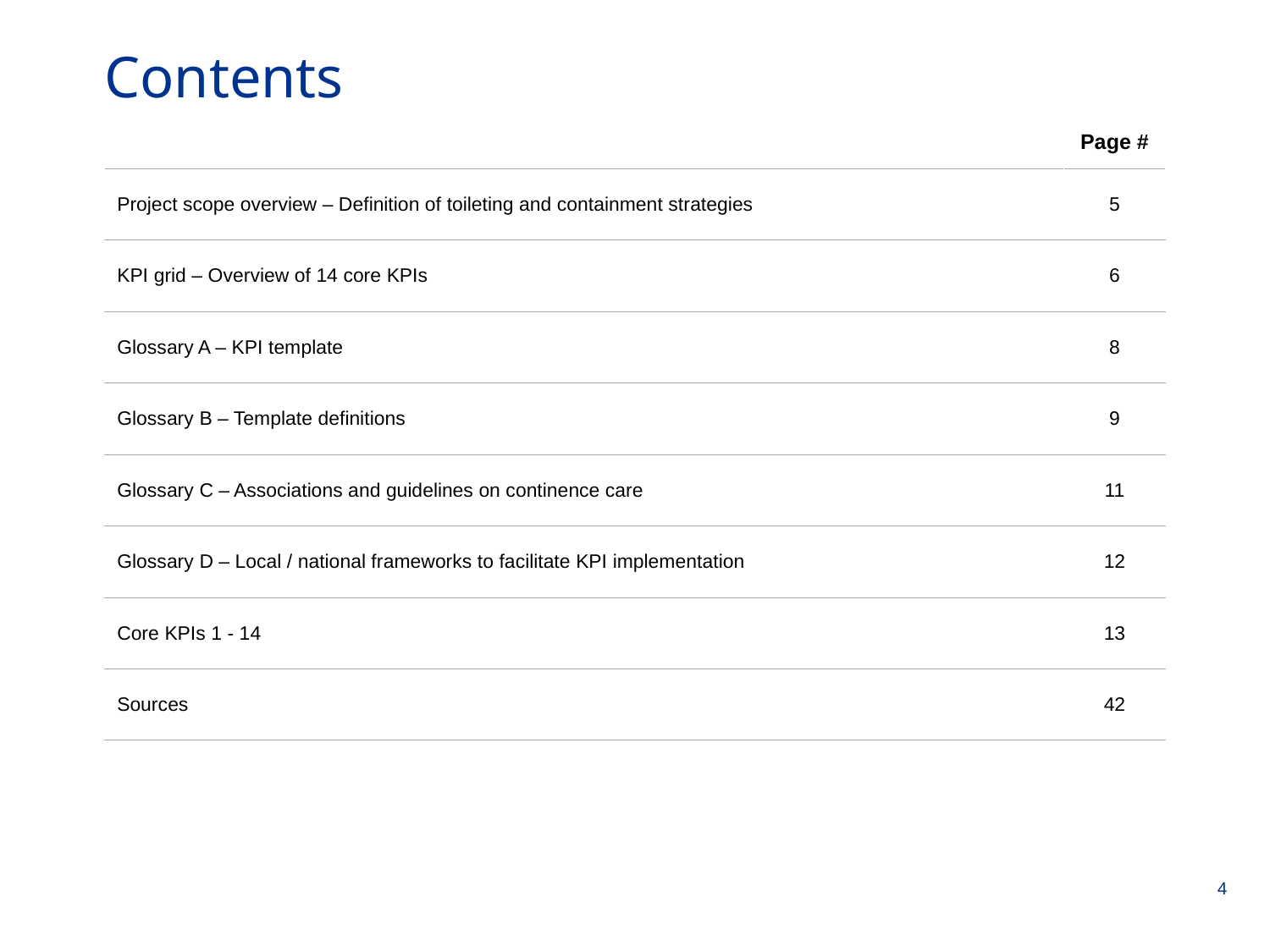

# Contents
| | Page # |
| --- | --- |
| Project scope overview – Definition of toileting and containment strategies | 5 |
| KPI grid – Overview of 14 core KPIs | 6 |
| Glossary A – KPI template | 8 |
| Glossary B – Template definitions | 9 |
| Glossary C – Associations and guidelines on continence care | 11 |
| Glossary D – Local / national frameworks to facilitate KPI implementation | 12 |
| Core KPIs 1 - 14 | 13 |
| Sources | 42 |

## Slide 5
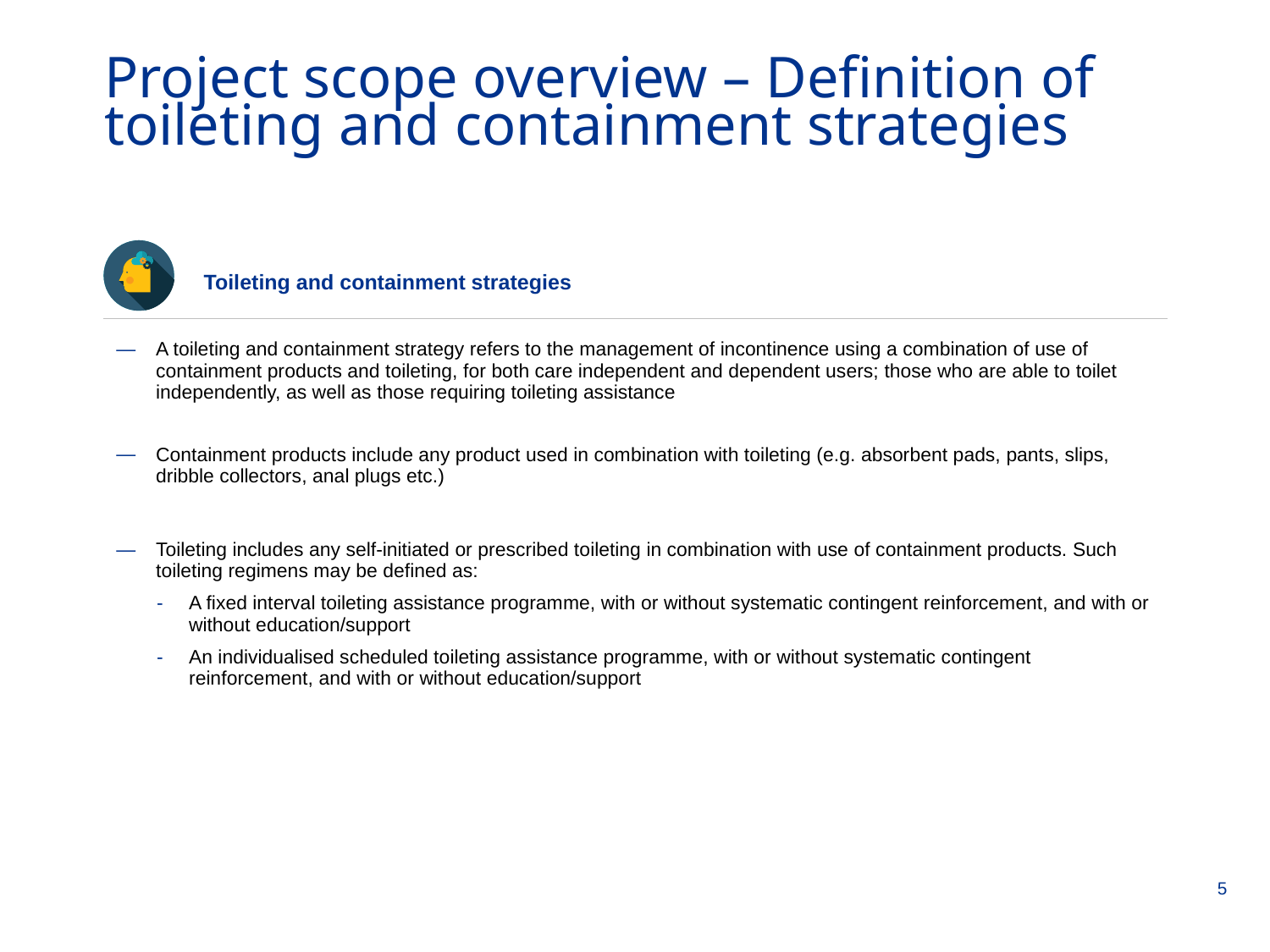

# Project scope overview – Definition of toileting and containment strategies
| Toileting and containment strategies |
| --- |
| A toileting and containment strategy refers to the management of incontinence using a combination of use of containment products and toileting, for both care independent and dependent users; those who are able to toilet independently, as well as those requiring toileting assistance |
| Containment products include any product used in combination with toileting (e.g. absorbent pads, pants, slips, dribble collectors, anal plugs etc.) |
| Toileting includes any self-initiated or prescribed toileting in combination with use of containment products. Such toileting regimens may be defined as: A fixed interval toileting assistance programme, with or without systematic contingent reinforcement, and with or without education/support An individualised scheduled toileting assistance programme, with or without systematic contingent reinforcement, and with or without education/support |
| |

## Slide 6
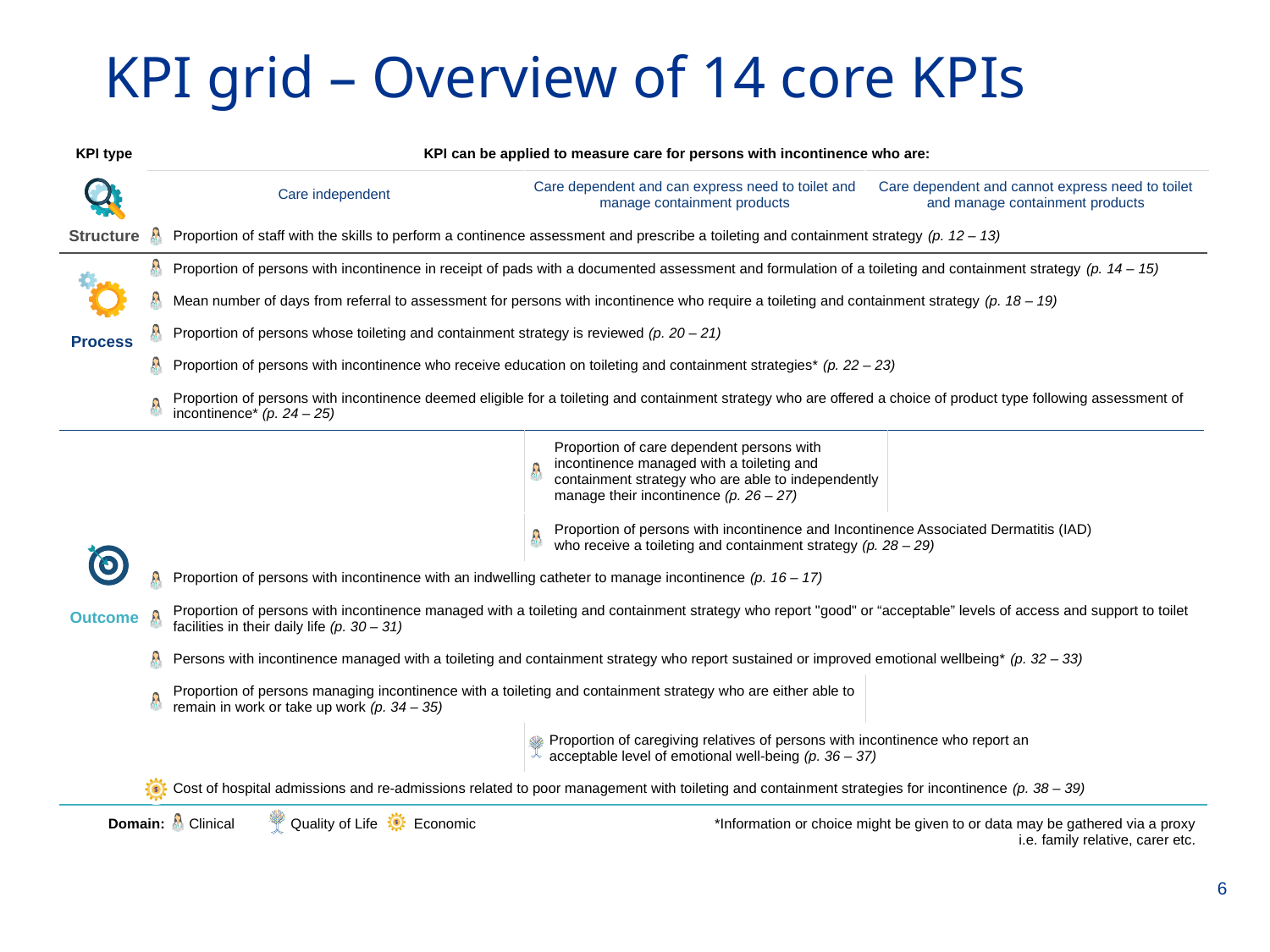

# KPI grid – Overview of 14 core KPIs
| KPI type | KPI can be applied to measure care for persons with incontinence who are: | | | |
| --- | --- | --- | --- | --- |
| | Care independent | Care dependent and can express need to toilet and manage containment products | Care dependent and cannot express need to toilet and manage containment products | |
| Structure | Proportion of staff with the skills to perform a continence assessment and prescribe a toileting and containment strategy (p. 12 – 13) | | | |
| Process | Proportion of persons with incontinence in receipt of pads with a documented assessment and formulation of a toileting and containment strategy (p. 14 – 15) | | | |
| | Mean number of days from referral to assessment for persons with incontinence who require a toileting and containment strategy (p. 18 – 19) | | | |
| | Proportion of persons whose toileting and containment strategy is reviewed (p. 20 – 21) | | | |
| | Proportion of persons with incontinence who receive education on toileting and containment strategies\* (p. 22 – 23) | | | |
| | Proportion of persons with incontinence deemed eligible for a toileting and containment strategy who are offered a choice of product type following assessment of incontinence\* (p. 24 – 25) | | | |
| Outcome | | Proportion of care dependent persons with incontinence managed with a toileting and containment strategy who are able to independently manage their incontinence (p. 26 – 27) | | |
| | | Proportion of persons with incontinence and Incontinence Associated Dermatitis (IAD) who receive a toileting and containment strategy (p. 28 – 29) | | |
| | Proportion of persons with incontinence with an indwelling catheter to manage incontinence (p. 16 – 17) | | | |
| | Proportion of persons with incontinence managed with a toileting and containment strategy who report "good" or “acceptable” levels of access and support to toilet facilities in their daily life (p. 30 – 31) | | | |
| | Persons with incontinence managed with a toileting and containment strategy who report sustained or improved emotional wellbeing\* (p. 32 – 33) | | | |
| | Proportion of persons managing incontinence with a toileting and containment strategy who are either able to remain in work or take up work (p. 34 – 35) | | | |
| | | Proportion of caregiving relatives of persons with incontinence who report an acceptable level of emotional well-being (p. 36 – 37) | | |
| | Cost of hospital admissions and re-admissions related to poor management with toileting and containment strategies for incontinence (p. 38 – 39) | | | |
Domain: Clinical Quality of Life Economic
*Information or choice might be given to or data may be gathered via a proxy i.e. family relative, carer etc.

## Slide 7
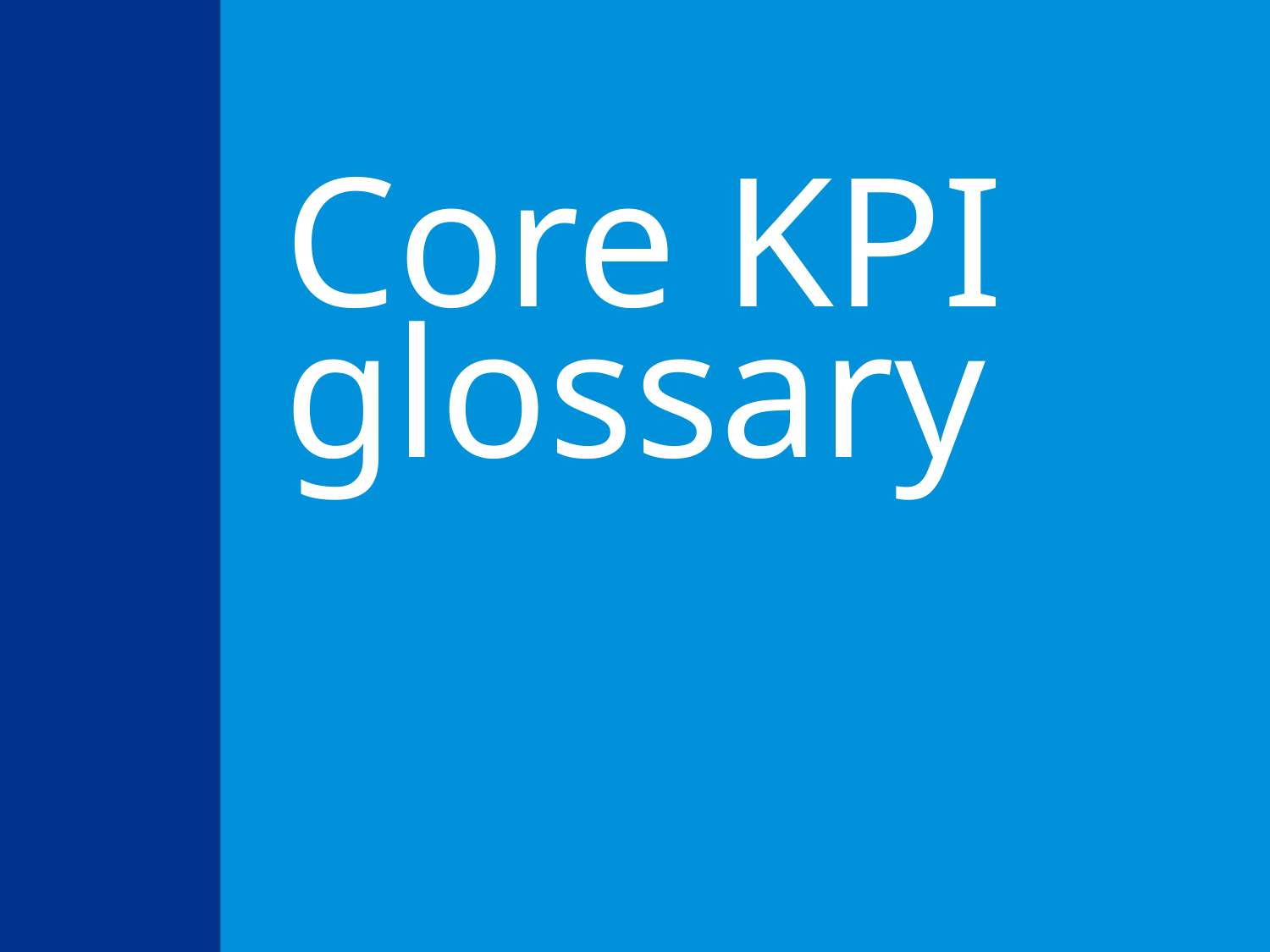

# Core KPI glossary

## Slide 8
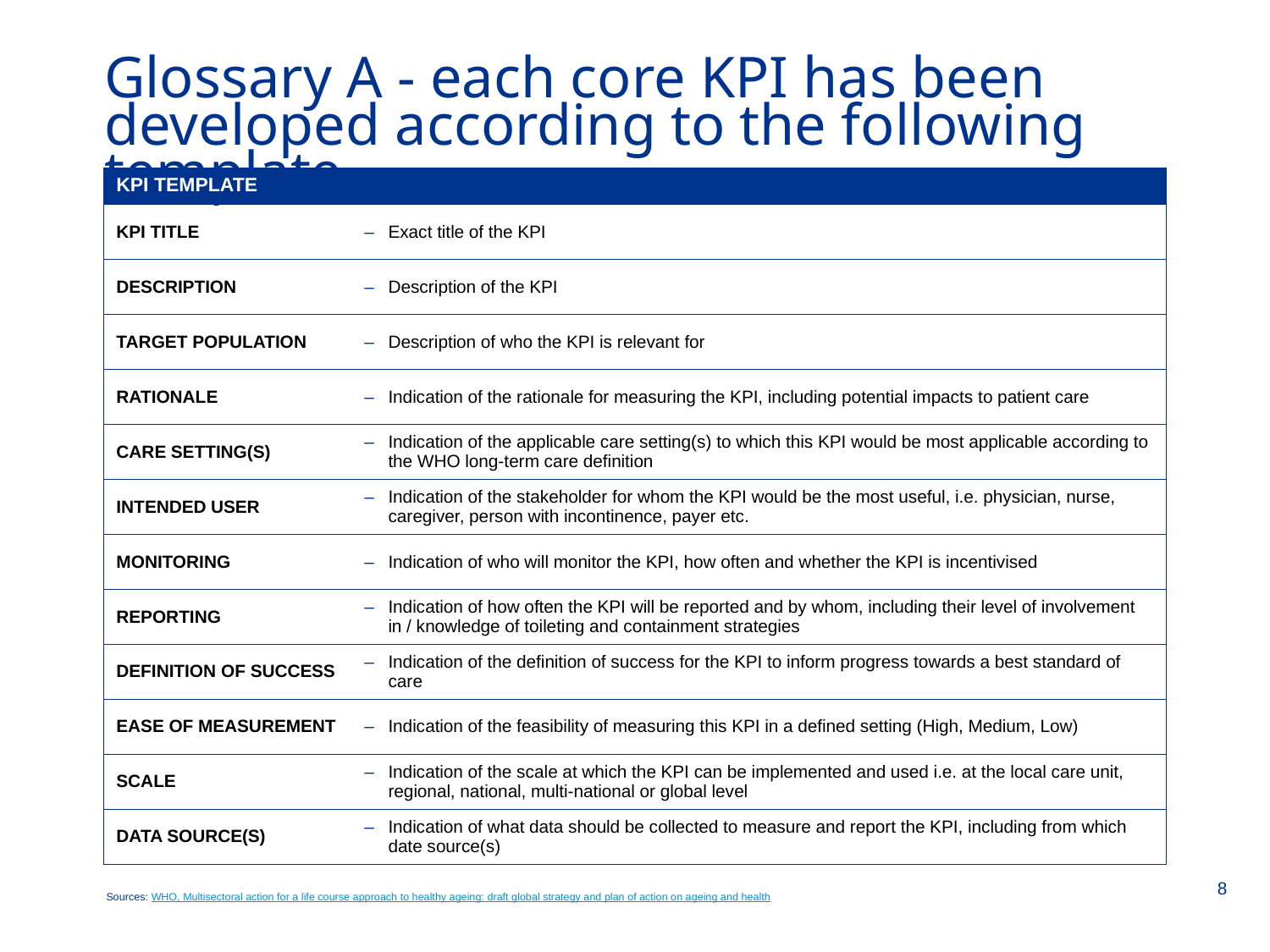

# Glossary A - each core KPI has been developed according to the following template
| KPI TEMPLATE | |
| --- | --- |
| KPI TITLE | Exact title of the KPI |
| DESCRIPTION | Description of the KPI |
| TARGET POPULATION | Description of who the KPI is relevant for |
| RATIONALE | Indication of the rationale for measuring the KPI, including potential impacts to patient care |
| CARE SETTING(S) | Indication of the applicable care setting(s) to which this KPI would be most applicable according to the WHO long-term care definition |
| INTENDED USER | Indication of the stakeholder for whom the KPI would be the most useful, i.e. physician, nurse, caregiver, person with incontinence, payer etc. |
| MONITORING | Indication of who will monitor the KPI, how often and whether the KPI is incentivised |
| REPORTING | Indication of how often the KPI will be reported and by whom, including their level of involvement in / knowledge of toileting and containment strategies |
| DEFINITION OF SUCCESS | Indication of the definition of success for the KPI to inform progress towards a best standard of care |
| EASE OF MEASUREMENT | Indication of the feasibility of measuring this KPI in a defined setting (High, Medium, Low) |
| SCALE | Indication of the scale at which the KPI can be implemented and used i.e. at the local care unit, regional, national, multi-national or global level |
| DATA SOURCE(S) | Indication of what data should be collected to measure and report the KPI, including from which date source(s) |
Sources: WHO, Multisectoral action for a life course approach to healthy ageing: draft global strategy and plan of action on ageing and health

## Slide 9
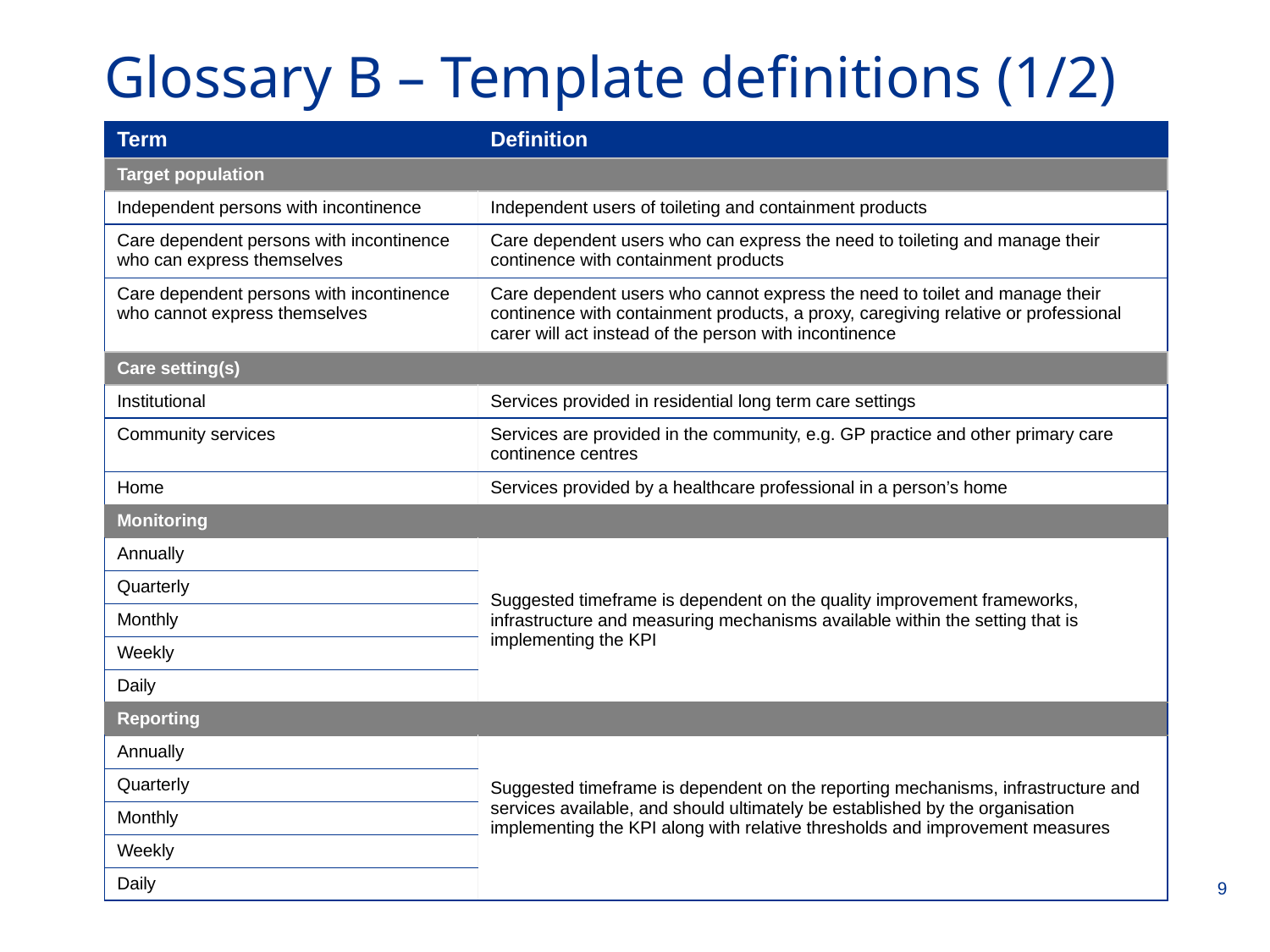

# Glossary B – Template definitions (1/2)
| Term | Definition |
| --- | --- |
| Target population | |
| Independent persons with incontinence | Independent users of toileting and containment products |
| Care dependent persons with incontinence who can express themselves | Care dependent users who can express the need to toileting and manage their continence with containment products |
| Care dependent persons with incontinence who cannot express themselves | Care dependent users who cannot express the need to toilet and manage their continence with containment products, a proxy, caregiving relative or professional carer will act instead of the person with incontinence |
| Care setting(s) | |
| Institutional | Services provided in residential long term care settings |
| Community services | Services are provided in the community, e.g. GP practice and other primary care continence centres |
| Home | Services provided by a healthcare professional in a person’s home |
| Monitoring | |
| Annually | Suggested timeframe is dependent on the quality improvement frameworks, infrastructure and measuring mechanisms available within the setting that is implementing the KPI |
| Quarterly | |
| Monthly | |
| Weekly | |
| Daily | |
| Reporting | |
| Annually | Suggested timeframe is dependent on the reporting mechanisms, infrastructure and services available, and should ultimately be established by the organisation implementing the KPI along with relative thresholds and improvement measures |
| Quarterly | |
| Monthly | |
| Weekly | |
| Daily | |

## Slide 10
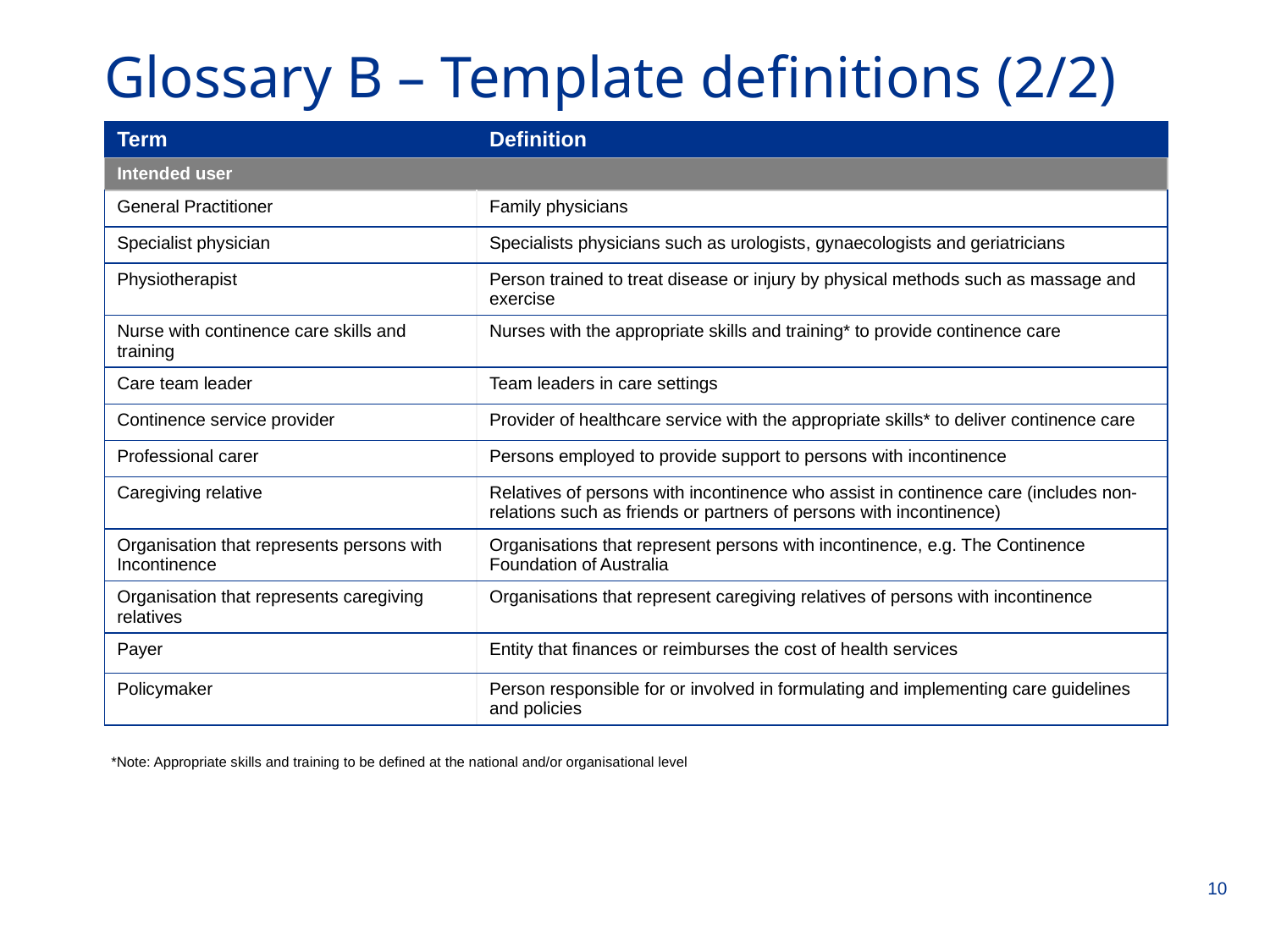

# Glossary B – Template definitions (2/2)
| Term | Definition |
| --- | --- |
| Intended user | |
| General Practitioner | Family physicians |
| Specialist physician | Specialists physicians such as urologists, gynaecologists and geriatricians |
| Physiotherapist | Person trained to treat disease or injury by physical methods such as massage and exercise |
| Nurse with continence care skills and training | Nurses with the appropriate skills and training\* to provide continence care |
| Care team leader | Team leaders in care settings |
| Continence service provider | Provider of healthcare service with the appropriate skills\* to deliver continence care |
| Professional carer | Persons employed to provide support to persons with incontinence |
| Caregiving relative | Relatives of persons with incontinence who assist in continence care (includes non-relations such as friends or partners of persons with incontinence) |
| Organisation that represents persons with Incontinence | Organisations that represent persons with incontinence, e.g. The Continence Foundation of Australia |
| Organisation that represents caregiving relatives | Organisations that represent caregiving relatives of persons with incontinence |
| Payer | Entity that finances or reimburses the cost of health services |
| Policymaker | Person responsible for or involved in formulating and implementing care guidelines and policies |
*Note: Appropriate skills and training to be defined at the national and/or organisational level

## Slide 11
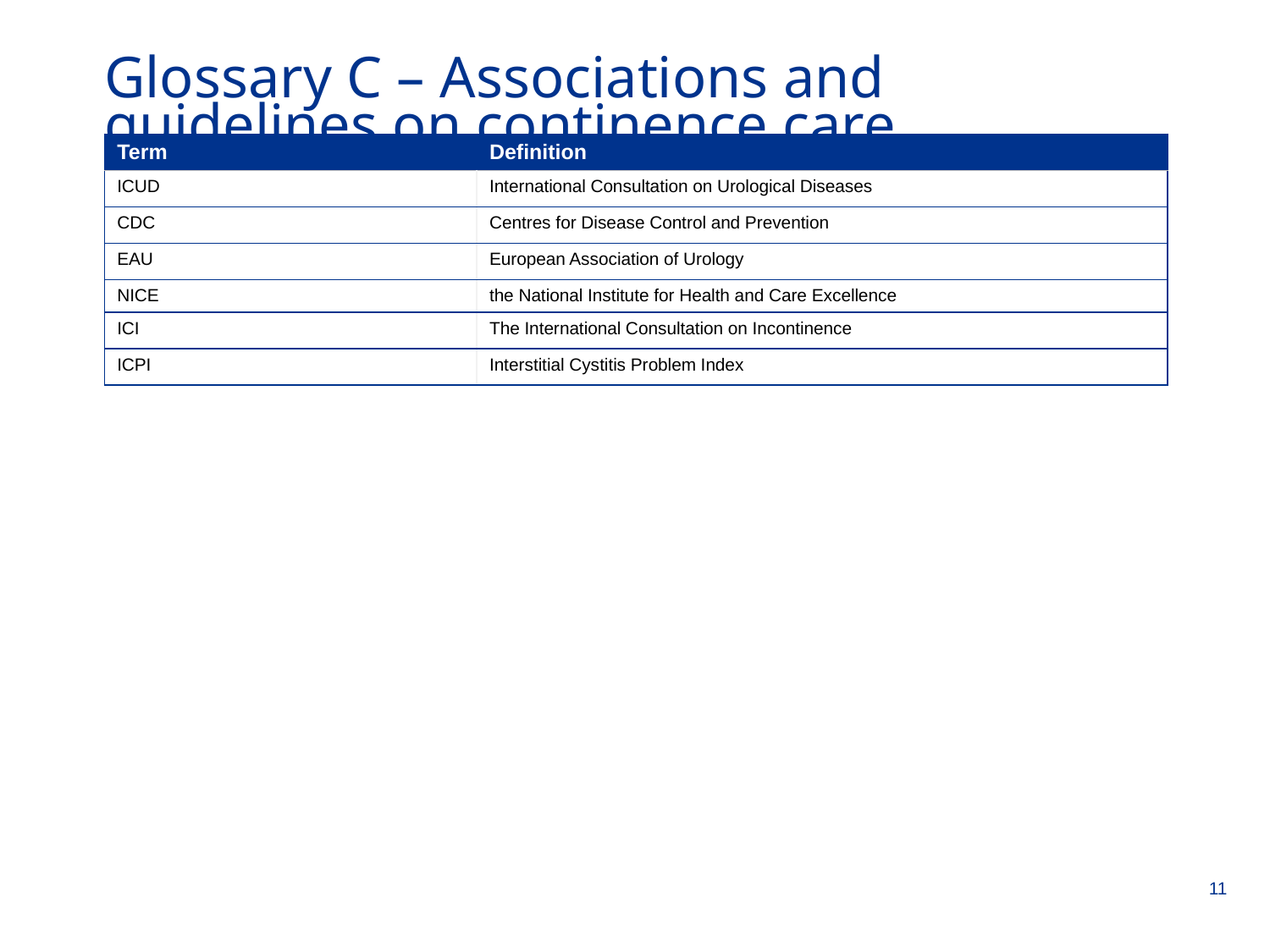

# Glossary C – Associations and guidelines on continence care
| Term | Definition |
| --- | --- |
| ICUD | International Consultation on Urological Diseases |
| CDC | Centres for Disease Control and Prevention |
| EAU | European Association of Urology |
| NICE | the National Institute for Health and Care Excellence |
| ICI | The International Consultation on Incontinence |
| ICPI | Interstitial Cystitis Problem Index |

## Slide 12
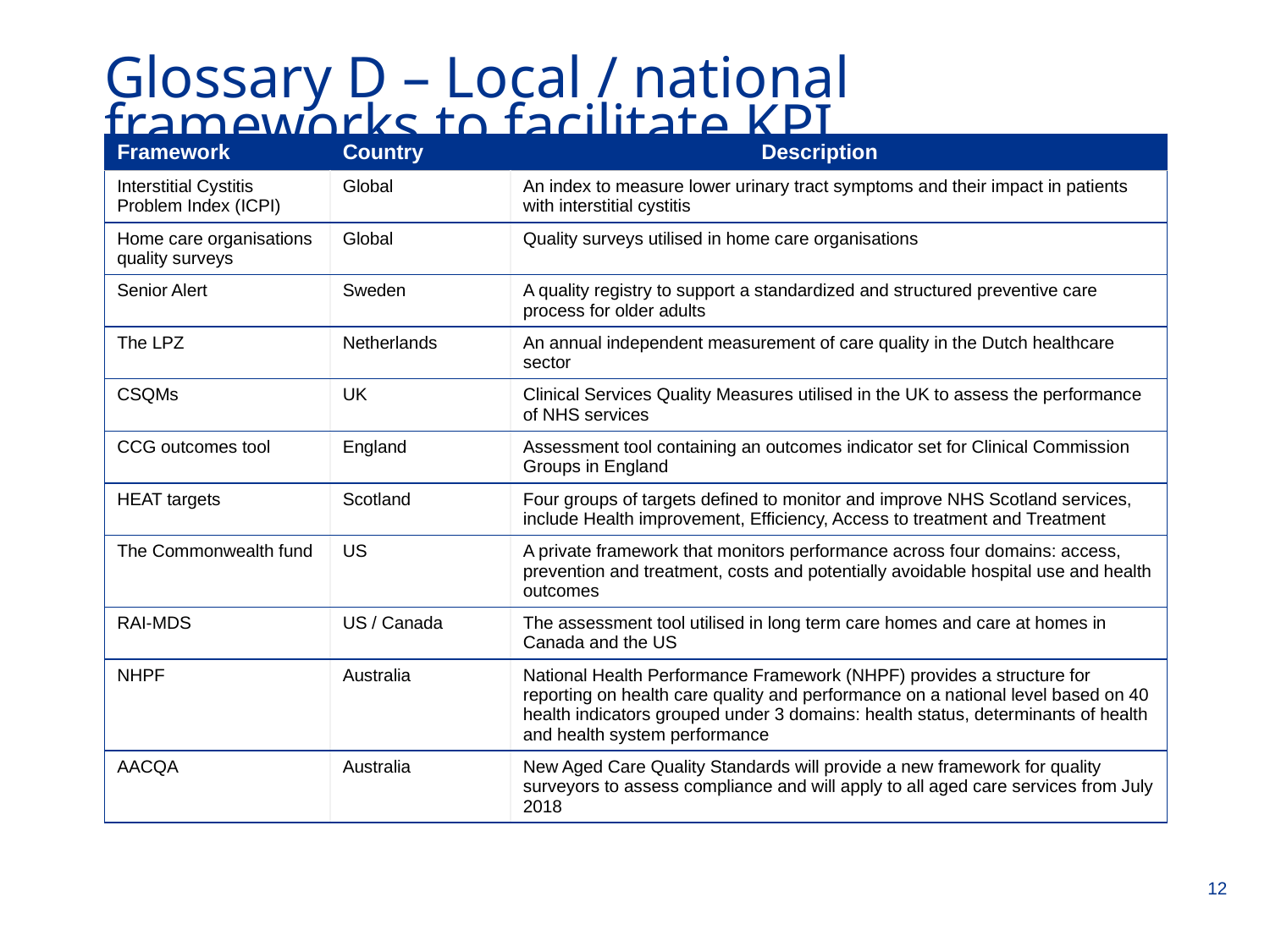

# Glossary D – Local / national frameworks to facilitate KPI implementation
| Framework | Country | | Description |
| --- | --- | --- | --- |
| Interstitial Cystitis Problem Index (ICPI) | Global | An index to measure lower urinary tract symptoms and their impact in patients with interstitial cystitis | |
| Home care organisations quality surveys | Global | Quality surveys utilised in home care organisations | |
| Senior Alert | Sweden | A quality registry to support a standardized and structured preventive care process for older adults | |
| The LPZ | Netherlands | An annual independent measurement of care quality in the Dutch healthcare sector | |
| CSQMs | UK | Clinical Services Quality Measures utilised in the UK to assess the performance of NHS services | |
| CCG outcomes tool | England | Assessment tool containing an outcomes indicator set for Clinical Commission Groups in England | |
| HEAT targets | Scotland | Four groups of targets defined to monitor and improve NHS Scotland services, include Health improvement, Efficiency, Access to treatment and Treatment | |
| The Commonwealth fund | US | A private framework that monitors performance across four domains: access, prevention and treatment, costs and potentially avoidable hospital use and health outcomes | |
| RAI-MDS | US / Canada | The assessment tool utilised in long term care homes and care at homes in Canada and the US | |
| NHPF | Australia | National Health Performance Framework (NHPF) provides a structure for reporting on health care quality and performance on a national level based on 40 health indicators grouped under 3 domains: health status, determinants of health and health system performance | |
| AACQA | Australia | New Aged Care Quality Standards will provide a new framework for quality surveyors to assess compliance and will apply to all aged care services from July 2018 | |

## Slide 13
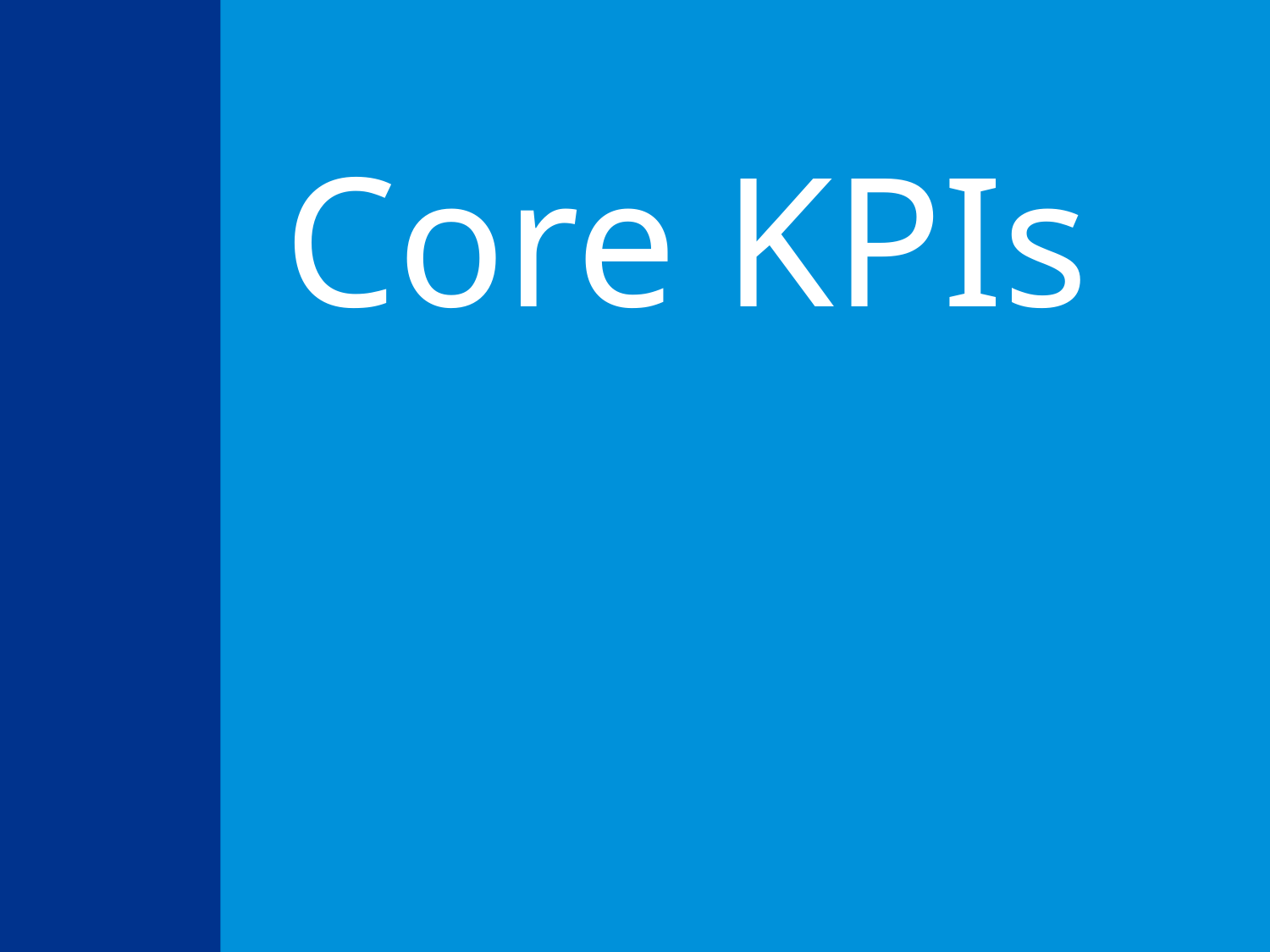

# Core KPIs

## Slide 14
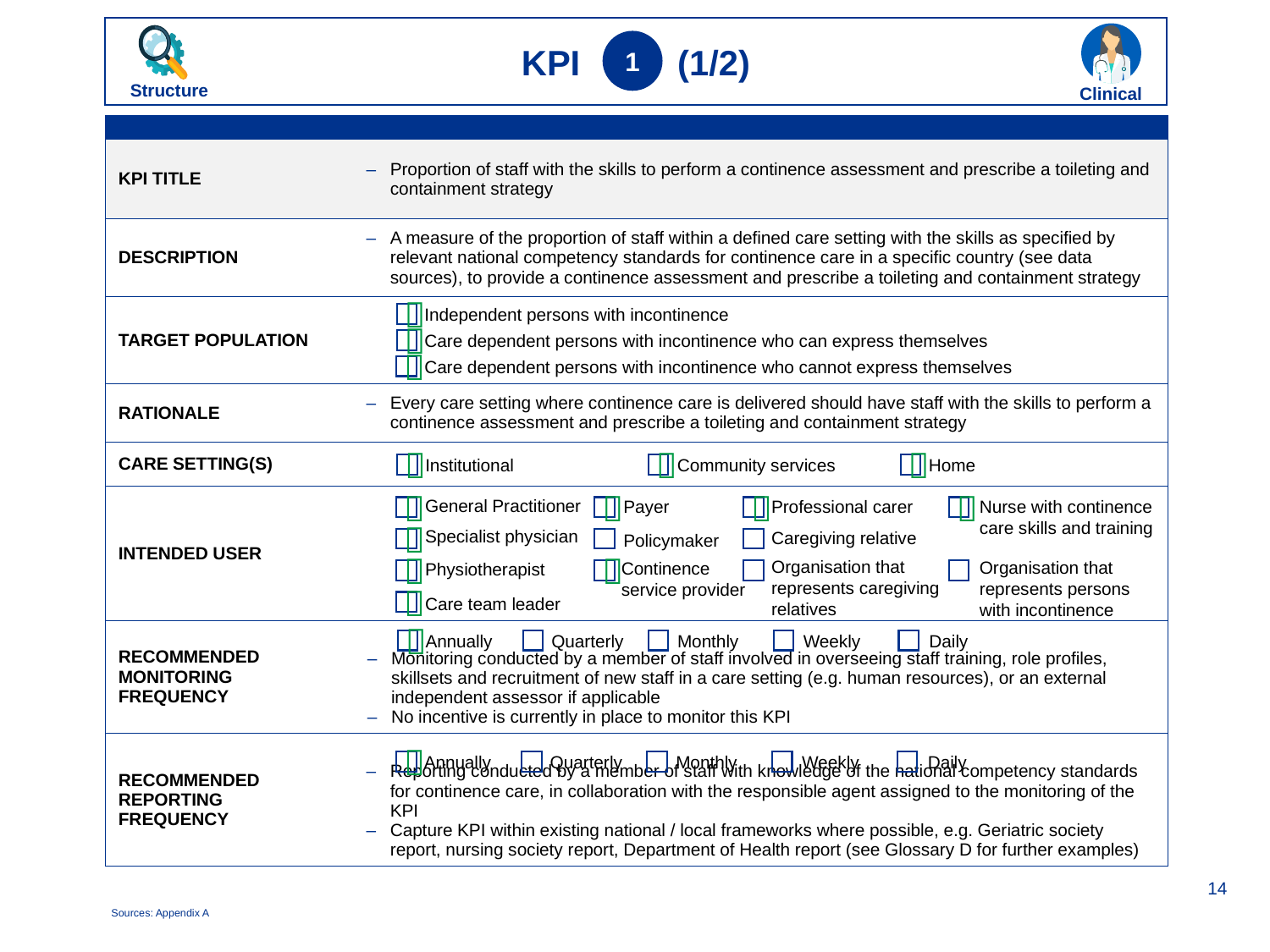

KPI (1/2)
1
Structure
Clinical
| | |
| --- | --- |
| KPI TITLE | Proportion of staff with the skills to perform a continence assessment and prescribe a toileting and containment strategy |
| DESCRIPTION | A measure of the proportion of staff within a defined care setting with the skills as specified by relevant national competency standards for continence care in a specific country (see data sources), to provide a continence assessment and prescribe a toileting and containment strategy |
| TARGET POPULATION | |
| RATIONALE | Every care setting where continence care is delivered should have staff with the skills to perform a continence assessment and prescribe a toileting and containment strategy |
| CARE SETTING(S) | |
| INTENDED USER | |
| RECOMMENDED MONITORING FREQUENCY | Monitoring conducted by a member of staff involved in overseeing staff training, role profiles, skillsets and recruitment of new staff in a care setting (e.g. human resources), or an external independent assessor if applicable No incentive is currently in place to monitor this KPI |
| RECOMMENDED REPORTING FREQUENCY | Reporting conducted by a member of staff with knowledge of the national competency standards for continence care, in collaboration with the responsible agent assigned to the monitoring of the KPI Capture KPI within existing national / local frameworks where possible, e.g. Geriatric society report, nursing society report, Department of Health report (see Glossary D for further examples) |
Independent persons with incontinence

Care dependent persons with incontinence who can express themselves

Care dependent persons with incontinence who cannot express themselves

Institutional
Community services
Home



General Practitioner
Professional carer
Payer
Nurse with continence care skills and training




Specialist physician
Caregiving relative
Policymaker

Physiotherapist


Continence service provider
Organisation that represents caregiving relatives
Organisation that represents persons with incontinence
Care team leader

Annually
Quarterly
Monthly
Weekly
Daily

Annually
Quarterly
Monthly
Weekly
Daily

Sources: Appendix A

## Slide 15
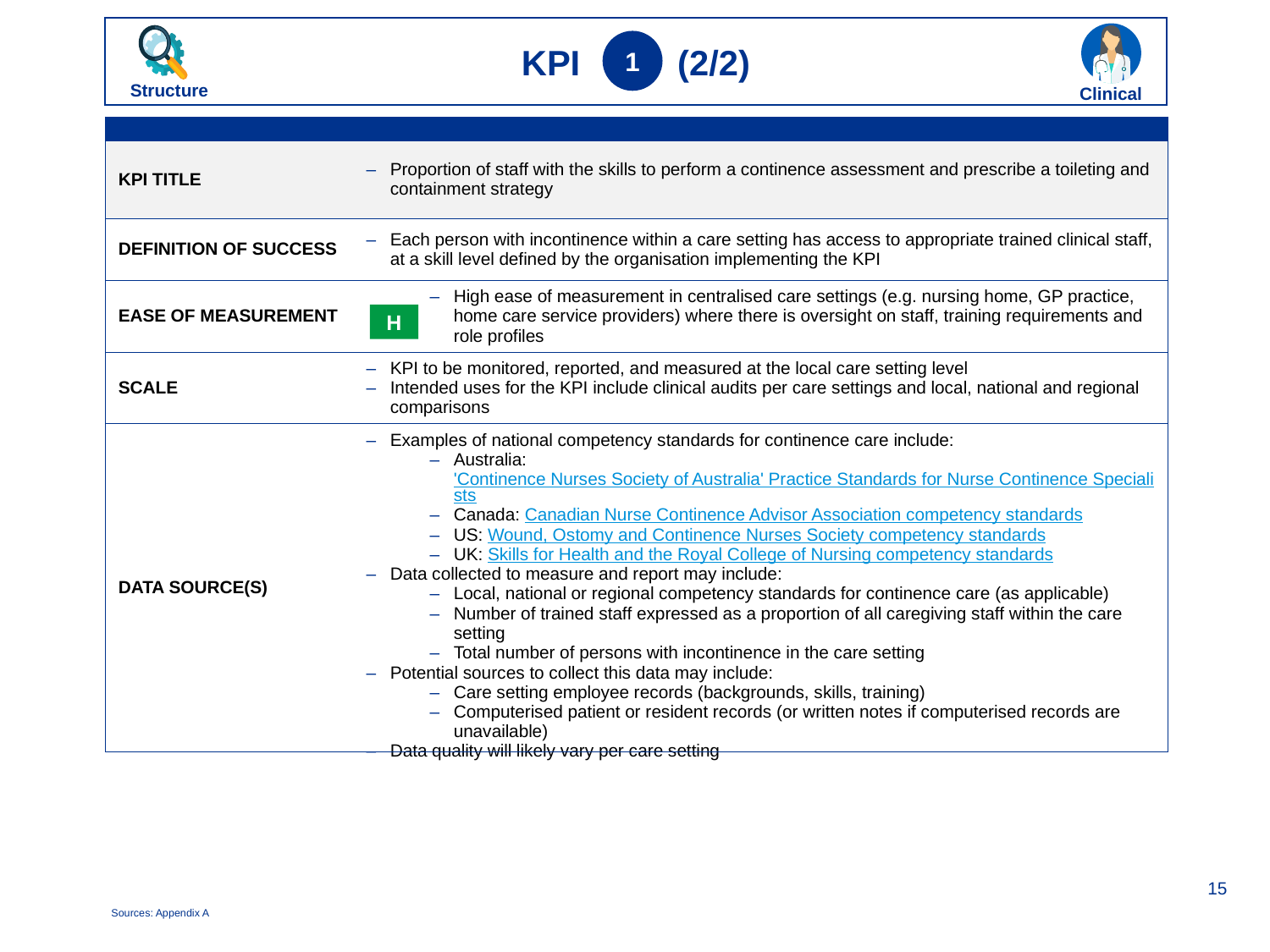

KPI (2/2)
1
Structure
Clinical
| | |
| --- | --- |
| KPI TITLE | Proportion of staff with the skills to perform a continence assessment and prescribe a toileting and containment strategy |
| DEFINITION OF SUCCESS | Each person with incontinence within a care setting has access to appropriate trained clinical staff, at a skill level defined by the organisation implementing the KPI |
| EASE OF MEASUREMENT | High ease of measurement in centralised care settings (e.g. nursing home, GP practice, home care service providers) where there is oversight on staff, training requirements and role profiles |
| SCALE | KPI to be monitored, reported, and measured at the local care setting level Intended uses for the KPI include clinical audits per care settings and local, national and regional comparisons |
| DATA SOURCE(S) | Examples of national competency standards for continence care include: Australia: 'Continence Nurses Society of Australia' Practice Standards for Nurse Continence Specialists Canada: Canadian Nurse Continence Advisor Association competency standards US: Wound, Ostomy and Continence Nurses Society competency standards UK: Skills for Health and the Royal College of Nursing competency standards Data collected to measure and report may include: Local, national or regional competency standards for continence care (as applicable) Number of trained staff expressed as a proportion of all caregiving staff within the care setting Total number of persons with incontinence in the care setting Potential sources to collect this data may include: Care setting employee records (backgrounds, skills, training) Computerised patient or resident records (or written notes if computerised records are unavailable) Data quality will likely vary per care setting |
H
Sources: Appendix A

## Slide 16
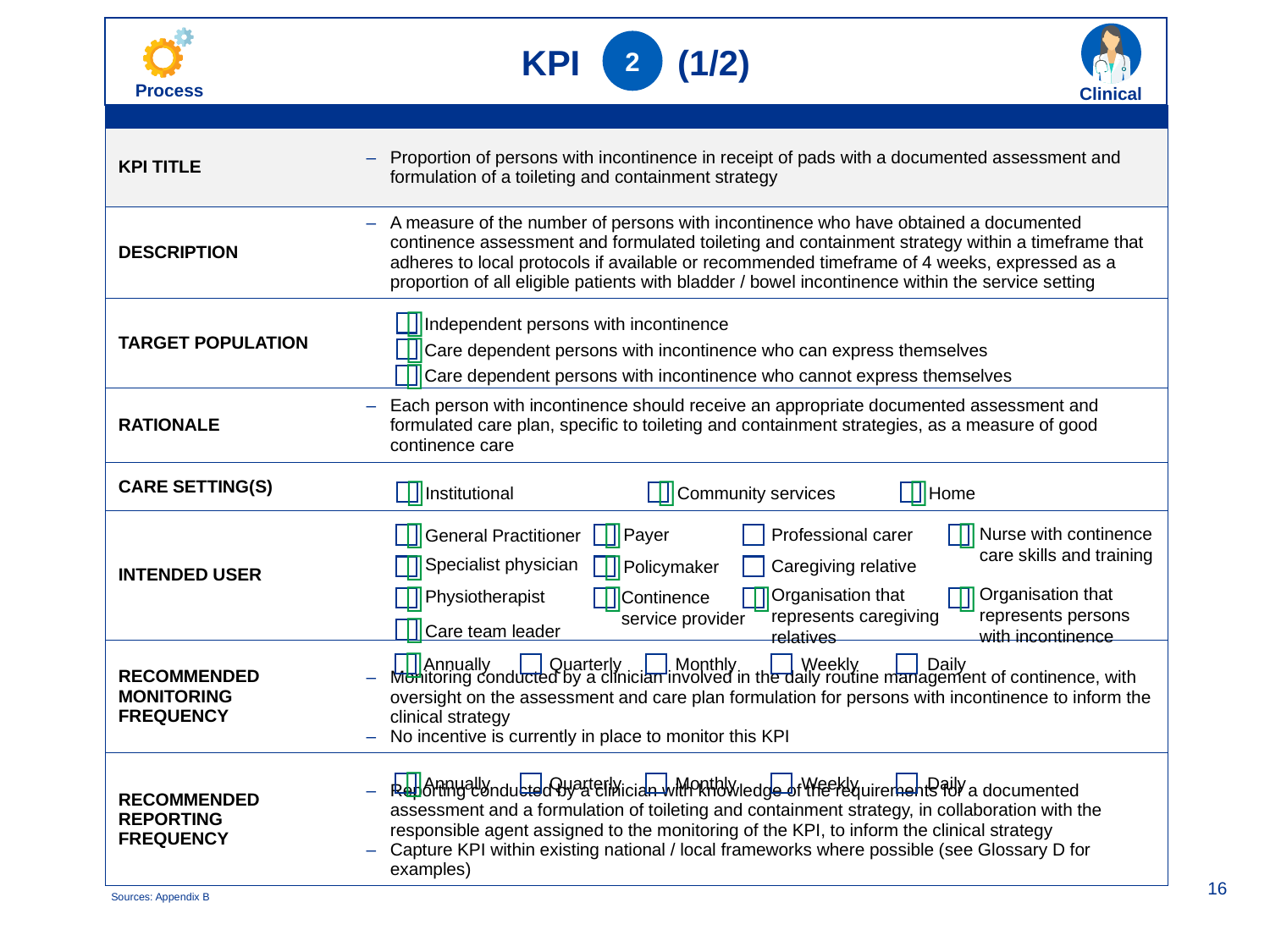

KPI (1/2)
2
Process
Clinical
| | |
| --- | --- |
| KPI TITLE | Proportion of persons with incontinence in receipt of pads with a documented assessment and formulation of a toileting and containment strategy |
| DESCRIPTION | A measure of the number of persons with incontinence who have obtained a documented continence assessment and formulated toileting and containment strategy within a timeframe that adheres to local protocols if available or recommended timeframe of 4 weeks, expressed as a proportion of all eligible patients with bladder / bowel incontinence within the service setting |
| TARGET POPULATION | |
| RATIONALE | Each person with incontinence should receive an appropriate documented assessment and formulated care plan, specific to toileting and containment strategies, as a measure of good continence care |
| CARE SETTING(S) | |
| INTENDED USER | |
| RECOMMENDED MONITORING FREQUENCY | Monitoring conducted by a clinician involved in the daily routine management of continence, with oversight on the assessment and care plan formulation for persons with incontinence to inform the clinical strategy No incentive is currently in place to monitor this KPI |
| RECOMMENDED REPORTING FREQUENCY | Reporting conducted by a clinician with knowledge of the requirements for a documented assessment and a formulation of toileting and containment strategy, in collaboration with the responsible agent assigned to the monitoring of the KPI, to inform the clinical strategy Capture KPI within existing national / local frameworks where possible (see Glossary D for examples) |
Independent persons with incontinence

Care dependent persons with incontinence who can express themselves

Care dependent persons with incontinence who cannot express themselves

Institutional
Community services
Home



General Practitioner
Professional carer
Payer
Nurse with continence care skills and training



Specialist physician
Caregiving relative
Policymaker


Physiotherapist




Continence service provider
Organisation that represents caregiving relatives
Organisation that represents persons with incontinence
Care team leader

Annually
Quarterly
Monthly
Weekly
Daily

Annually
Quarterly
Monthly
Weekly
Daily

Sources: Appendix B

## Slide 17
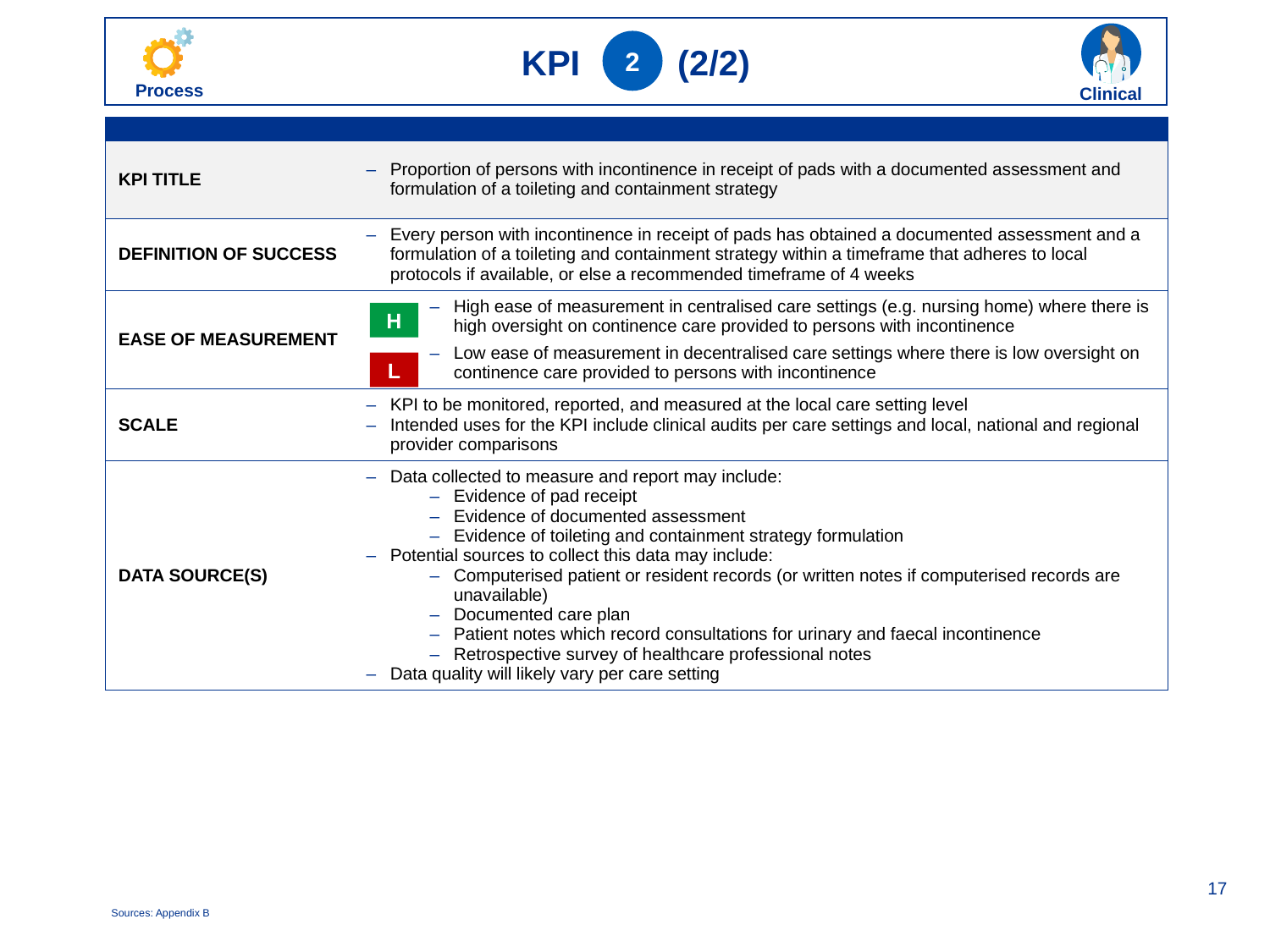

KPI (2/2)
2
Process
Clinical
| | |
| --- | --- |
| KPI TITLE | Proportion of persons with incontinence in receipt of pads with a documented assessment and formulation of a toileting and containment strategy |
| DEFINITION OF SUCCESS | Every person with incontinence in receipt of pads has obtained a documented assessment and a formulation of a toileting and containment strategy within a timeframe that adheres to local protocols if available, or else a recommended timeframe of 4 weeks |
| EASE OF MEASUREMENT | High ease of measurement in centralised care settings (e.g. nursing home) where there is high oversight on continence care provided to persons with incontinence Low ease of measurement in decentralised care settings where there is low oversight on continence care provided to persons with incontinence |
| SCALE | KPI to be monitored, reported, and measured at the local care setting level Intended uses for the KPI include clinical audits per care settings and local, national and regional provider comparisons |
| DATA SOURCE(S) | Data collected to measure and report may include: Evidence of pad receipt Evidence of documented assessment Evidence of toileting and containment strategy formulation Potential sources to collect this data may include: Computerised patient or resident records (or written notes if computerised records are unavailable) Documented care plan Patient notes which record consultations for urinary and faecal incontinence Retrospective survey of healthcare professional notes Data quality will likely vary per care setting |
H
L
Sources: Appendix B

## Slide 18
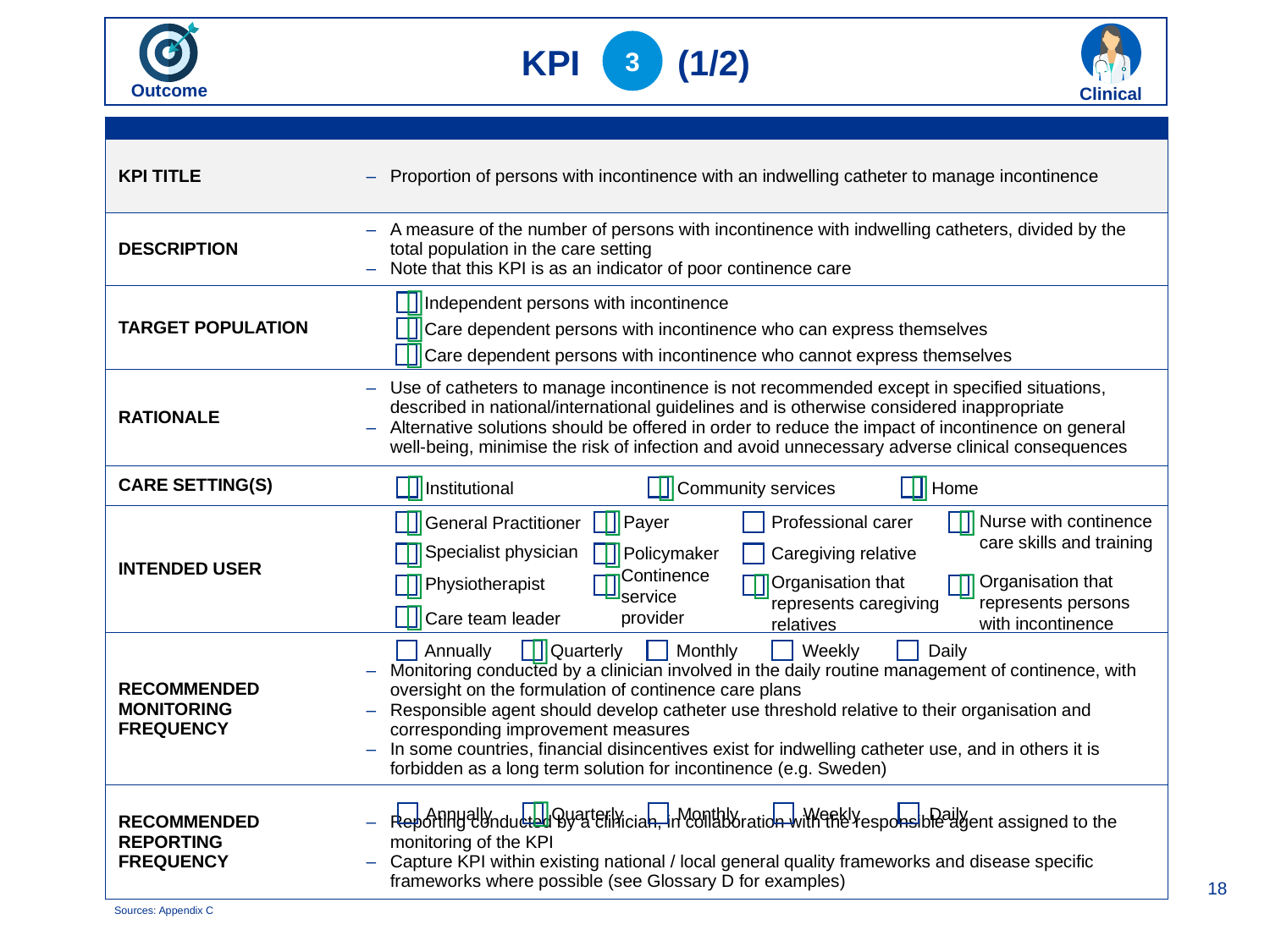

KPI (1/2)
3
Outcome
Clinical
| | |
| --- | --- |
| KPI TITLE | Proportion of persons with incontinence with an indwelling catheter to manage incontinence |
| DESCRIPTION | A measure of the number of persons with incontinence with indwelling catheters, divided by the total population in the care setting Note that this KPI is as an indicator of poor continence care |
| TARGET POPULATION | |
| RATIONALE | Use of catheters to manage incontinence is not recommended except in specified situations, described in national/international guidelines and is otherwise considered inappropriate Alternative solutions should be offered in order to reduce the impact of incontinence on general well-being, minimise the risk of infection and avoid unnecessary adverse clinical consequences |
| CARE SETTING(S) | |
| INTENDED USER | |
| RECOMMENDED MONITORING FREQUENCY | Monitoring conducted by a clinician involved in the daily routine management of continence, with oversight on the formulation of continence care plans Responsible agent should develop catheter use threshold relative to their organisation and corresponding improvement measures In some countries, financial disincentives exist for indwelling catheter use, and in others it is forbidden as a long term solution for incontinence (e.g. Sweden) |
| RECOMMENDED REPORTING FREQUENCY | Reporting conducted by a clinician, in collaboration with the responsible agent assigned to the monitoring of the KPI Capture KPI within existing national / local general quality frameworks and disease specific frameworks where possible (see Glossary D for examples) |
Independent persons with incontinence

Care dependent persons with incontinence who can express themselves

Care dependent persons with incontinence who cannot express themselves

Institutional
Community services
Home



General Practitioner
Professional carer
Payer
Nurse with continence care skills and training



Specialist physician
Caregiving relative
Policymaker


Physiotherapist




Continence service provider
Organisation that represents caregiving relatives
Organisation that represents persons with incontinence
Care team leader

Annually
Quarterly
Monthly
Weekly
Daily

Annually
Quarterly
Monthly
Weekly
Daily

Sources: Appendix C

## Slide 19
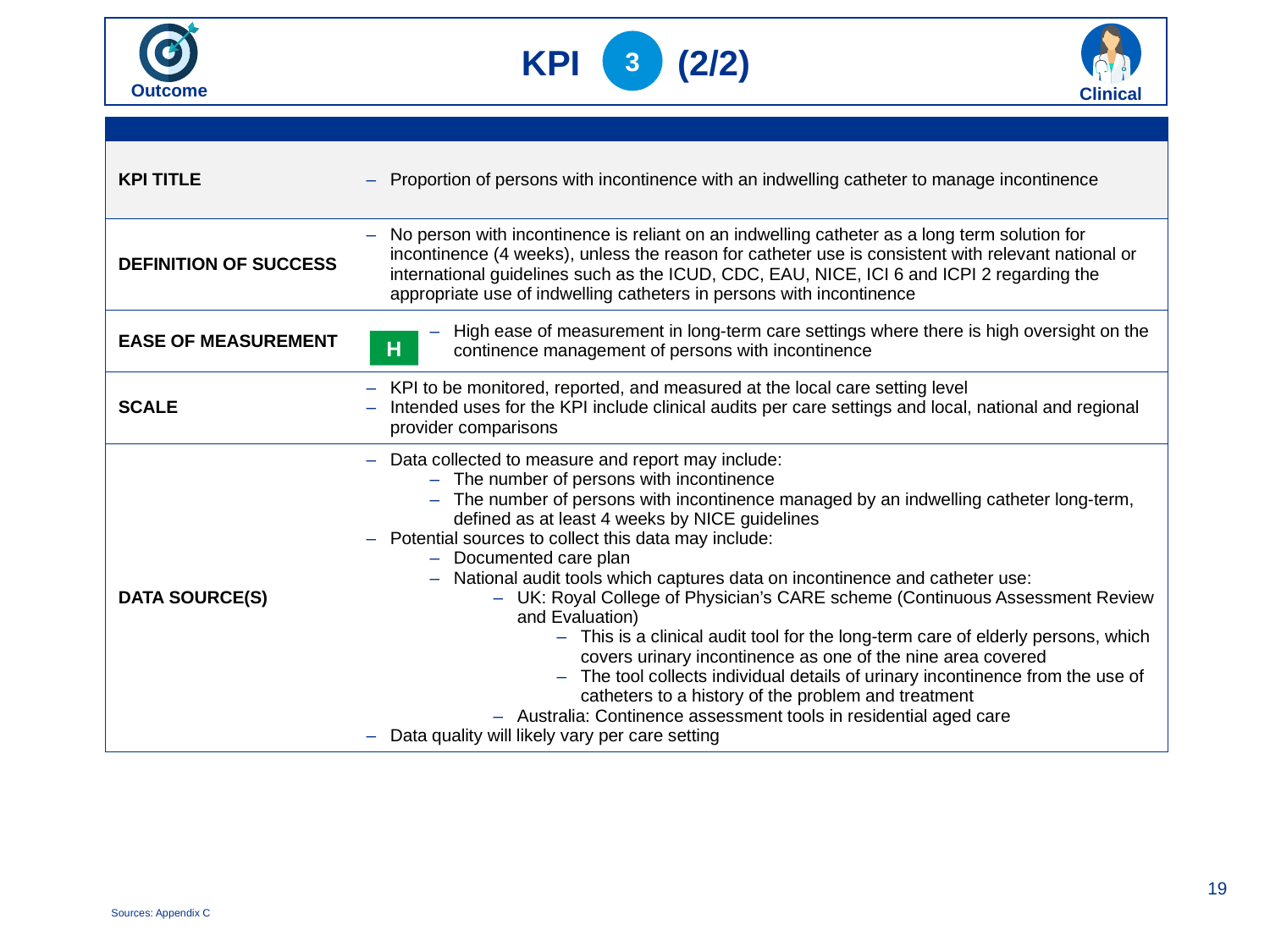

KPI (2/2)
3
Outcome
Clinical
| | |
| --- | --- |
| KPI TITLE | Proportion of persons with incontinence with an indwelling catheter to manage incontinence |
| DEFINITION OF SUCCESS | No person with incontinence is reliant on an indwelling catheter as a long term solution for incontinence (4 weeks), unless the reason for catheter use is consistent with relevant national or international guidelines such as the ICUD, CDC, EAU, NICE, ICI 6 and ICPI 2 regarding the appropriate use of indwelling catheters in persons with incontinence |
| EASE OF MEASUREMENT | High ease of measurement in long-term care settings where there is high oversight on the continence management of persons with incontinence |
| SCALE | KPI to be monitored, reported, and measured at the local care setting level Intended uses for the KPI include clinical audits per care settings and local, national and regional provider comparisons |
| DATA SOURCE(S) | Data collected to measure and report may include: The number of persons with incontinence The number of persons with incontinence managed by an indwelling catheter long-term, defined as at least 4 weeks by NICE guidelines Potential sources to collect this data may include: Documented care plan National audit tools which captures data on incontinence and catheter use: UK: Royal College of Physician’s CARE scheme (Continuous Assessment Review and Evaluation) This is a clinical audit tool for the long-term care of elderly persons, which covers urinary incontinence as one of the nine area covered The tool collects individual details of urinary incontinence from the use of catheters to a history of the problem and treatment Australia: Continence assessment tools in residential aged care Data quality will likely vary per care setting |
H
Sources: Appendix C

## Slide 20
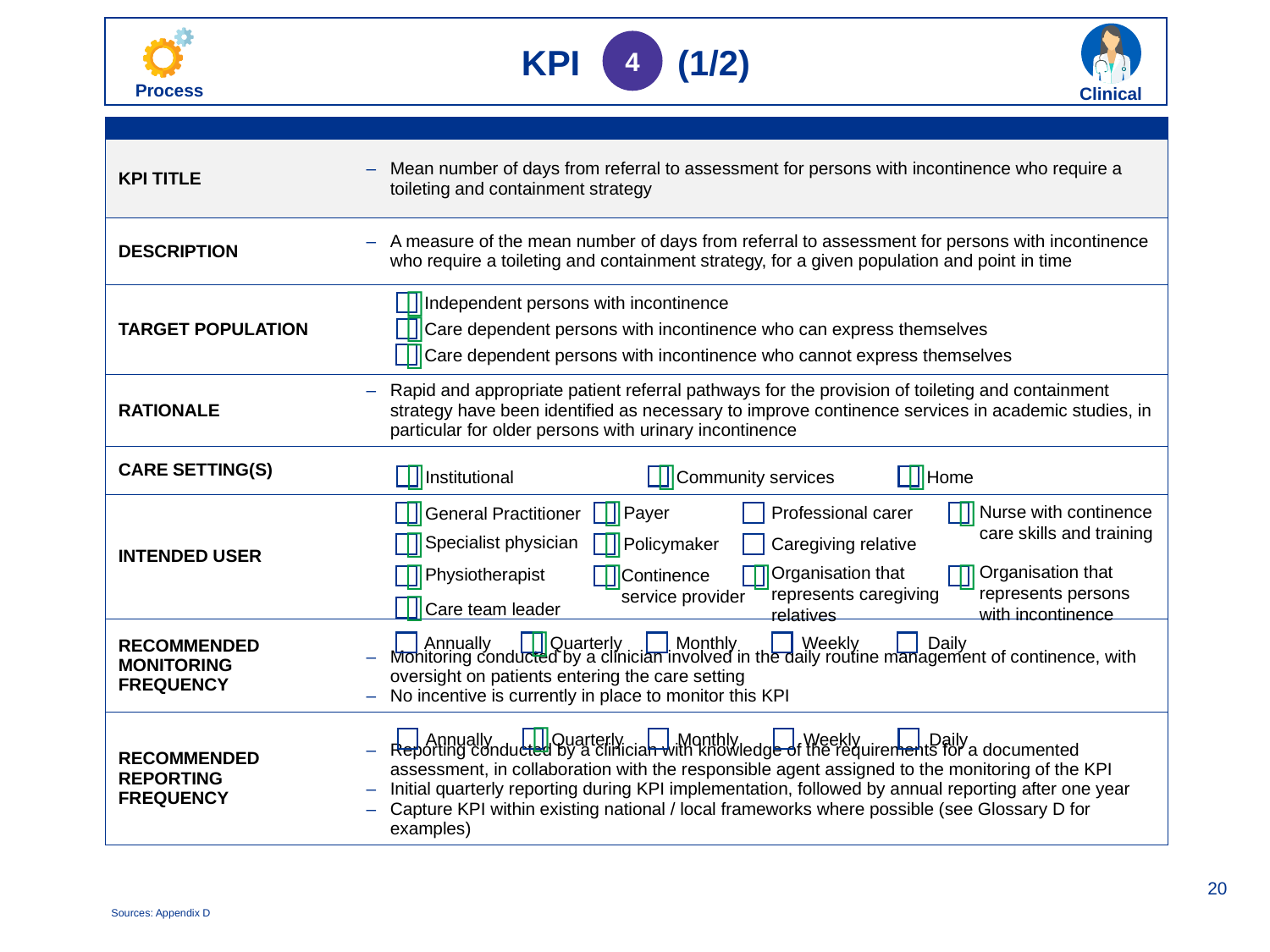

KPI (1/2)
4
Process
Clinical
| | |
| --- | --- |
| KPI TITLE | Mean number of days from referral to assessment for persons with incontinence who require a toileting and containment strategy |
| DESCRIPTION | A measure of the mean number of days from referral to assessment for persons with incontinence who require a toileting and containment strategy, for a given population and point in time |
| TARGET POPULATION | |
| RATIONALE | Rapid and appropriate patient referral pathways for the provision of toileting and containment strategy have been identified as necessary to improve continence services in academic studies, in particular for older persons with urinary incontinence |
| CARE SETTING(S) | |
| INTENDED USER | |
| RECOMMENDED MONITORING FREQUENCY | Monitoring conducted by a clinician involved in the daily routine management of continence, with oversight on patients entering the care setting No incentive is currently in place to monitor this KPI |
| RECOMMENDED REPORTING FREQUENCY | Reporting conducted by a clinician with knowledge of the requirements for a documented assessment, in collaboration with the responsible agent assigned to the monitoring of the KPI Initial quarterly reporting during KPI implementation, followed by annual reporting after one year Capture KPI within existing national / local frameworks where possible (see Glossary D for examples) |
Independent persons with incontinence

Care dependent persons with incontinence who can express themselves

Care dependent persons with incontinence who cannot express themselves

Institutional
Community services
Home



General Practitioner
Professional carer
Payer
Nurse with continence care skills and training



Specialist physician
Caregiving relative
Policymaker


Physiotherapist




Continence service provider
Organisation that represents caregiving relatives
Organisation that represents persons with incontinence
Care team leader

Annually
Quarterly
Monthly
Weekly
Daily

Annually
Quarterly
Monthly
Weekly
Daily

Sources: Appendix D

## Slide 21
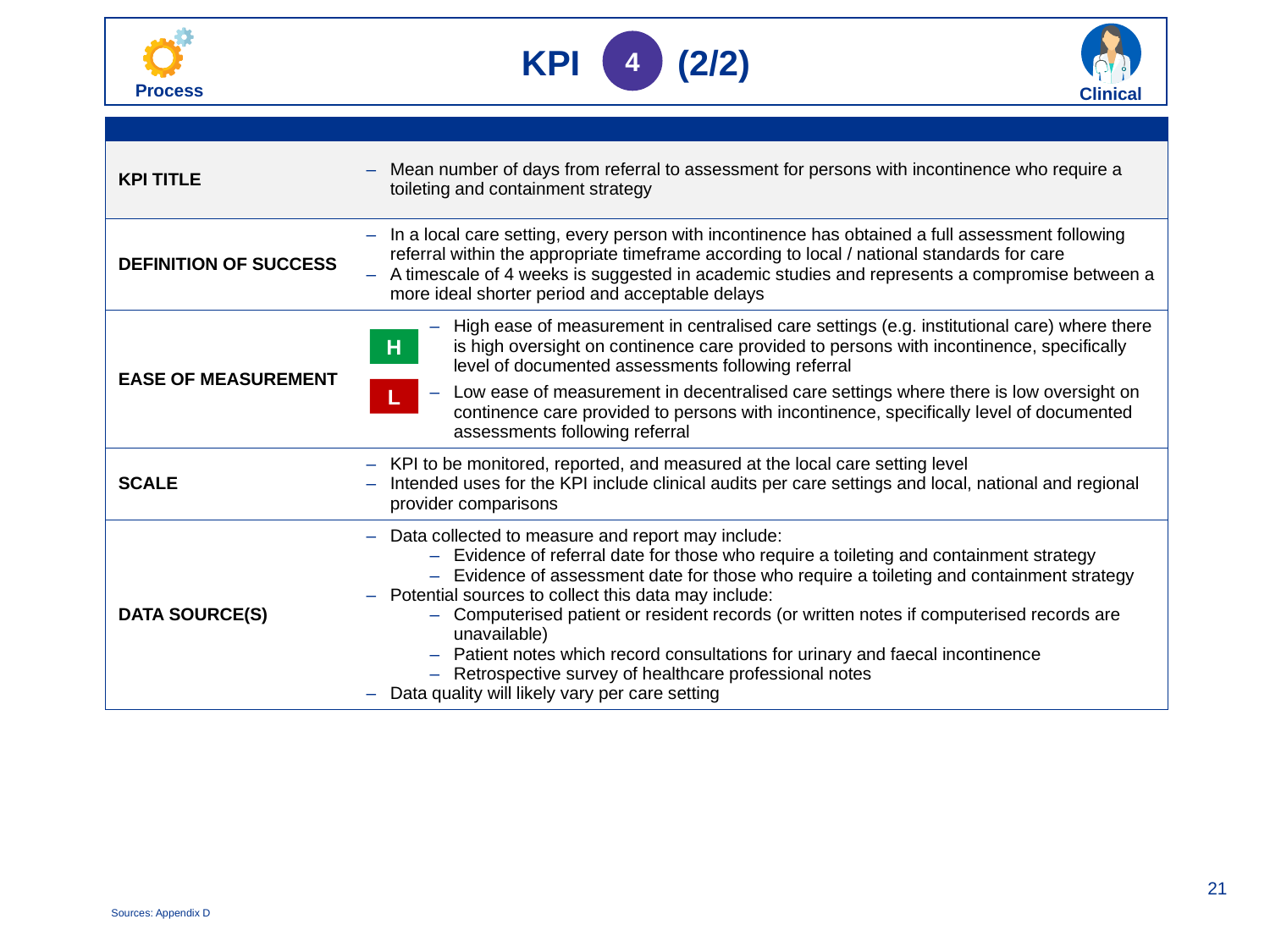

KPI (2/2)
4
Process
Clinical
| | |
| --- | --- |
| KPI TITLE | Mean number of days from referral to assessment for persons with incontinence who require a toileting and containment strategy |
| DEFINITION OF SUCCESS | In a local care setting, every person with incontinence has obtained a full assessment following referral within the appropriate timeframe according to local / national standards for care A timescale of 4 weeks is suggested in academic studies and represents a compromise between a more ideal shorter period and acceptable delays |
| EASE OF MEASUREMENT | High ease of measurement in centralised care settings (e.g. institutional care) where there is high oversight on continence care provided to persons with incontinence, specifically level of documented assessments following referral Low ease of measurement in decentralised care settings where there is low oversight on continence care provided to persons with incontinence, specifically level of documented assessments following referral |
| SCALE | KPI to be monitored, reported, and measured at the local care setting level Intended uses for the KPI include clinical audits per care settings and local, national and regional provider comparisons |
| DATA SOURCE(S) | Data collected to measure and report may include: Evidence of referral date for those who require a toileting and containment strategy Evidence of assessment date for those who require a toileting and containment strategy Potential sources to collect this data may include: Computerised patient or resident records (or written notes if computerised records are unavailable) Patient notes which record consultations for urinary and faecal incontinence Retrospective survey of healthcare professional notes Data quality will likely vary per care setting |
H
L
Sources: Appendix D

## Slide 22
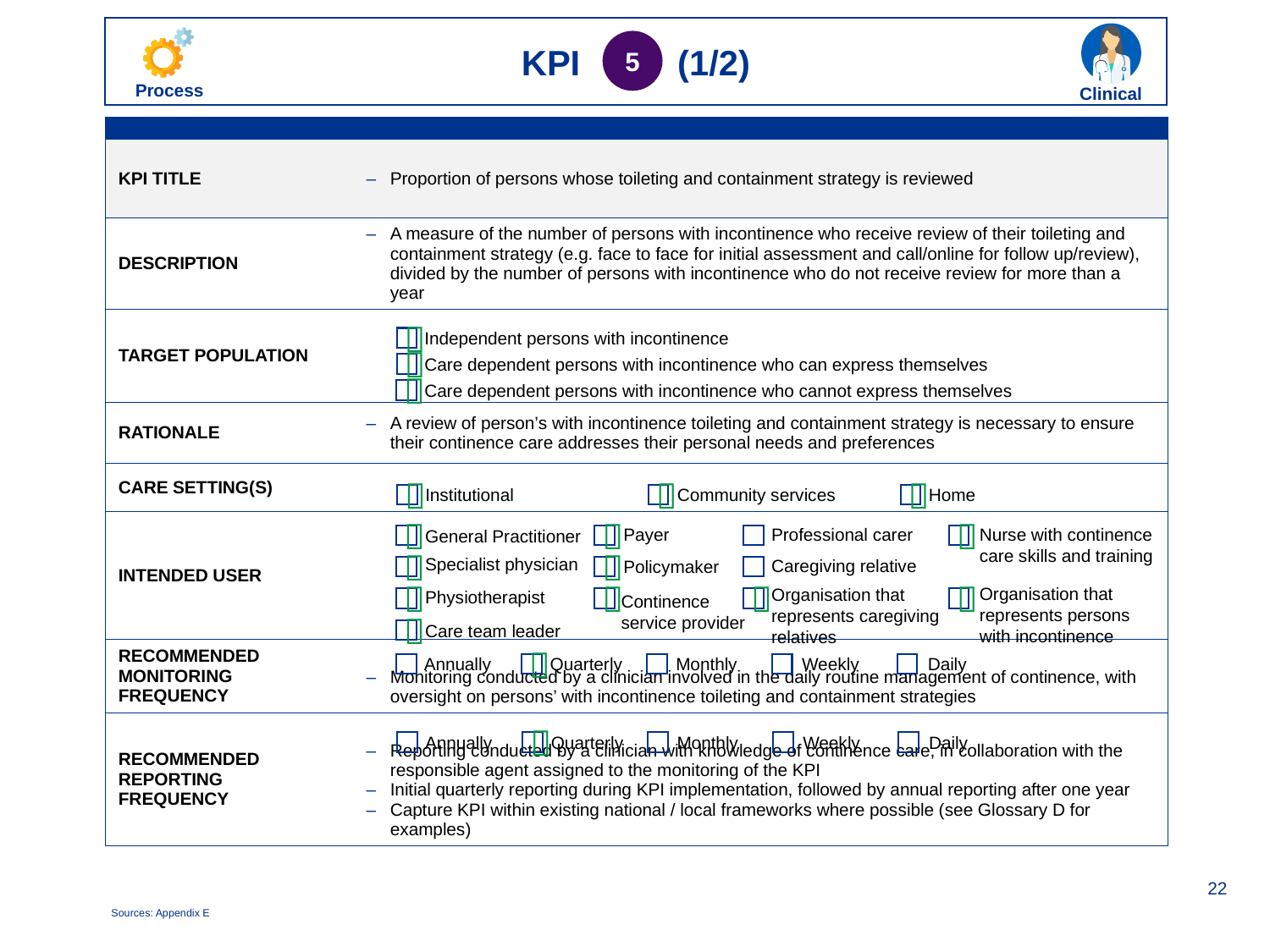

KPI (1/2)
5
Process
Clinical
| | |
| --- | --- |
| KPI TITLE | Proportion of persons whose toileting and containment strategy is reviewed |
| DESCRIPTION | A measure of the number of persons with incontinence who receive review of their toileting and containment strategy (e.g. face to face for initial assessment and call/online for follow up/review), divided by the number of persons with incontinence who do not receive review for more than a year |
| TARGET POPULATION | |
| RATIONALE | A review of person’s with incontinence toileting and containment strategy is necessary to ensure their continence care addresses their personal needs and preferences |
| CARE SETTING(S) | |
| INTENDED USER | |
| RECOMMENDED MONITORING FREQUENCY | Monitoring conducted by a clinician involved in the daily routine management of continence, with oversight on persons’ with incontinence toileting and containment strategies |
| RECOMMENDED REPORTING FREQUENCY | Reporting conducted by a clinician with knowledge of continence care, in collaboration with the responsible agent assigned to the monitoring of the KPI Initial quarterly reporting during KPI implementation, followed by annual reporting after one year Capture KPI within existing national / local frameworks where possible (see Glossary D for examples) |
Independent persons with incontinence

Care dependent persons with incontinence who can express themselves

Care dependent persons with incontinence who cannot express themselves

Institutional
Community services
Home



General Practitioner
Professional carer
Payer
Nurse with continence care skills and training



Specialist physician
Caregiving relative
Policymaker


Physiotherapist




Continence service provider
Organisation that represents caregiving relatives
Organisation that represents persons with incontinence
Care team leader

Annually
Quarterly
Monthly
Weekly
Daily

Annually
Quarterly
Monthly
Weekly
Daily

Sources: Appendix E

## Slide 23
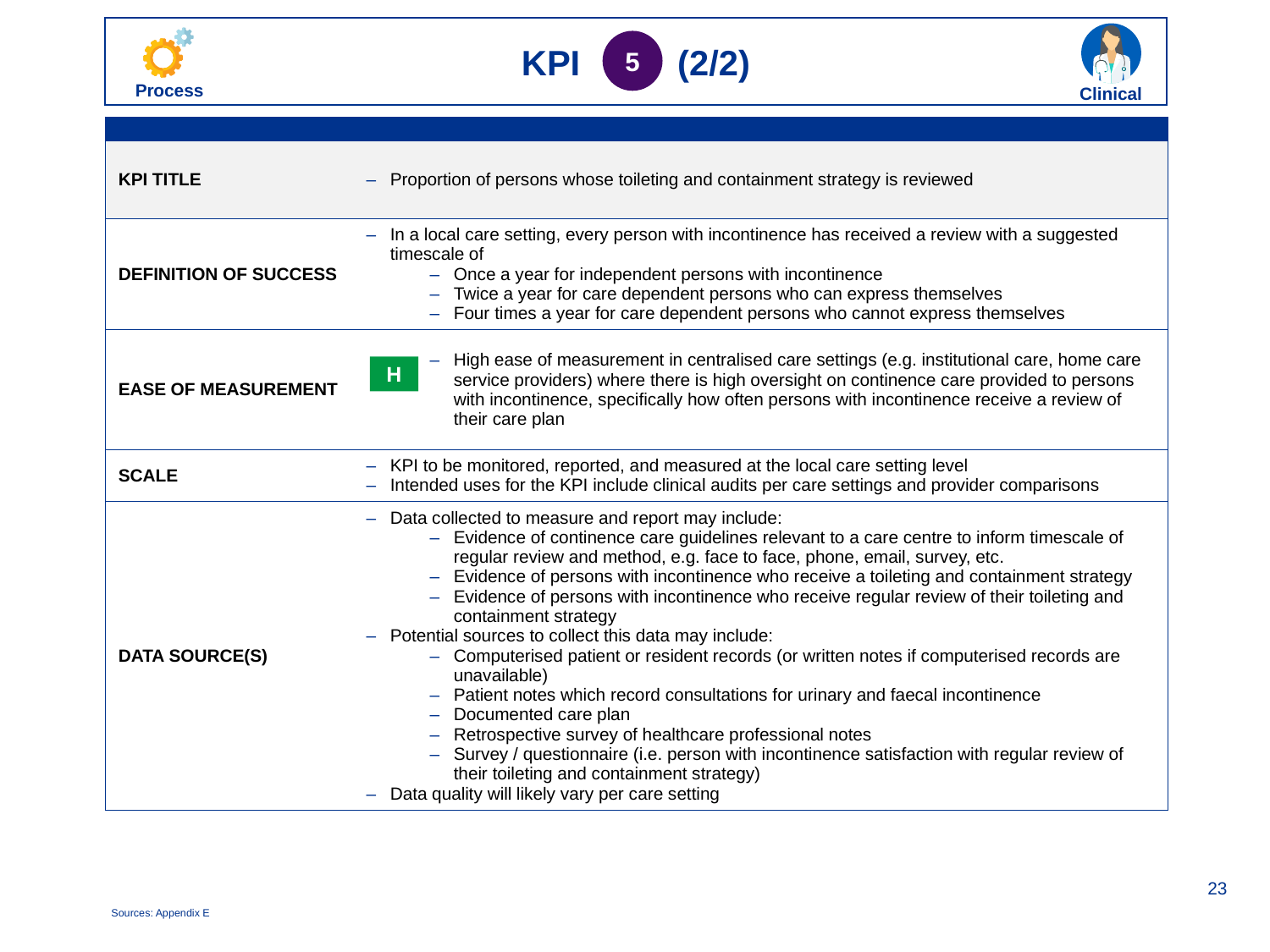

KPI (2/2)
5
Process
Clinical
| | |
| --- | --- |
| KPI TITLE | Proportion of persons whose toileting and containment strategy is reviewed |
| DEFINITION OF SUCCESS | In a local care setting, every person with incontinence has received a review with a suggested timescale of Once a year for independent persons with incontinence Twice a year for care dependent persons who can express themselves Four times a year for care dependent persons who cannot express themselves |
| EASE OF MEASUREMENT | High ease of measurement in centralised care settings (e.g. institutional care, home care service providers) where there is high oversight on continence care provided to persons with incontinence, specifically how often persons with incontinence receive a review of their care plan |
| SCALE | KPI to be monitored, reported, and measured at the local care setting level Intended uses for the KPI include clinical audits per care settings and provider comparisons |
| DATA SOURCE(S) | Data collected to measure and report may include: Evidence of continence care guidelines relevant to a care centre to inform timescale of regular review and method, e.g. face to face, phone, email, survey, etc. Evidence of persons with incontinence who receive a toileting and containment strategy Evidence of persons with incontinence who receive regular review of their toileting and containment strategy Potential sources to collect this data may include: Computerised patient or resident records (or written notes if computerised records are unavailable) Patient notes which record consultations for urinary and faecal incontinence Documented care plan Retrospective survey of healthcare professional notes Survey / questionnaire (i.e. person with incontinence satisfaction with regular review of their toileting and containment strategy) Data quality will likely vary per care setting |
H
Sources: Appendix E

## Slide 24
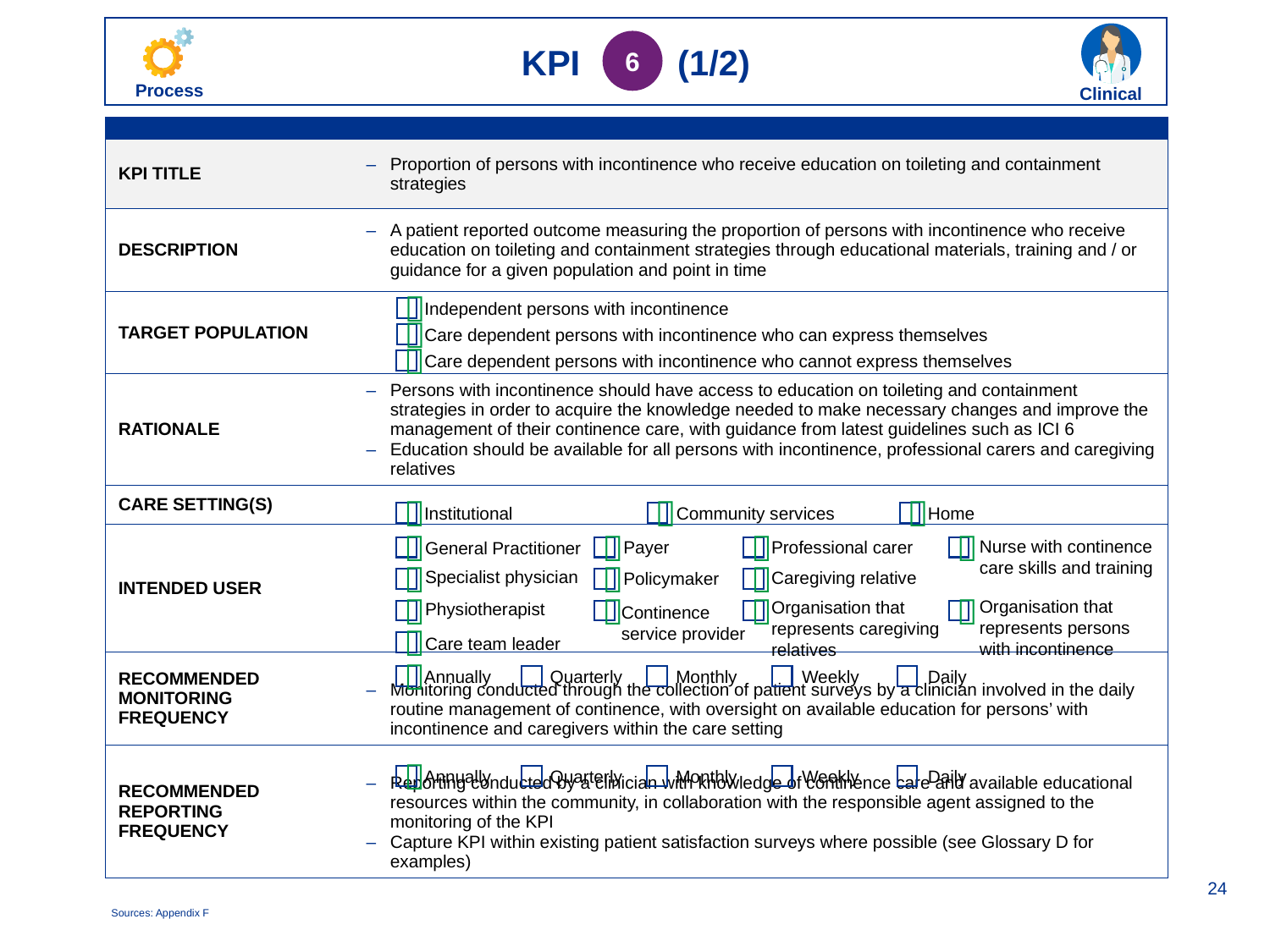

KPI (1/2)
6
Process
Clinical
| | |
| --- | --- |
| KPI TITLE | Proportion of persons with incontinence who receive education on toileting and containment strategies |
| DESCRIPTION | A patient reported outcome measuring the proportion of persons with incontinence who receive education on toileting and containment strategies through educational materials, training and / or guidance for a given population and point in time |
| TARGET POPULATION | |
| RATIONALE | Persons with incontinence should have access to education on toileting and containment strategies in order to acquire the knowledge needed to make necessary changes and improve the management of their continence care, with guidance from latest guidelines such as ICI 6 Education should be available for all persons with incontinence, professional carers and caregiving relatives |
| CARE SETTING(S) | |
| INTENDED USER | |
| RECOMMENDED MONITORING FREQUENCY | Monitoring conducted through the collection of patient surveys by a clinician involved in the daily routine management of continence, with oversight on available education for persons’ with incontinence and caregivers within the care setting |
| RECOMMENDED REPORTING FREQUENCY | Reporting conducted by a clinician with knowledge of continence care and available educational resources within the community, in collaboration with the responsible agent assigned to the monitoring of the KPI Capture KPI within existing patient satisfaction surveys where possible (see Glossary D for examples) |
Independent persons with incontinence

Care dependent persons with incontinence who can express themselves

Care dependent persons with incontinence who cannot express themselves

Institutional
Community services
Home



General Practitioner
Professional carer
Payer
Nurse with continence care skills and training




Specialist physician
Caregiving relative
Policymaker



Physiotherapist




Continence service provider
Organisation that represents caregiving relatives
Organisation that represents persons with incontinence
Care team leader

Annually
Quarterly
Monthly
Weekly
Daily

Annually
Quarterly
Monthly
Weekly
Daily

Sources: Appendix F

## Slide 25
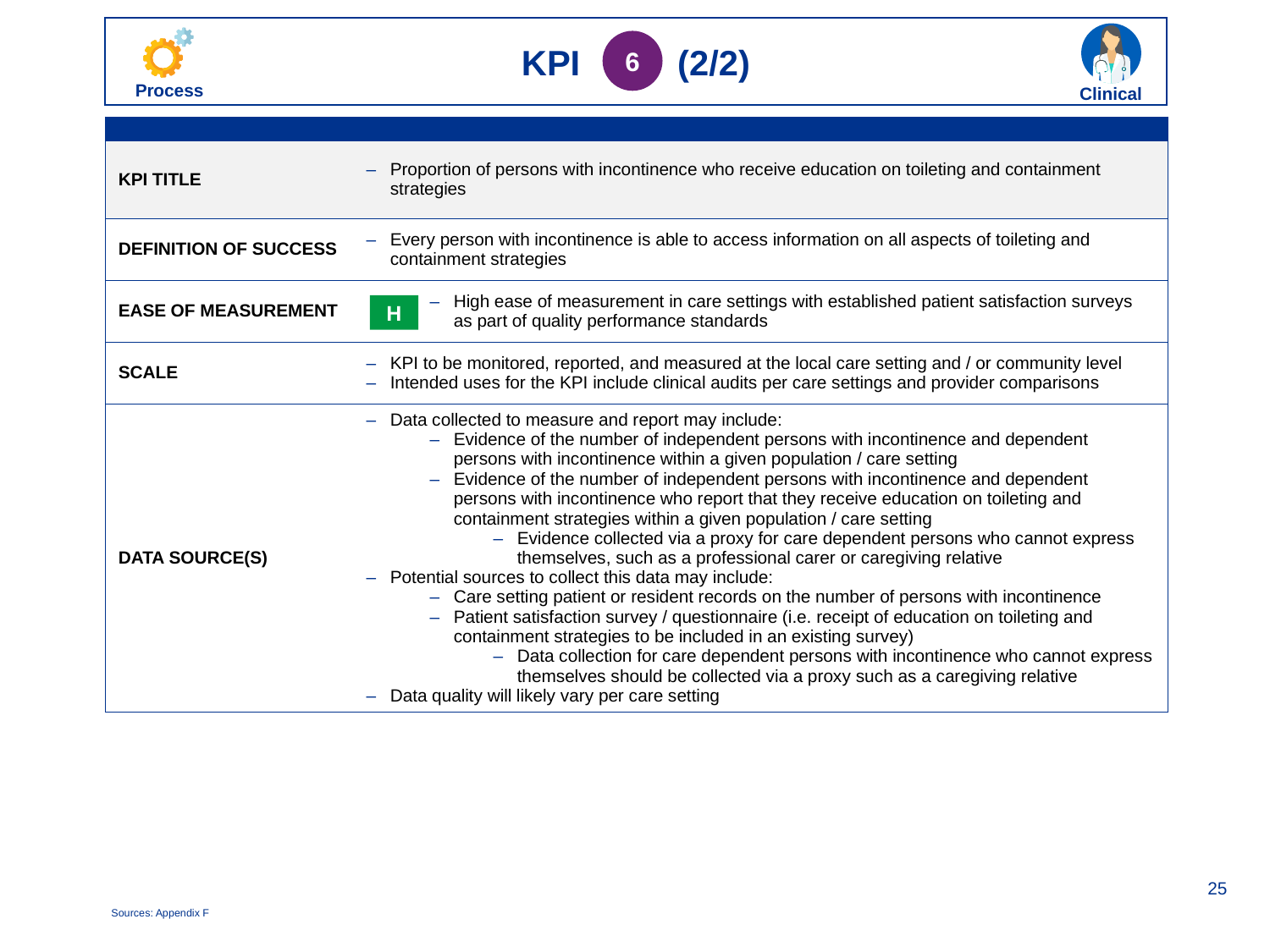

KPI (2/2)
6
Process
Clinical
| | |
| --- | --- |
| KPI TITLE | Proportion of persons with incontinence who receive education on toileting and containment strategies |
| DEFINITION OF SUCCESS | Every person with incontinence is able to access information on all aspects of toileting and containment strategies |
| EASE OF MEASUREMENT | High ease of measurement in care settings with established patient satisfaction surveys as part of quality performance standards |
| SCALE | KPI to be monitored, reported, and measured at the local care setting and / or community level Intended uses for the KPI include clinical audits per care settings and provider comparisons |
| DATA SOURCE(S) | Data collected to measure and report may include: Evidence of the number of independent persons with incontinence and dependent persons with incontinence within a given population / care setting Evidence of the number of independent persons with incontinence and dependent persons with incontinence who report that they receive education on toileting and containment strategies within a given population / care setting Evidence collected via a proxy for care dependent persons who cannot express themselves, such as a professional carer or caregiving relative Potential sources to collect this data may include: Care setting patient or resident records on the number of persons with incontinence Patient satisfaction survey / questionnaire (i.e. receipt of education on toileting and containment strategies to be included in an existing survey) Data collection for care dependent persons with incontinence who cannot express themselves should be collected via a proxy such as a caregiving relative Data quality will likely vary per care setting |
H
Sources: Appendix F

## Slide 26
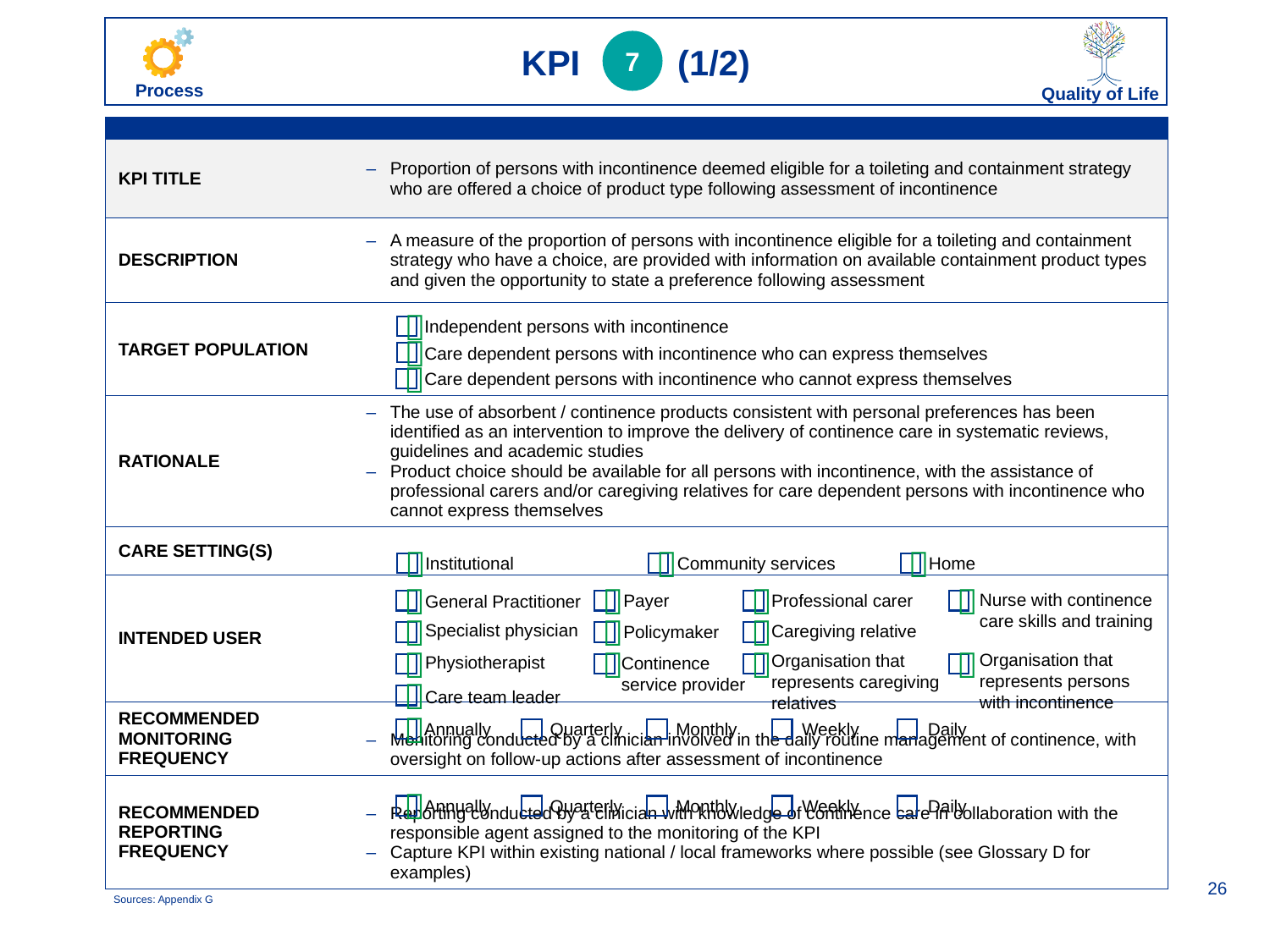

KPI (1/2)
7
Process
Quality of Life
| | |
| --- | --- |
| KPI TITLE | Proportion of persons with incontinence deemed eligible for a toileting and containment strategy who are offered a choice of product type following assessment of incontinence |
| DESCRIPTION | A measure of the proportion of persons with incontinence eligible for a toileting and containment strategy who have a choice, are provided with information on available containment product types and given the opportunity to state a preference following assessment |
| TARGET POPULATION | |
| RATIONALE | The use of absorbent / continence products consistent with personal preferences has been identified as an intervention to improve the delivery of continence care in systematic reviews, guidelines and academic studies Product choice should be available for all persons with incontinence, with the assistance of professional carers and/or caregiving relatives for care dependent persons with incontinence who cannot express themselves |
| CARE SETTING(S) | |
| INTENDED USER | |
| RECOMMENDED MONITORING FREQUENCY | Monitoring conducted by a clinician involved in the daily routine management of continence, with oversight on follow-up actions after assessment of incontinence |
| RECOMMENDED REPORTING FREQUENCY | Reporting conducted by a clinician with knowledge of continence care in collaboration with the responsible agent assigned to the monitoring of the KPI Capture KPI within existing national / local frameworks where possible (see Glossary D for examples) |
Independent persons with incontinence

Care dependent persons with incontinence who can express themselves

Care dependent persons with incontinence who cannot express themselves

Institutional
Community services
Home



General Practitioner
Professional carer
Payer
Nurse with continence care skills and training




Specialist physician
Caregiving relative
Policymaker



Physiotherapist




Continence service provider
Organisation that represents caregiving relatives
Organisation that represents persons with incontinence
Care team leader

Annually
Quarterly
Monthly
Weekly
Daily

Annually
Quarterly
Monthly
Weekly
Daily

Sources: Appendix G

## Slide 27
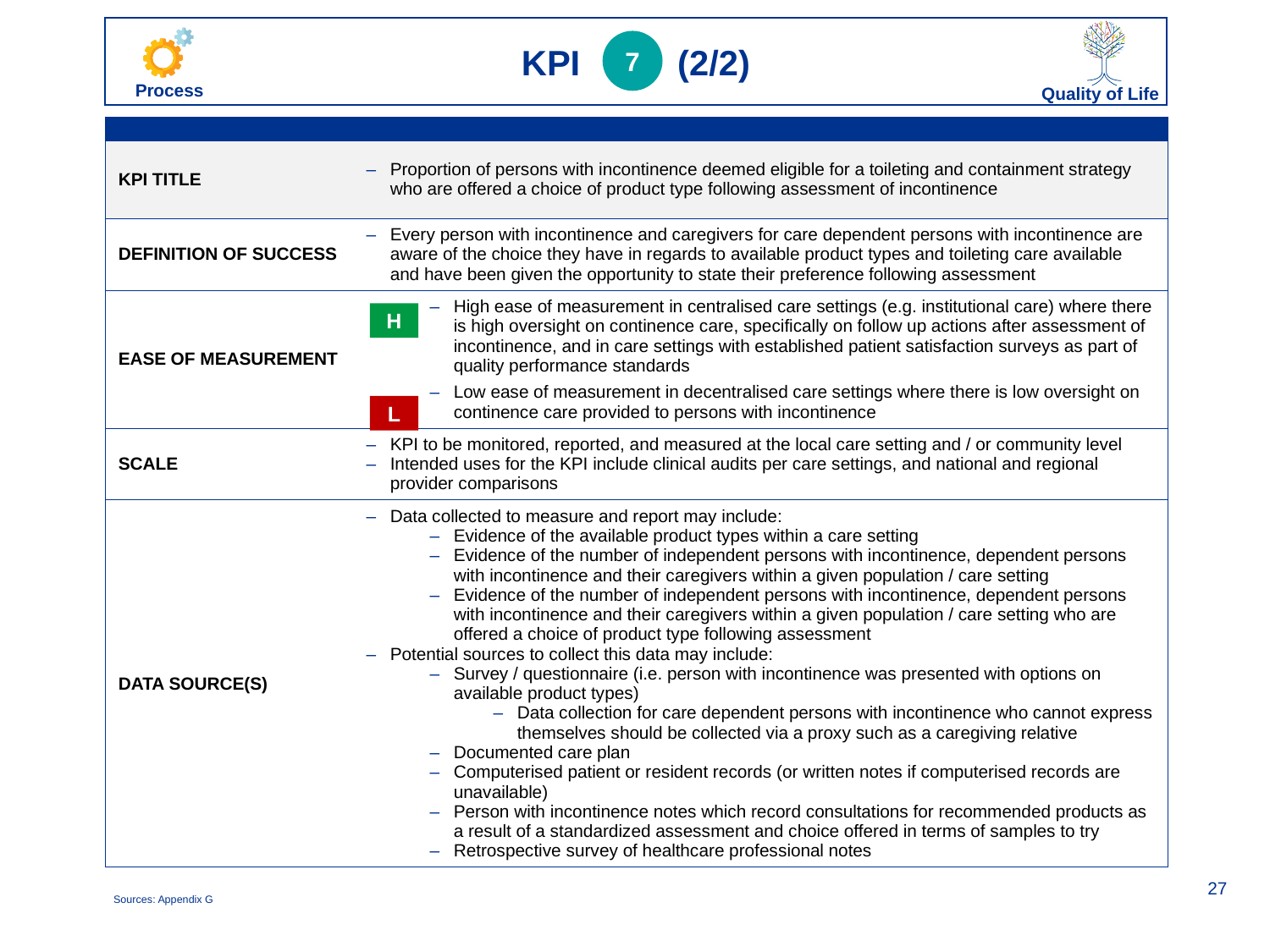

KPI (2/2)
7
Process
Quality of Life
| | |
| --- | --- |
| KPI TITLE | Proportion of persons with incontinence deemed eligible for a toileting and containment strategy who are offered a choice of product type following assessment of incontinence |
| DEFINITION OF SUCCESS | Every person with incontinence and caregivers for care dependent persons with incontinence are aware of the choice they have in regards to available product types and toileting care available and have been given the opportunity to state their preference following assessment |
| EASE OF MEASUREMENT | High ease of measurement in centralised care settings (e.g. institutional care) where there is high oversight on continence care, specifically on follow up actions after assessment of incontinence, and in care settings with established patient satisfaction surveys as part of quality performance standards Low ease of measurement in decentralised care settings where there is low oversight on continence care provided to persons with incontinence |
| SCALE | KPI to be monitored, reported, and measured at the local care setting and / or community level Intended uses for the KPI include clinical audits per care settings, and national and regional provider comparisons |
| DATA SOURCE(S) | Data collected to measure and report may include: Evidence of the available product types within a care setting Evidence of the number of independent persons with incontinence, dependent persons with incontinence and their caregivers within a given population / care setting Evidence of the number of independent persons with incontinence, dependent persons with incontinence and their caregivers within a given population / care setting who are offered a choice of product type following assessment Potential sources to collect this data may include: Survey / questionnaire (i.e. person with incontinence was presented with options on available product types) Data collection for care dependent persons with incontinence who cannot express themselves should be collected via a proxy such as a caregiving relative Documented care plan Computerised patient or resident records (or written notes if computerised records are unavailable) Person with incontinence notes which record consultations for recommended products as a result of a standardized assessment and choice offered in terms of samples to try Retrospective survey of healthcare professional notes |
H
L
Sources: Appendix G

## Slide 28
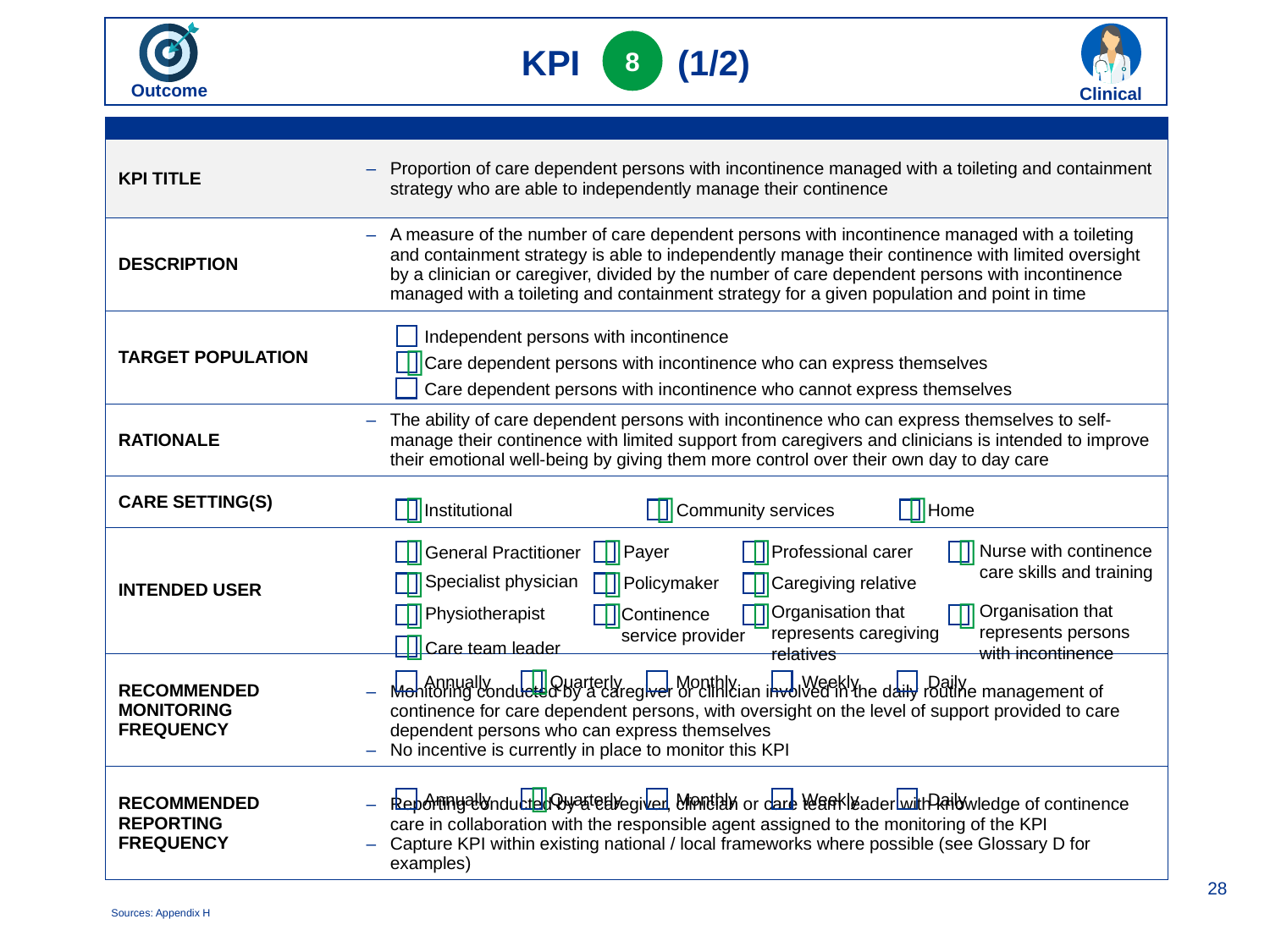

KPI (1/2)
8
Outcome
Clinical
| | |
| --- | --- |
| KPI TITLE | Proportion of care dependent persons with incontinence managed with a toileting and containment strategy who are able to independently manage their continence |
| DESCRIPTION | A measure of the number of care dependent persons with incontinence managed with a toileting and containment strategy is able to independently manage their continence with limited oversight by a clinician or caregiver, divided by the number of care dependent persons with incontinence managed with a toileting and containment strategy for a given population and point in time |
| TARGET POPULATION | |
| RATIONALE | The ability of care dependent persons with incontinence who can express themselves to self-manage their continence with limited support from caregivers and clinicians is intended to improve their emotional well-being by giving them more control over their own day to day care |
| CARE SETTING(S) | |
| INTENDED USER | |
| RECOMMENDED MONITORING FREQUENCY | Monitoring conducted by a caregiver or clinician involved in the daily routine management of continence for care dependent persons, with oversight on the level of support provided to care dependent persons who can express themselves No incentive is currently in place to monitor this KPI |
| RECOMMENDED REPORTING FREQUENCY | Reporting conducted by a caregiver, clinician or care team leader with knowledge of continence care in collaboration with the responsible agent assigned to the monitoring of the KPI Capture KPI within existing national / local frameworks where possible (see Glossary D for examples) |
Independent persons with incontinence
Care dependent persons with incontinence who can express themselves

Care dependent persons with incontinence who cannot express themselves
Institutional
Community services
Home



General Practitioner
Professional carer
Payer
Nurse with continence care skills and training




Specialist physician
Caregiving relative
Policymaker



Physiotherapist




Continence service provider
Organisation that represents caregiving relatives
Organisation that represents persons with incontinence
Care team leader

Annually
Quarterly
Monthly
Weekly
Daily

Annually
Quarterly
Monthly
Weekly
Daily

Sources: Appendix H

## Slide 29
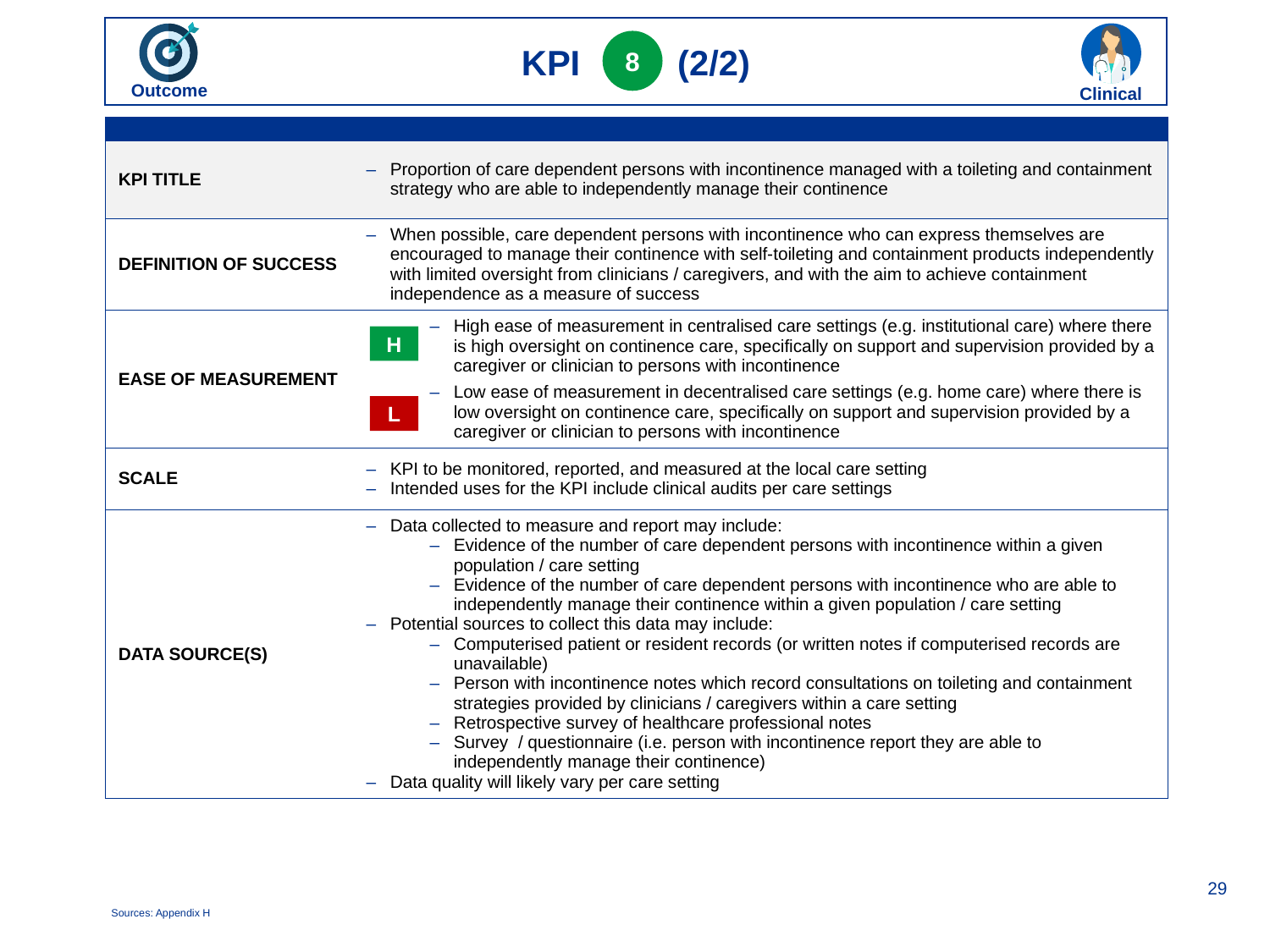

KPI (2/2)
8
Outcome
Clinical
| | |
| --- | --- |
| KPI TITLE | Proportion of care dependent persons with incontinence managed with a toileting and containment strategy who are able to independently manage their continence |
| DEFINITION OF SUCCESS | When possible, care dependent persons with incontinence who can express themselves are encouraged to manage their continence with self-toileting and containment products independently with limited oversight from clinicians / caregivers, and with the aim to achieve containment independence as a measure of success |
| EASE OF MEASUREMENT | High ease of measurement in centralised care settings (e.g. institutional care) where there is high oversight on continence care, specifically on support and supervision provided by a caregiver or clinician to persons with incontinence Low ease of measurement in decentralised care settings (e.g. home care) where there is low oversight on continence care, specifically on support and supervision provided by a caregiver or clinician to persons with incontinence |
| SCALE | KPI to be monitored, reported, and measured at the local care setting Intended uses for the KPI include clinical audits per care settings |
| DATA SOURCE(S) | Data collected to measure and report may include: Evidence of the number of care dependent persons with incontinence within a given population / care setting Evidence of the number of care dependent persons with incontinence who are able to independently manage their continence within a given population / care setting Potential sources to collect this data may include: Computerised patient or resident records (or written notes if computerised records are unavailable) Person with incontinence notes which record consultations on toileting and containment strategies provided by clinicians / caregivers within a care setting Retrospective survey of healthcare professional notes Survey / questionnaire (i.e. person with incontinence report they are able to independently manage their continence) Data quality will likely vary per care setting |
H
L
Sources: Appendix H

## Slide 30
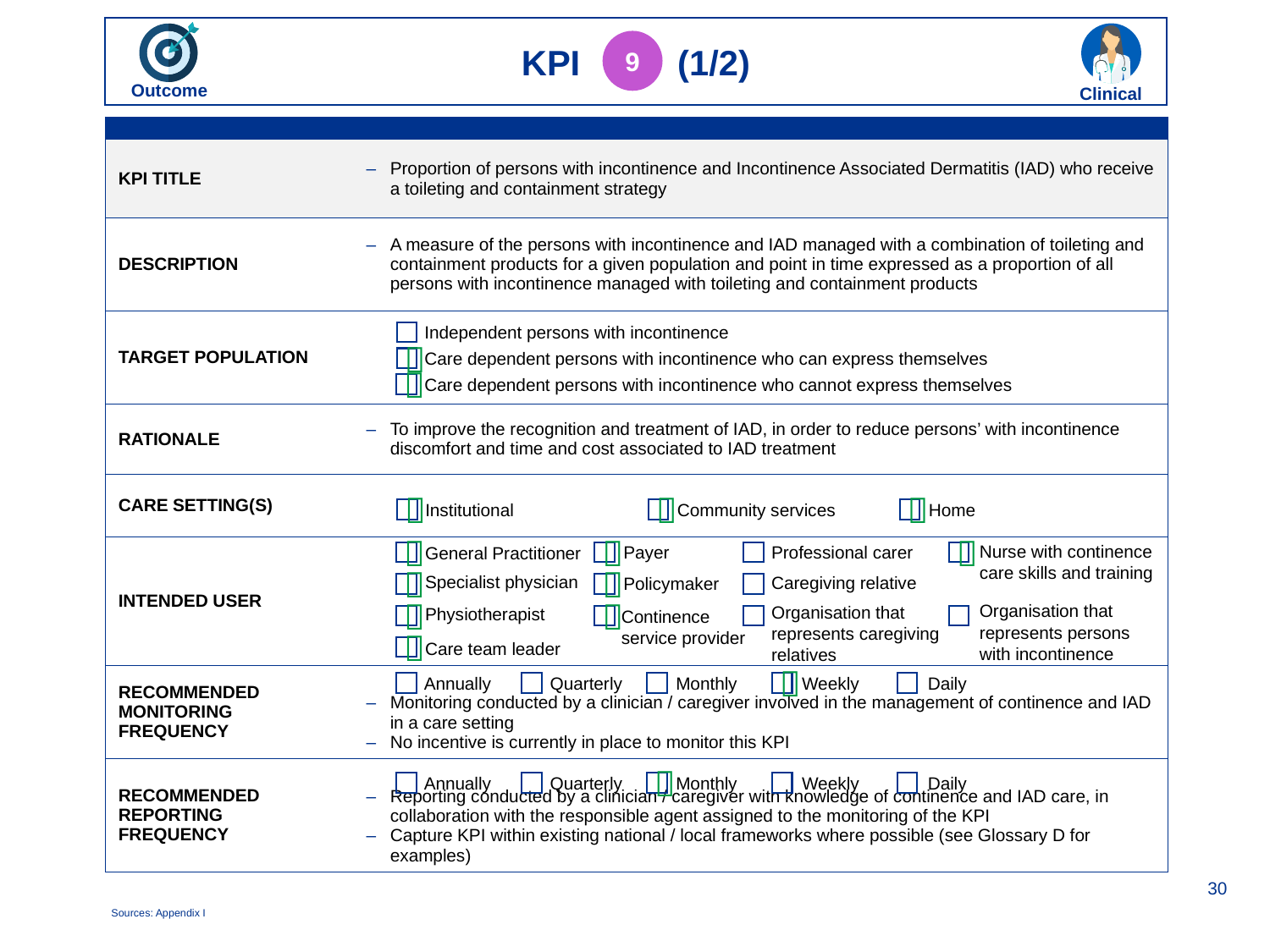

KPI (1/2)
9
Outcome
Clinical
| | |
| --- | --- |
| KPI TITLE | Proportion of persons with incontinence and Incontinence Associated Dermatitis (IAD) who receive a toileting and containment strategy |
| DESCRIPTION | A measure of the persons with incontinence and IAD managed with a combination of toileting and containment products for a given population and point in time expressed as a proportion of all persons with incontinence managed with toileting and containment products |
| TARGET POPULATION | |
| RATIONALE | To improve the recognition and treatment of IAD, in order to reduce persons’ with incontinence discomfort and time and cost associated to IAD treatment |
| CARE SETTING(S) | |
| INTENDED USER | |
| RECOMMENDED MONITORING FREQUENCY | Monitoring conducted by a clinician / caregiver involved in the management of continence and IAD in a care setting No incentive is currently in place to monitor this KPI |
| RECOMMENDED REPORTING FREQUENCY | Reporting conducted by a clinician / caregiver with knowledge of continence and IAD care, in collaboration with the responsible agent assigned to the monitoring of the KPI Capture KPI within existing national / local frameworks where possible (see Glossary D for examples) |
Independent persons with incontinence
Care dependent persons with incontinence who can express themselves

Care dependent persons with incontinence who cannot express themselves

Institutional
Community services
Home



General Practitioner
Professional carer
Payer
Nurse with continence care skills and training



Specialist physician
Caregiving relative
Policymaker


Physiotherapist


Continence service provider
Organisation that represents caregiving relatives
Organisation that represents persons with incontinence
Care team leader

Annually
Quarterly
Monthly
Weekly
Daily

Annually
Quarterly
Monthly
Weekly
Daily

Sources: Appendix I

## Slide 31
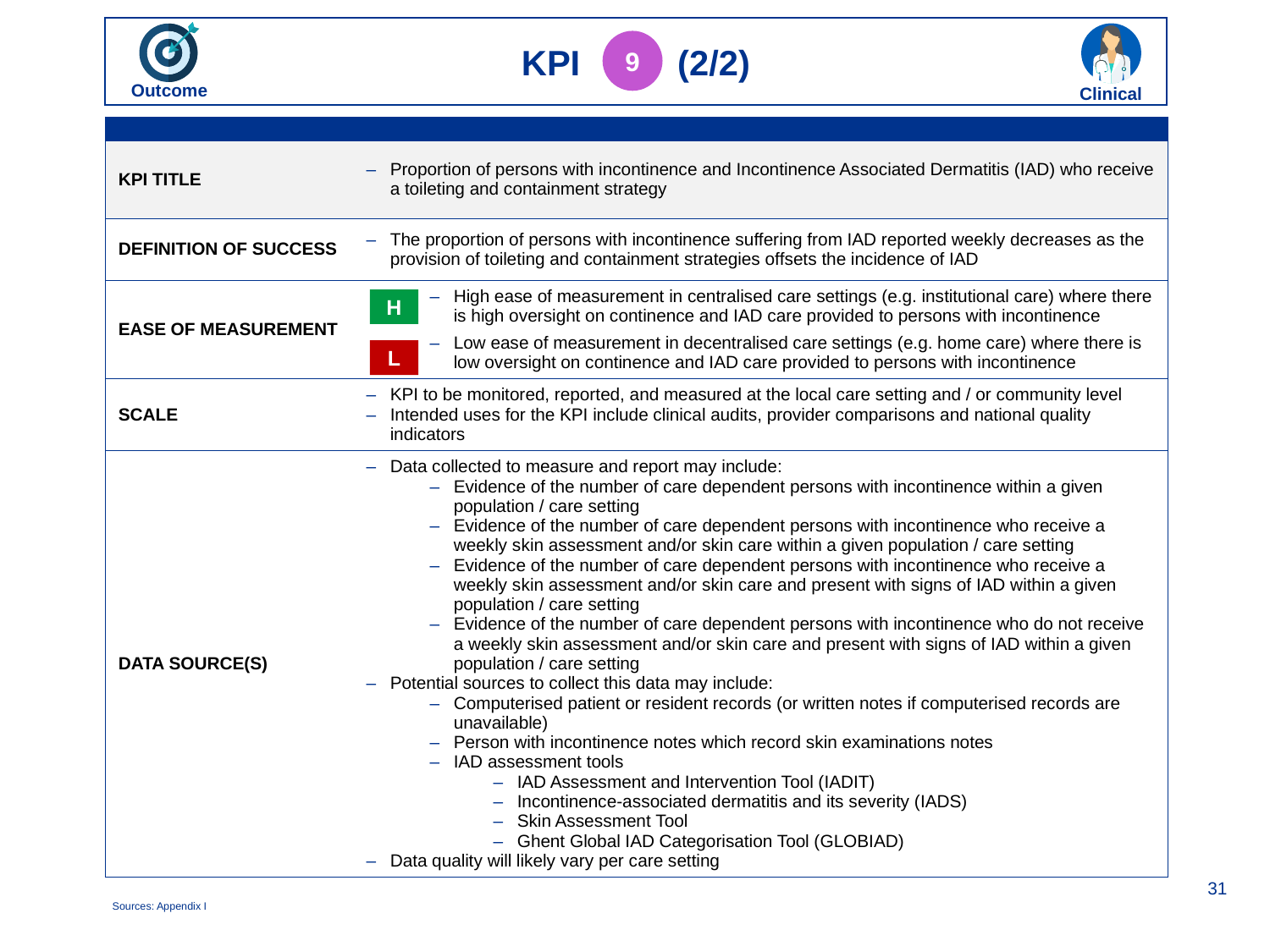

KPI (2/2)
9
Outcome
Clinical
| | |
| --- | --- |
| KPI TITLE | Proportion of persons with incontinence and Incontinence Associated Dermatitis (IAD) who receive a toileting and containment strategy |
| DEFINITION OF SUCCESS | The proportion of persons with incontinence suffering from IAD reported weekly decreases as the provision of toileting and containment strategies offsets the incidence of IAD |
| EASE OF MEASUREMENT | High ease of measurement in centralised care settings (e.g. institutional care) where there is high oversight on continence and IAD care provided to persons with incontinence Low ease of measurement in decentralised care settings (e.g. home care) where there is low oversight on continence and IAD care provided to persons with incontinence |
| SCALE | KPI to be monitored, reported, and measured at the local care setting and / or community level Intended uses for the KPI include clinical audits, provider comparisons and national quality indicators |
| DATA SOURCE(S) | Data collected to measure and report may include: Evidence of the number of care dependent persons with incontinence within a given population / care setting Evidence of the number of care dependent persons with incontinence who receive a weekly skin assessment and/or skin care within a given population / care setting Evidence of the number of care dependent persons with incontinence who receive a weekly skin assessment and/or skin care and present with signs of IAD within a given population / care setting Evidence of the number of care dependent persons with incontinence who do not receive a weekly skin assessment and/or skin care and present with signs of IAD within a given population / care setting Potential sources to collect this data may include: Computerised patient or resident records (or written notes if computerised records are unavailable) Person with incontinence notes which record skin examinations notes IAD assessment tools IAD Assessment and Intervention Tool (IADIT) Incontinence-associated dermatitis and its severity (IADS) Skin Assessment Tool Ghent Global IAD Categorisation Tool (GLOBIAD) Data quality will likely vary per care setting |
H
L
Sources: Appendix I

## Slide 32
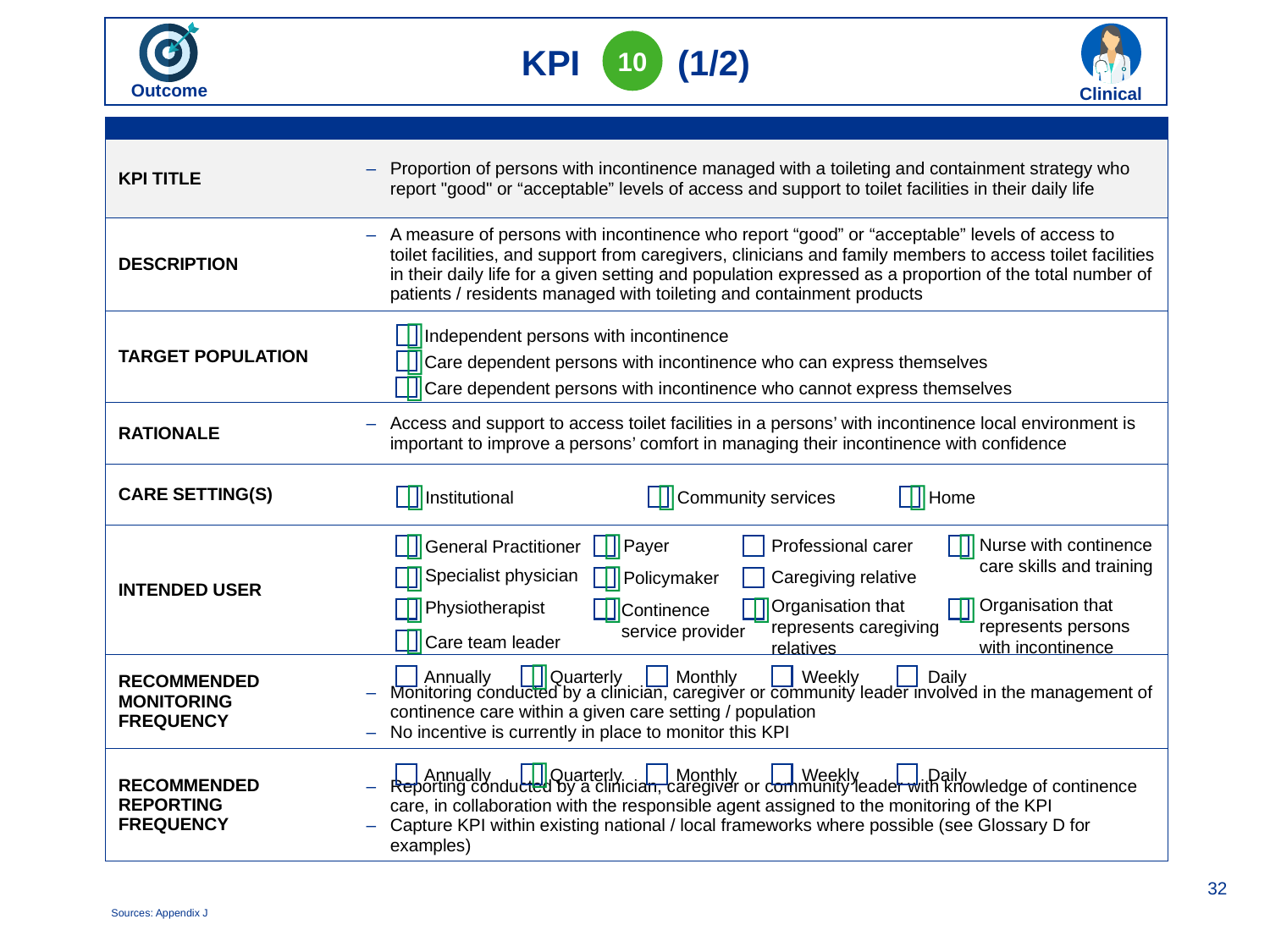

KPI (1/2)
10
Outcome
Clinical
| | |
| --- | --- |
| KPI TITLE | Proportion of persons with incontinence managed with a toileting and containment strategy who report "good" or “acceptable” levels of access and support to toilet facilities in their daily life |
| DESCRIPTION | A measure of persons with incontinence who report “good” or “acceptable” levels of access to toilet facilities, and support from caregivers, clinicians and family members to access toilet facilities in their daily life for a given setting and population expressed as a proportion of the total number of patients / residents managed with toileting and containment products |
| TARGET POPULATION | |
| RATIONALE | Access and support to access toilet facilities in a persons’ with incontinence local environment is important to improve a persons’ comfort in managing their incontinence with confidence |
| CARE SETTING(S) | |
| INTENDED USER | |
| RECOMMENDED MONITORING FREQUENCY | Monitoring conducted by a clinician, caregiver or community leader involved in the management of continence care within a given care setting / population No incentive is currently in place to monitor this KPI |
| RECOMMENDED REPORTING FREQUENCY | Reporting conducted by a clinician, caregiver or community leader with knowledge of continence care, in collaboration with the responsible agent assigned to the monitoring of the KPI Capture KPI within existing national / local frameworks where possible (see Glossary D for examples) |
Independent persons with incontinence

Care dependent persons with incontinence who can express themselves

Care dependent persons with incontinence who cannot express themselves

Institutional
Community services
Home



General Practitioner
Professional carer
Payer
Nurse with continence care skills and training



Specialist physician
Caregiving relative
Policymaker


Physiotherapist




Continence service provider
Organisation that represents caregiving relatives
Organisation that represents persons with incontinence
Care team leader

Annually
Quarterly
Monthly
Weekly
Daily

Annually
Quarterly
Monthly
Weekly
Daily

Sources: Appendix J

## Slide 33
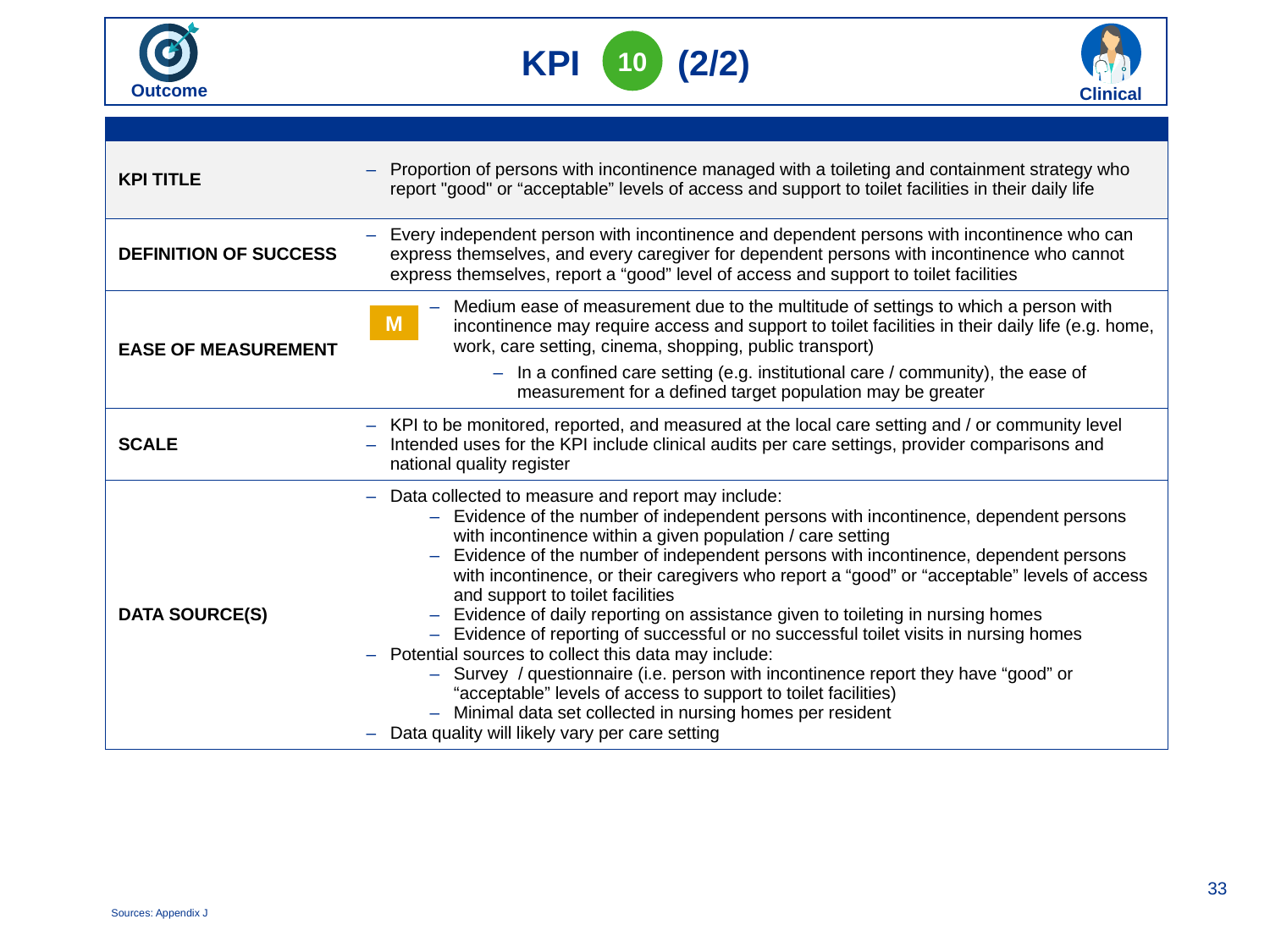

KPI (2/2)
10
Outcome
Clinical
| | |
| --- | --- |
| KPI TITLE | Proportion of persons with incontinence managed with a toileting and containment strategy who report "good" or “acceptable” levels of access and support to toilet facilities in their daily life |
| DEFINITION OF SUCCESS | Every independent person with incontinence and dependent persons with incontinence who can express themselves, and every caregiver for dependent persons with incontinence who cannot express themselves, report a “good” level of access and support to toilet facilities |
| EASE OF MEASUREMENT | Medium ease of measurement due to the multitude of settings to which a person with incontinence may require access and support to toilet facilities in their daily life (e.g. home, work, care setting, cinema, shopping, public transport) In a confined care setting (e.g. institutional care / community), the ease of measurement for a defined target population may be greater |
| SCALE | KPI to be monitored, reported, and measured at the local care setting and / or community level Intended uses for the KPI include clinical audits per care settings, provider comparisons and national quality register |
| DATA SOURCE(S) | Data collected to measure and report may include: Evidence of the number of independent persons with incontinence, dependent persons with incontinence within a given population / care setting Evidence of the number of independent persons with incontinence, dependent persons with incontinence, or their caregivers who report a “good” or “acceptable” levels of access and support to toilet facilities Evidence of daily reporting on assistance given to toileting in nursing homes Evidence of reporting of successful or no successful toilet visits in nursing homes Potential sources to collect this data may include: Survey / questionnaire (i.e. person with incontinence report they have “good” or “acceptable” levels of access to support to toilet facilities) Minimal data set collected in nursing homes per resident Data quality will likely vary per care setting |
M
Sources: Appendix J

## Slide 34
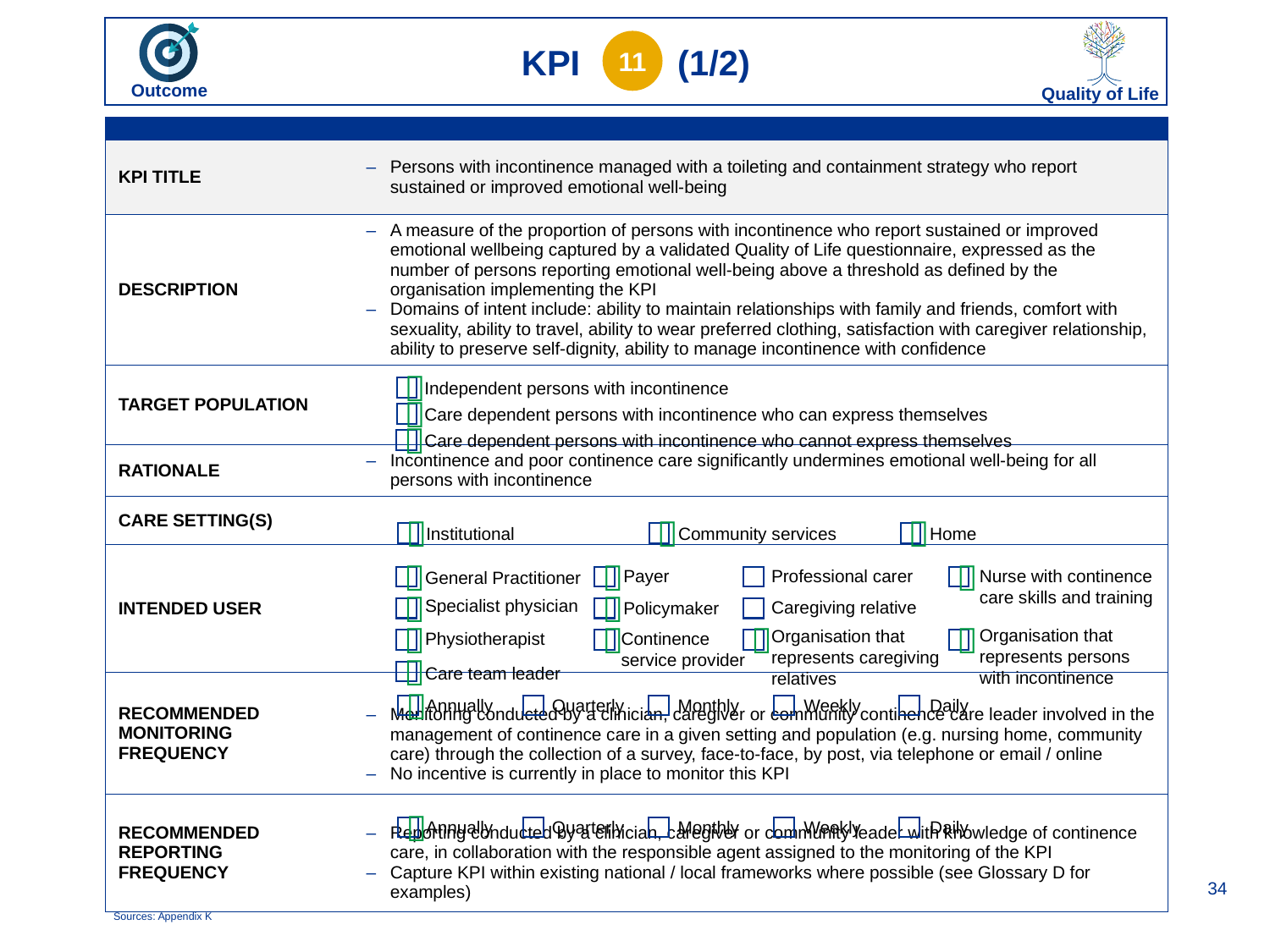

KPI (1/2)
11
Outcome
Quality of Life
| | |
| --- | --- |
| KPI TITLE | Persons with incontinence managed with a toileting and containment strategy who report sustained or improved emotional well-being |
| DESCRIPTION | A measure of the proportion of persons with incontinence who report sustained or improved emotional wellbeing captured by a validated Quality of Life questionnaire, expressed as the number of persons reporting emotional well-being above a threshold as defined by the organisation implementing the KPI Domains of intent include: ability to maintain relationships with family and friends, comfort with sexuality, ability to travel, ability to wear preferred clothing, satisfaction with caregiver relationship, ability to preserve self-dignity, ability to manage incontinence with confidence |
| TARGET POPULATION | |
| RATIONALE | Incontinence and poor continence care significantly undermines emotional well-being for all persons with incontinence |
| CARE SETTING(S) | |
| INTENDED USER | |
| RECOMMENDED MONITORING FREQUENCY | Monitoring conducted by a clinician, caregiver or community continence care leader involved in the management of continence care in a given setting and population (e.g. nursing home, community care) through the collection of a survey, face-to-face, by post, via telephone or email / online No incentive is currently in place to monitor this KPI |
| RECOMMENDED REPORTING FREQUENCY | Reporting conducted by a clinician, caregiver or community leader with knowledge of continence care, in collaboration with the responsible agent assigned to the monitoring of the KPI Capture KPI within existing national / local frameworks where possible (see Glossary D for examples) |
Independent persons with incontinence

Care dependent persons with incontinence who can express themselves

Care dependent persons with incontinence who cannot express themselves

Institutional
Community services
Home



General Practitioner
Professional carer
Payer
Nurse with continence care skills and training



Specialist physician
Caregiving relative
Policymaker


Physiotherapist




Continence service provider
Organisation that represents caregiving relatives
Organisation that represents persons with incontinence
Care team leader

Annually
Quarterly
Monthly
Weekly
Daily

Annually
Quarterly
Monthly
Weekly
Daily

Sources: Appendix K

## Slide 35
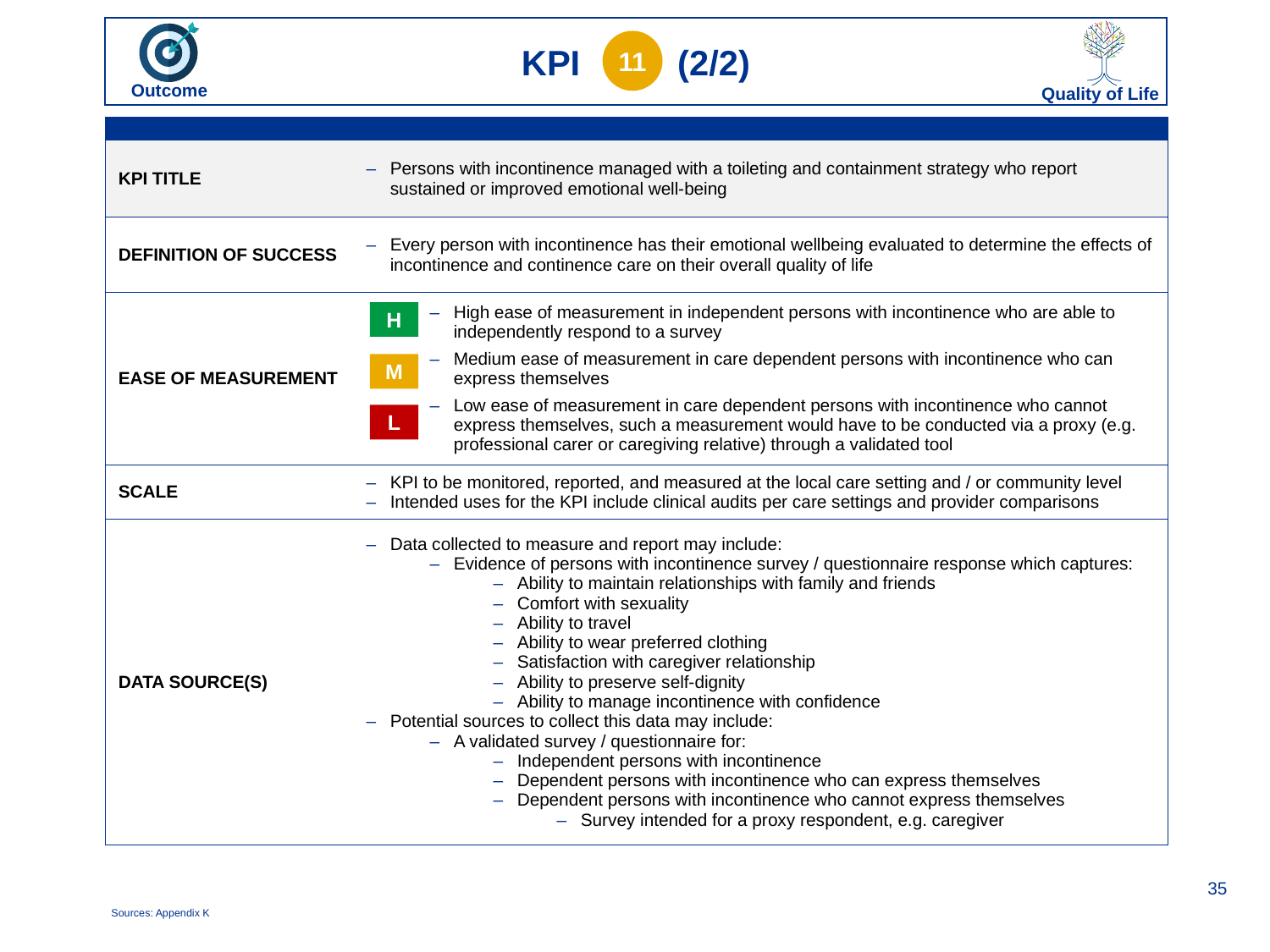

KPI (2/2)
11
Outcome
Quality of Life
| | |
| --- | --- |
| KPI TITLE | Persons with incontinence managed with a toileting and containment strategy who report sustained or improved emotional well-being |
| DEFINITION OF SUCCESS | Every person with incontinence has their emotional wellbeing evaluated to determine the effects of incontinence and continence care on their overall quality of life |
| EASE OF MEASUREMENT | High ease of measurement in independent persons with incontinence who are able to independently respond to a survey Medium ease of measurement in care dependent persons with incontinence who can express themselves Low ease of measurement in care dependent persons with incontinence who cannot express themselves, such a measurement would have to be conducted via a proxy (e.g. professional carer or caregiving relative) through a validated tool |
| SCALE | KPI to be monitored, reported, and measured at the local care setting and / or community level Intended uses for the KPI include clinical audits per care settings and provider comparisons |
| DATA SOURCE(S) | Data collected to measure and report may include: Evidence of persons with incontinence survey / questionnaire response which captures: Ability to maintain relationships with family and friends Comfort with sexuality Ability to travel Ability to wear preferred clothing Satisfaction with caregiver relationship Ability to preserve self-dignity Ability to manage incontinence with confidence Potential sources to collect this data may include: A validated survey / questionnaire for: Independent persons with incontinence Dependent persons with incontinence who can express themselves Dependent persons with incontinence who cannot express themselves Survey intended for a proxy respondent, e.g. caregiver |
H
M
L
Sources: Appendix K

## Slide 36
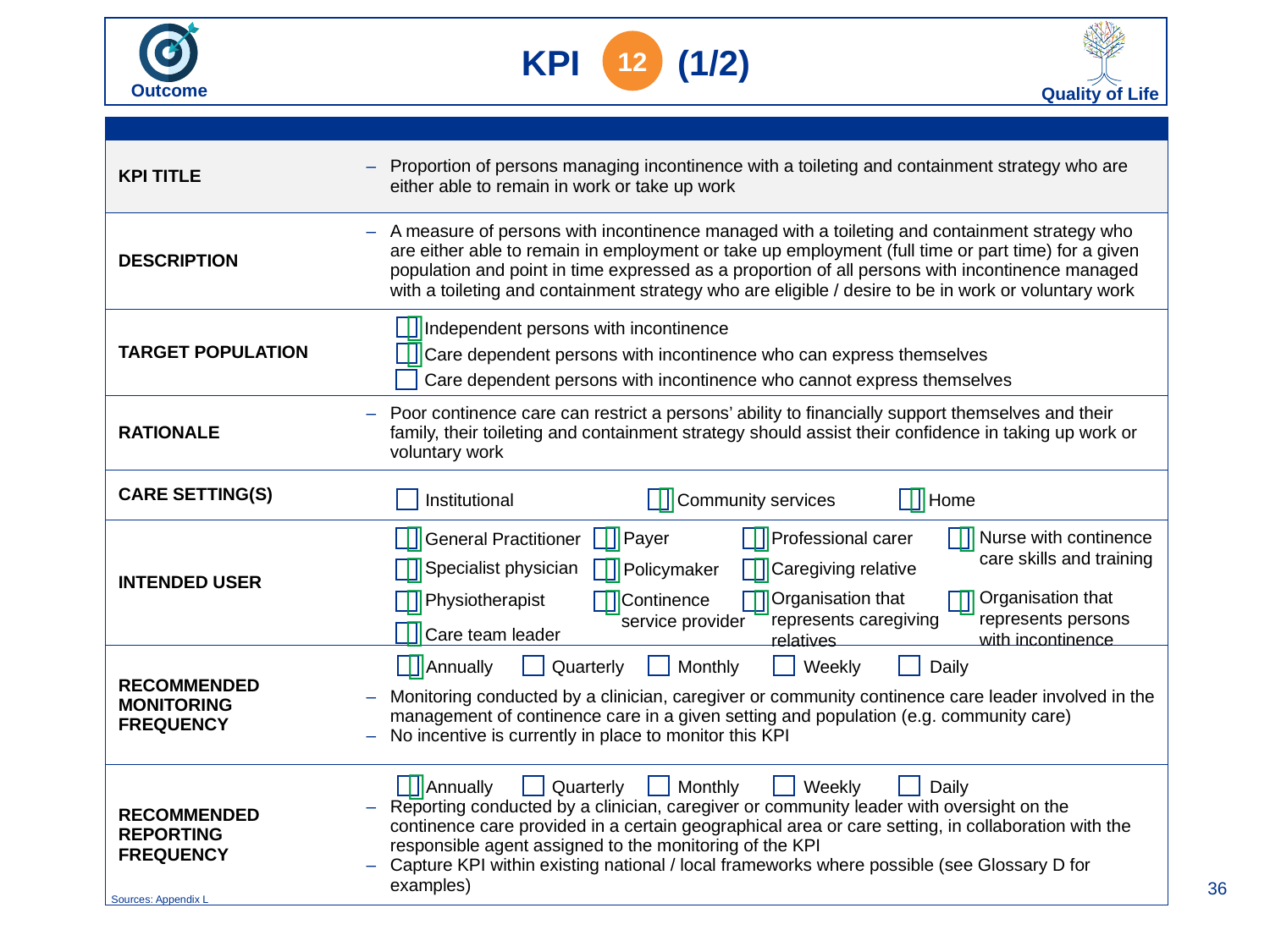

KPI (1/2)
12
Outcome
Quality of Life
| | |
| --- | --- |
| KPI TITLE | Proportion of persons managing incontinence with a toileting and containment strategy who are either able to remain in work or take up work |
| DESCRIPTION | A measure of persons with incontinence managed with a toileting and containment strategy who are either able to remain in employment or take up employment (full time or part time) for a given population and point in time expressed as a proportion of all persons with incontinence managed with a toileting and containment strategy who are eligible / desire to be in work or voluntary work |
| TARGET POPULATION | |
| RATIONALE | Poor continence care can restrict a persons’ ability to financially support themselves and their family, their toileting and containment strategy should assist their confidence in taking up work or voluntary work |
| CARE SETTING(S) | |
| INTENDED USER | |
| RECOMMENDED MONITORING FREQUENCY | Monitoring conducted by a clinician, caregiver or community continence care leader involved in the management of continence care in a given setting and population (e.g. community care) No incentive is currently in place to monitor this KPI |
| RECOMMENDED REPORTING FREQUENCY | Reporting conducted by a clinician, caregiver or community leader with oversight on the continence care provided in a certain geographical area or care setting, in collaboration with the responsible agent assigned to the monitoring of the KPI Capture KPI within existing national / local frameworks where possible (see Glossary D for examples) |
Independent persons with incontinence

Care dependent persons with incontinence who can express themselves

Care dependent persons with incontinence who cannot express themselves
Institutional
Community services
Home


General Practitioner
Professional carer
Payer
Nurse with continence care skills and training




Specialist physician
Caregiving relative
Policymaker



Physiotherapist




Continence service provider
Organisation that represents caregiving relatives
Organisation that represents persons with incontinence
Care team leader

Annually
Quarterly
Monthly
Weekly
Daily

Annually
Quarterly
Monthly
Weekly
Daily

Sources: Appendix L

## Slide 37
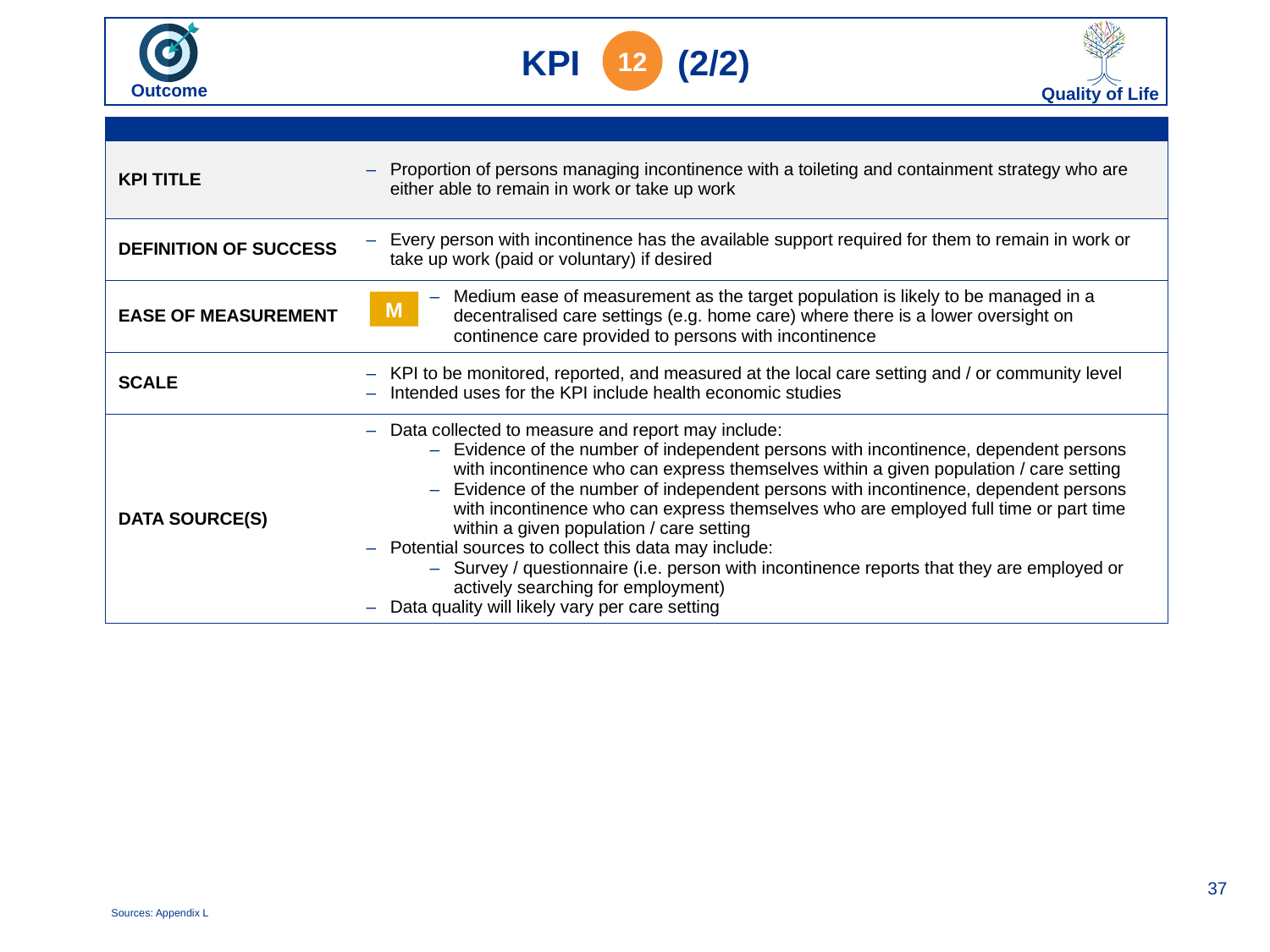

KPI (2/2)
12
Outcome
Quality of Life
| | |
| --- | --- |
| KPI TITLE | Proportion of persons managing incontinence with a toileting and containment strategy who are either able to remain in work or take up work |
| DEFINITION OF SUCCESS | Every person with incontinence has the available support required for them to remain in work or take up work (paid or voluntary) if desired |
| EASE OF MEASUREMENT | Medium ease of measurement as the target population is likely to be managed in a decentralised care settings (e.g. home care) where there is a lower oversight on continence care provided to persons with incontinence |
| SCALE | KPI to be monitored, reported, and measured at the local care setting and / or community level Intended uses for the KPI include health economic studies |
| DATA SOURCE(S) | Data collected to measure and report may include: Evidence of the number of independent persons with incontinence, dependent persons with incontinence who can express themselves within a given population / care setting Evidence of the number of independent persons with incontinence, dependent persons with incontinence who can express themselves who are employed full time or part time within a given population / care setting Potential sources to collect this data may include: Survey / questionnaire (i.e. person with incontinence reports that they are employed or actively searching for employment) Data quality will likely vary per care setting |
M
Sources: Appendix L

## Slide 38
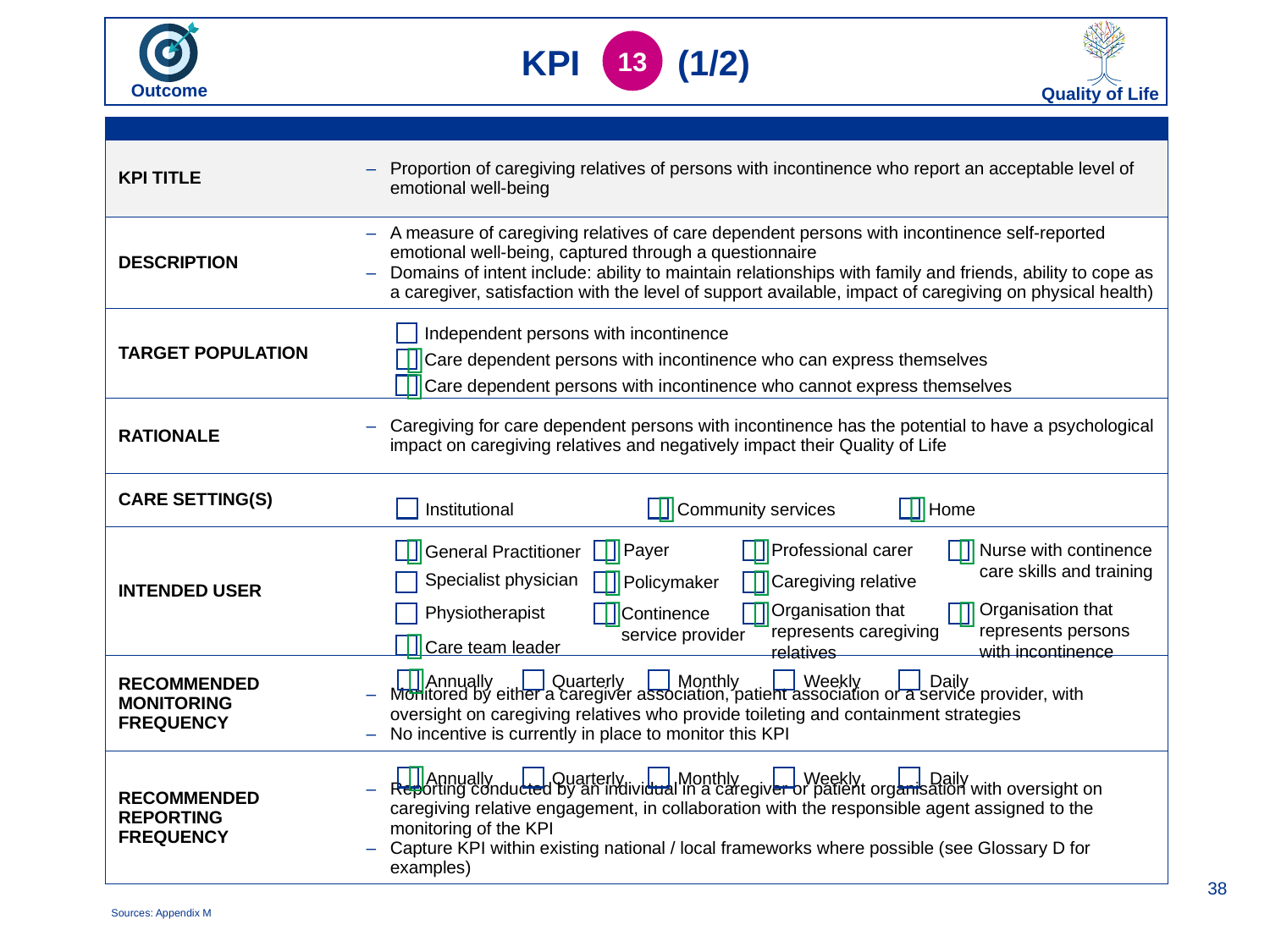

KPI (1/2)
13
Outcome
Quality of Life
| | |
| --- | --- |
| KPI TITLE | Proportion of caregiving relatives of persons with incontinence who report an acceptable level of emotional well-being |
| DESCRIPTION | A measure of caregiving relatives of care dependent persons with incontinence self-reported emotional well-being, captured through a questionnaire Domains of intent include: ability to maintain relationships with family and friends, ability to cope as a caregiver, satisfaction with the level of support available, impact of caregiving on physical health) |
| TARGET POPULATION | |
| RATIONALE | Caregiving for care dependent persons with incontinence has the potential to have a psychological impact on caregiving relatives and negatively impact their Quality of Life |
| CARE SETTING(S) | |
| INTENDED USER | |
| RECOMMENDED MONITORING FREQUENCY | Monitored by either a caregiver association, patient association or a service provider, with oversight on caregiving relatives who provide toileting and containment strategies No incentive is currently in place to monitor this KPI |
| RECOMMENDED REPORTING FREQUENCY | Reporting conducted by an individual in a caregiver or patient organisation with oversight on caregiving relative engagement, in collaboration with the responsible agent assigned to the monitoring of the KPI Capture KPI within existing national / local frameworks where possible (see Glossary D for examples) |
Independent persons with incontinence
Care dependent persons with incontinence who can express themselves

Care dependent persons with incontinence who cannot express themselves

Institutional
Community services
Home


General Practitioner
Professional carer
Payer
Nurse with continence care skills and training




Specialist physician
Caregiving relative
Policymaker


Physiotherapist



Continence service provider
Organisation that represents caregiving relatives
Organisation that represents persons with incontinence
Care team leader

Annually
Quarterly
Monthly
Weekly
Daily

Annually
Quarterly
Monthly
Weekly
Daily

Sources: Appendix M

## Slide 39
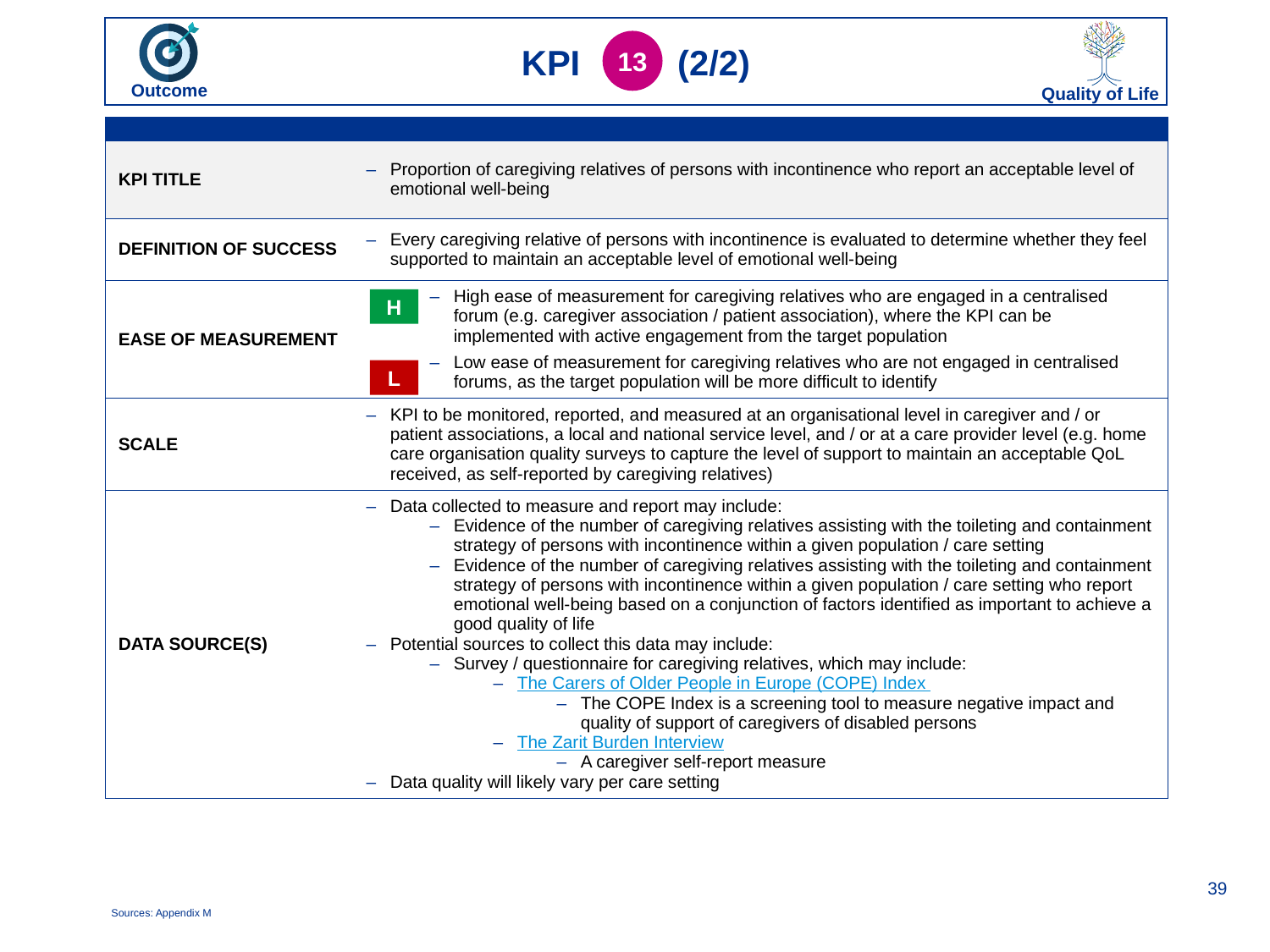

KPI (2/2)
13
Outcome
Quality of Life
| | |
| --- | --- |
| KPI TITLE | Proportion of caregiving relatives of persons with incontinence who report an acceptable level of emotional well-being |
| DEFINITION OF SUCCESS | Every caregiving relative of persons with incontinence is evaluated to determine whether they feel supported to maintain an acceptable level of emotional well-being |
| EASE OF MEASUREMENT | High ease of measurement for caregiving relatives who are engaged in a centralised forum (e.g. caregiver association / patient association), where the KPI can be implemented with active engagement from the target population Low ease of measurement for caregiving relatives who are not engaged in centralised forums, as the target population will be more difficult to identify |
| SCALE | KPI to be monitored, reported, and measured at an organisational level in caregiver and / or patient associations, a local and national service level, and / or at a care provider level (e.g. home care organisation quality surveys to capture the level of support to maintain an acceptable QoL received, as self-reported by caregiving relatives) |
| DATA SOURCE(S) | Data collected to measure and report may include: Evidence of the number of caregiving relatives assisting with the toileting and containment strategy of persons with incontinence within a given population / care setting Evidence of the number of caregiving relatives assisting with the toileting and containment strategy of persons with incontinence within a given population / care setting who report emotional well-being based on a conjunction of factors identified as important to achieve a good quality of life Potential sources to collect this data may include: Survey / questionnaire for caregiving relatives, which may include: The Carers of Older People in Europe (COPE) Index The COPE Index is a screening tool to measure negative impact and quality of support of caregivers of disabled persons The Zarit Burden Interview A caregiver self-report measure Data quality will likely vary per care setting |
H
L
Sources: Appendix M

## Slide 40
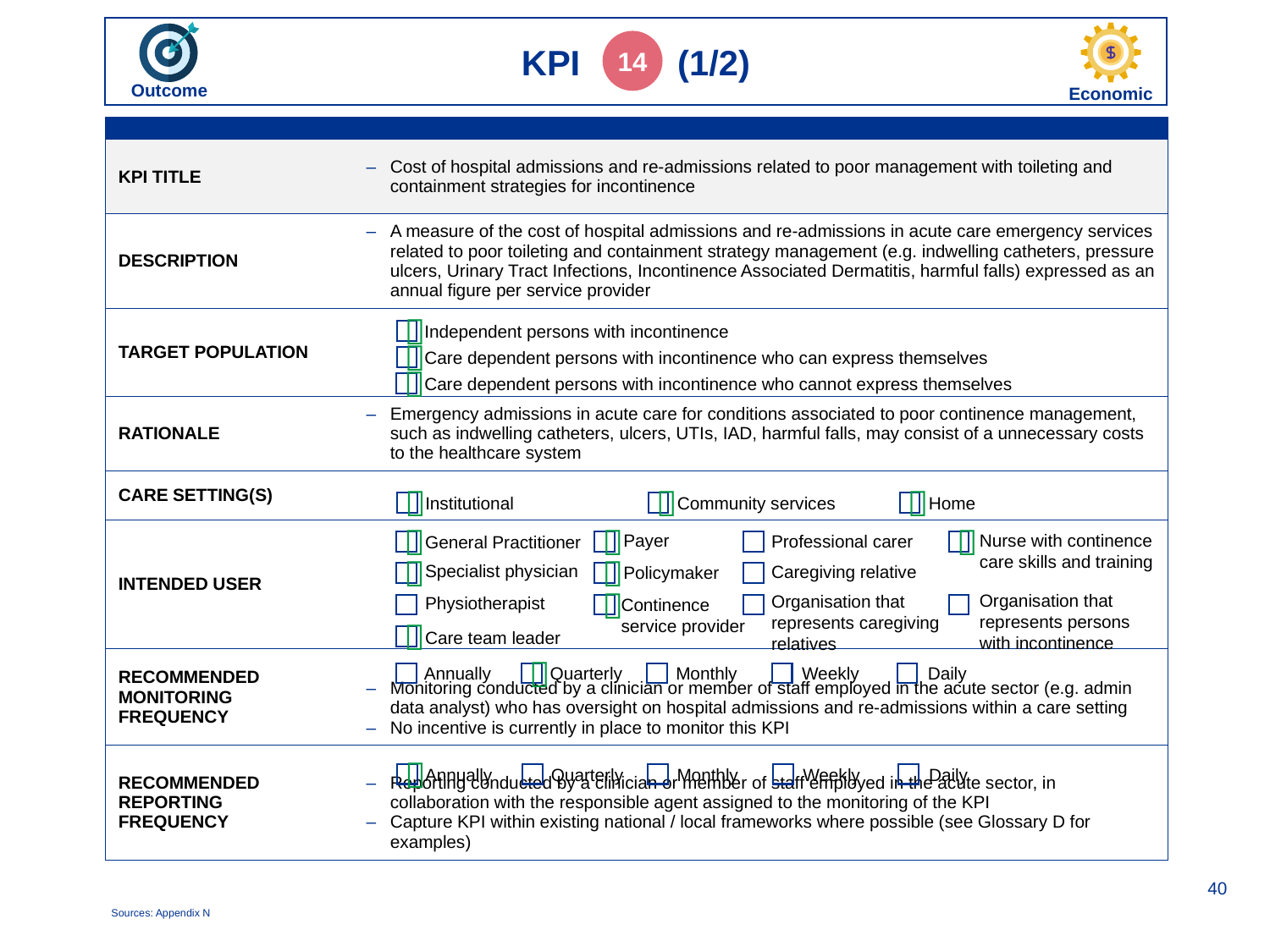

KPI (1/2)
14
Outcome
Economic
| | |
| --- | --- |
| KPI TITLE | Cost of hospital admissions and re-admissions related to poor management with toileting and containment strategies for incontinence |
| DESCRIPTION | A measure of the cost of hospital admissions and re-admissions in acute care emergency services related to poor toileting and containment strategy management (e.g. indwelling catheters, pressure ulcers, Urinary Tract Infections, Incontinence Associated Dermatitis, harmful falls) expressed as an annual figure per service provider |
| TARGET POPULATION | |
| RATIONALE | Emergency admissions in acute care for conditions associated to poor continence management, such as indwelling catheters, ulcers, UTIs, IAD, harmful falls, may consist of a unnecessary costs to the healthcare system |
| CARE SETTING(S) | |
| INTENDED USER | |
| RECOMMENDED MONITORING FREQUENCY | Monitoring conducted by a clinician or member of staff employed in the acute sector (e.g. admin data analyst) who has oversight on hospital admissions and re-admissions within a care setting No incentive is currently in place to monitor this KPI |
| RECOMMENDED REPORTING FREQUENCY | Reporting conducted by a clinician or member of staff employed in the acute sector, in collaboration with the responsible agent assigned to the monitoring of the KPI Capture KPI within existing national / local frameworks where possible (see Glossary D for examples) |
Independent persons with incontinence

Care dependent persons with incontinence who can express themselves

Care dependent persons with incontinence who cannot express themselves

Institutional
Community services
Home



General Practitioner
Professional carer
Payer
Nurse with continence care skills and training



Specialist physician
Caregiving relative
Policymaker


Physiotherapist

Continence service provider
Organisation that represents caregiving relatives
Organisation that represents persons with incontinence
Care team leader

Annually
Quarterly
Monthly
Weekly
Daily

Annually
Quarterly
Monthly
Weekly
Daily

Sources: Appendix N

## Slide 41
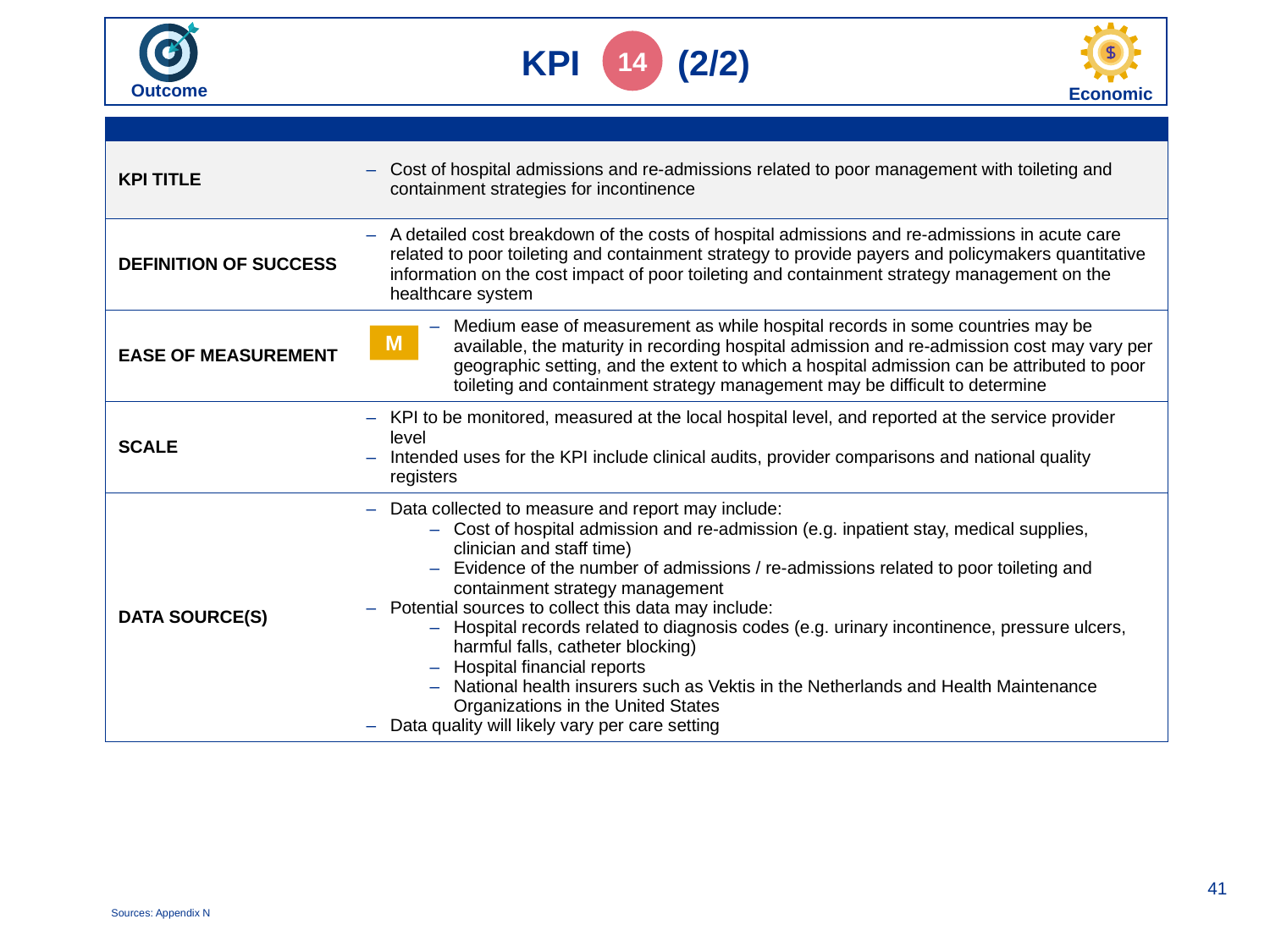

KPI (2/2)
14
Outcome
Economic
| | |
| --- | --- |
| KPI TITLE | Cost of hospital admissions and re-admissions related to poor management with toileting and containment strategies for incontinence |
| DEFINITION OF SUCCESS | A detailed cost breakdown of the costs of hospital admissions and re-admissions in acute care related to poor toileting and containment strategy to provide payers and policymakers quantitative information on the cost impact of poor toileting and containment strategy management on the healthcare system |
| EASE OF MEASUREMENT | Medium ease of measurement as while hospital records in some countries may be available, the maturity in recording hospital admission and re-admission cost may vary per geographic setting, and the extent to which a hospital admission can be attributed to poor toileting and containment strategy management may be difficult to determine |
| SCALE | KPI to be monitored, measured at the local hospital level, and reported at the service provider level Intended uses for the KPI include clinical audits, provider comparisons and national quality registers |
| DATA SOURCE(S) | Data collected to measure and report may include: Cost of hospital admission and re-admission (e.g. inpatient stay, medical supplies, clinician and staff time) Evidence of the number of admissions / re-admissions related to poor toileting and containment strategy management Potential sources to collect this data may include: Hospital records related to diagnosis codes (e.g. urinary incontinence, pressure ulcers, harmful falls, catheter blocking) Hospital financial reports National health insurers such as Vektis in the Netherlands and Health Maintenance Organizations in the United States Data quality will likely vary per care setting |
M
Sources: Appendix N

## Slide 42
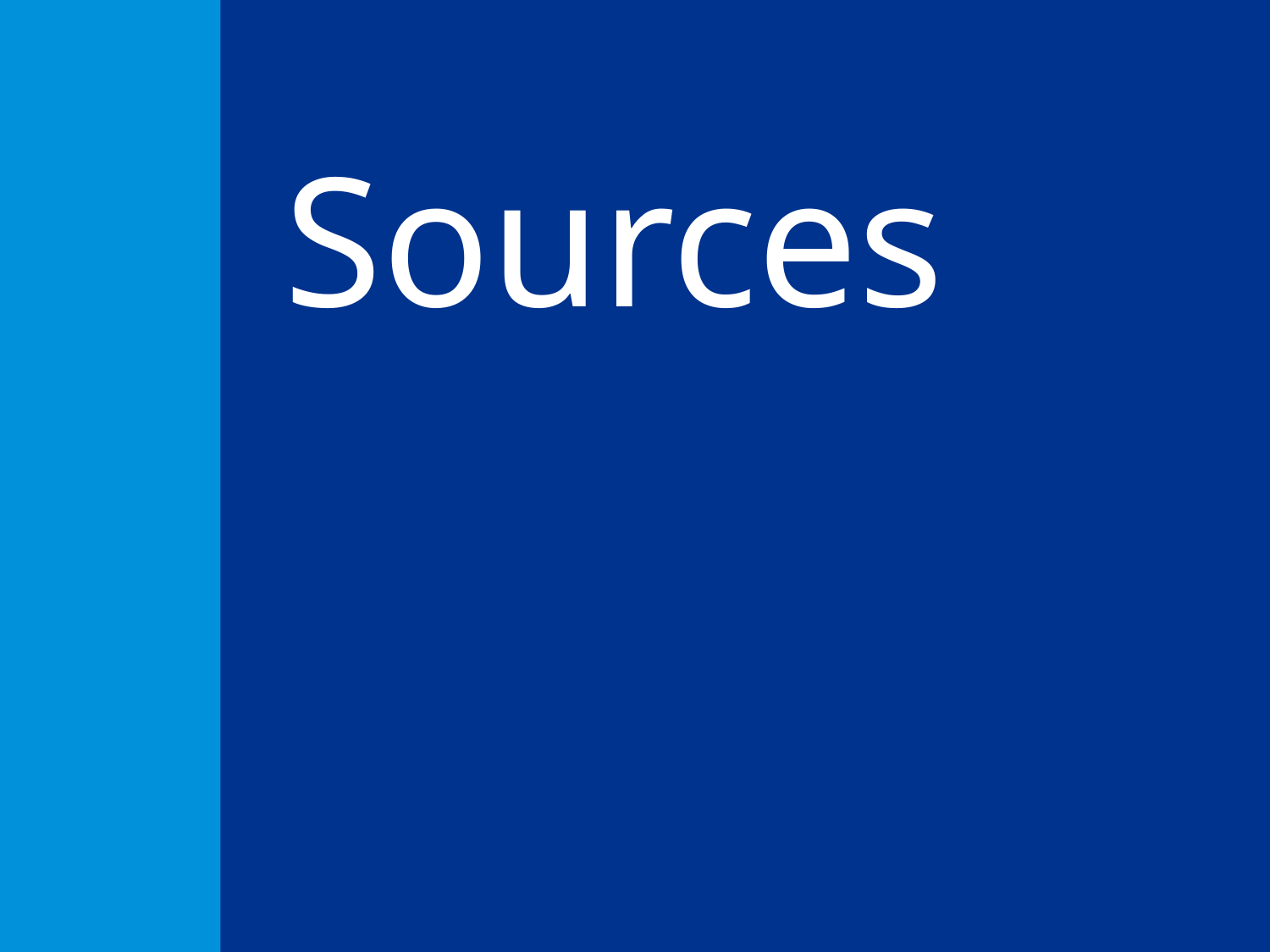

# Sources

## Slide 43
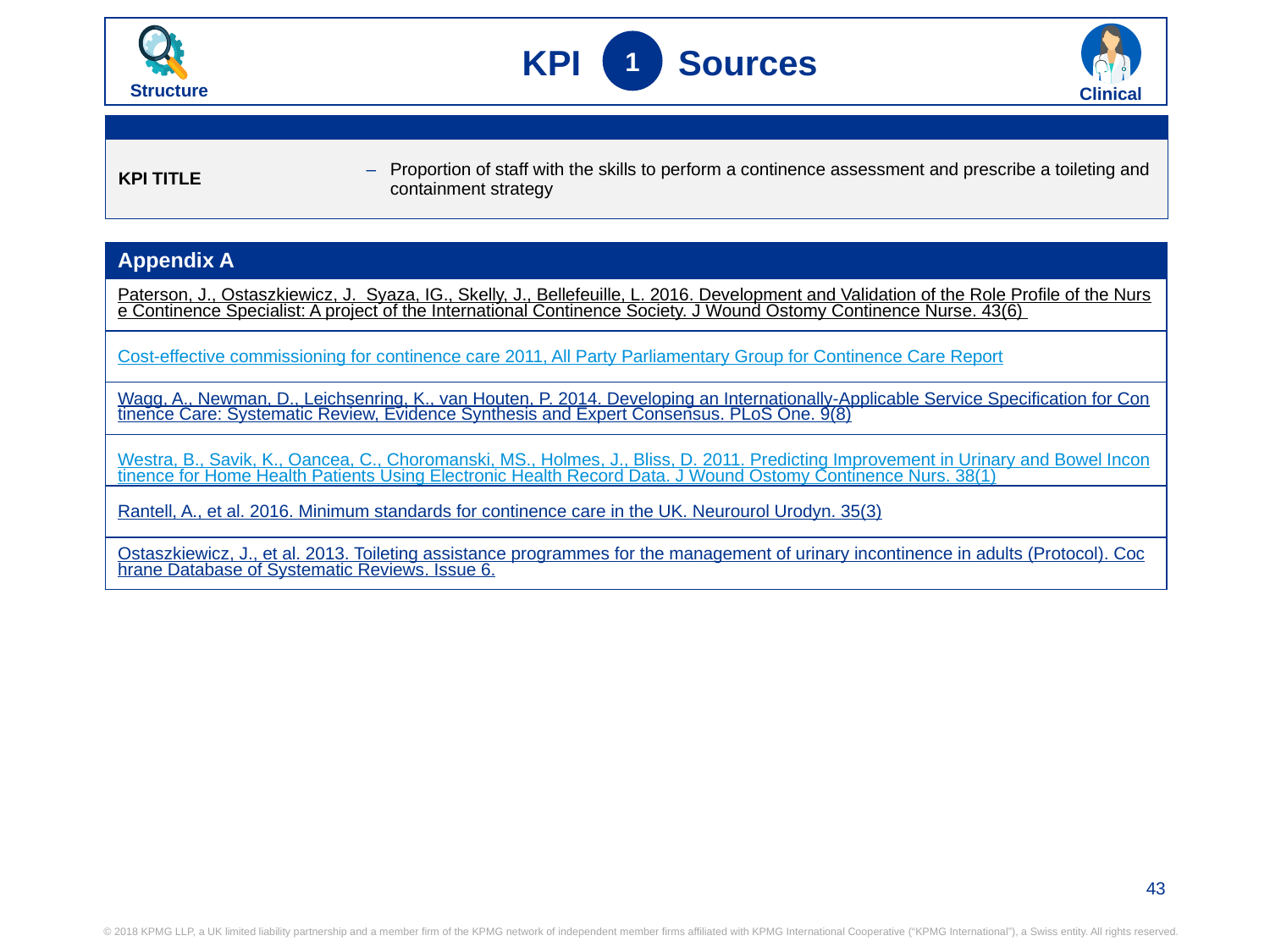

KPI Sources
1
Structure
Clinical
| | |
| --- | --- |
| KPI TITLE | Proportion of staff with the skills to perform a continence assessment and prescribe a toileting and containment strategy |
| Appendix A |
| --- |
| Paterson, J., Ostaszkiewicz, J. Syaza, IG., Skelly, J., Bellefeuille, L. 2016. Development and Validation of the Role Profile of the Nurse Continence Specialist: A project of the International Continence Society. J Wound Ostomy Continence Nurse. 43(6) |
| Cost-effective commissioning for continence care 2011, All Party Parliamentary Group for Continence Care Report |
| Wagg, A., Newman, D., Leichsenring, K., van Houten, P. 2014. Developing an Internationally-Applicable Service Specification for Continence Care: Systematic Review, Evidence Synthesis and Expert Consensus. PLoS One. 9(8) |
| Westra, B., Savik, K., Oancea, C., Choromanski, MS., Holmes, J., Bliss, D. 2011. Predicting Improvement in Urinary and Bowel Incontinence for Home Health Patients Using Electronic Health Record Data. J Wound Ostomy Continence Nurs. 38(1) |
| Rantell, A., et al. 2016. Minimum standards for continence care in the UK. Neurourol Urodyn. 35(3) |
| Ostaszkiewicz, J., et al. 2013. Toileting assistance programmes for the management of urinary incontinence in adults (Protocol). Cochrane Database of Systematic Reviews. Issue 6. |

## Slide 44
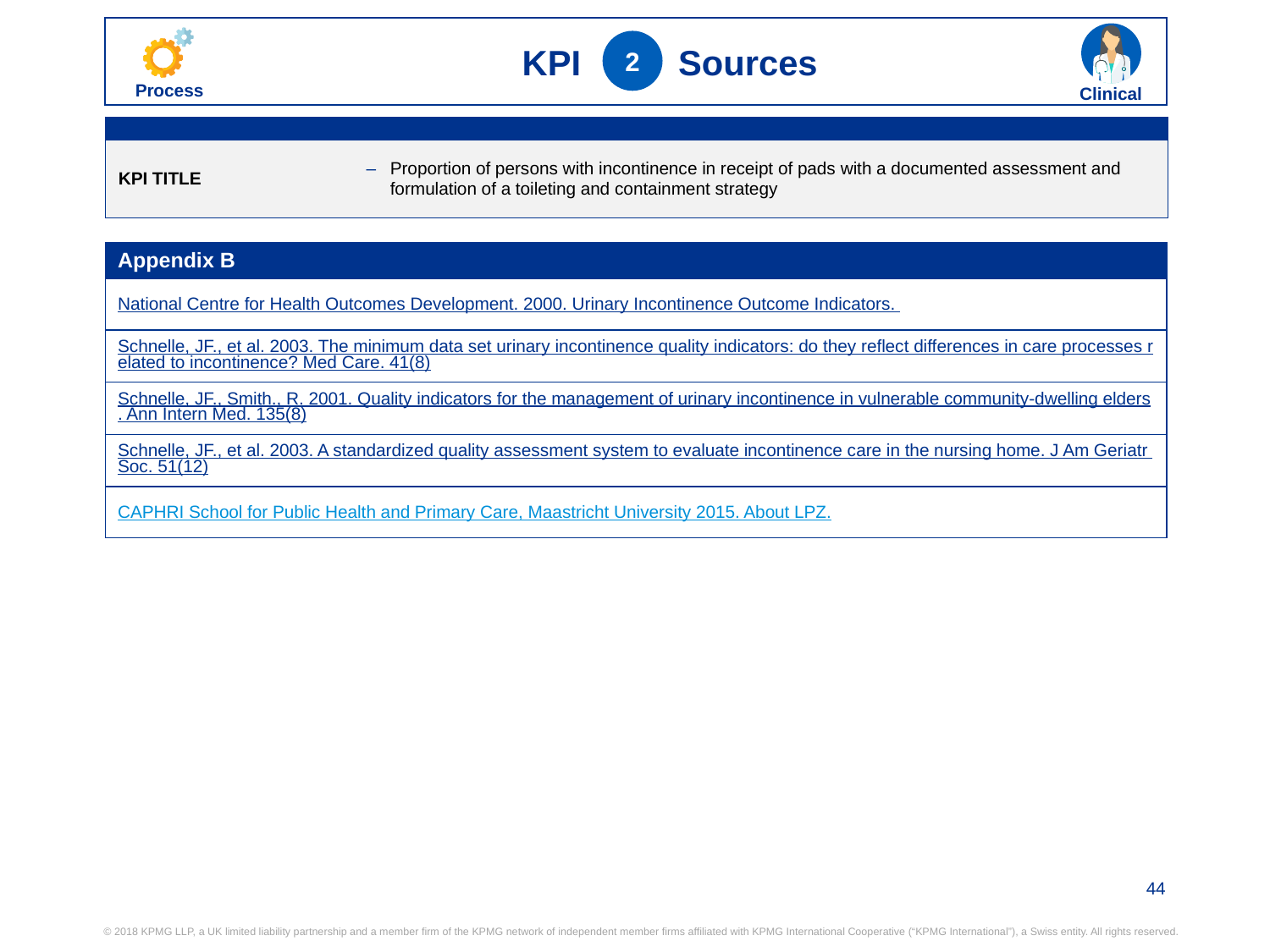

KPI Sources
2
Process
Clinical
| | |
| --- | --- |
| KPI TITLE | Proportion of persons with incontinence in receipt of pads with a documented assessment and formulation of a toileting and containment strategy |
| Appendix B |
| --- |
| National Centre for Health Outcomes Development. 2000. Urinary Incontinence Outcome Indicators. |
| Schnelle, JF., et al. 2003. The minimum data set urinary incontinence quality indicators: do they reflect differences in care processes related to incontinence? Med Care. 41(8) |
| Schnelle, JF., Smith., R. 2001. Quality indicators for the management of urinary incontinence in vulnerable community-dwelling elders. Ann Intern Med. 135(8) |
| Schnelle, JF., et al. 2003. A standardized quality assessment system to evaluate incontinence care in the nursing home. J Am Geriatr Soc. 51(12) |
| CAPHRI School for Public Health and Primary Care, Maastricht University 2015. About LPZ. |

## Slide 45
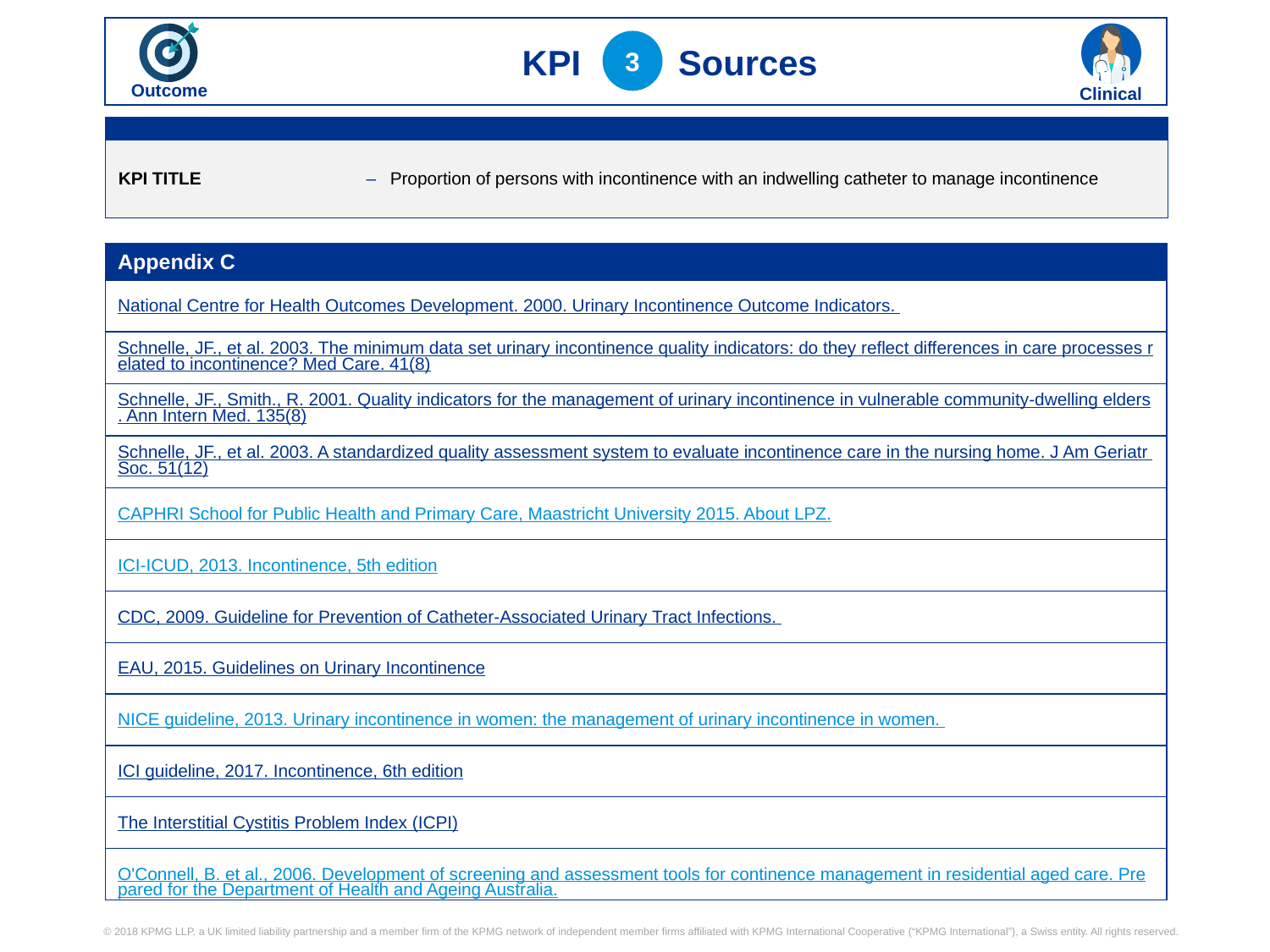

KPI Sources
3
Outcome
Clinical
| | |
| --- | --- |
| KPI TITLE | Proportion of persons with incontinence with an indwelling catheter to manage incontinence |
| Appendix C |
| --- |
| National Centre for Health Outcomes Development. 2000. Urinary Incontinence Outcome Indicators. |
| Schnelle, JF., et al. 2003. The minimum data set urinary incontinence quality indicators: do they reflect differences in care processes related to incontinence? Med Care. 41(8) |
| Schnelle, JF., Smith., R. 2001. Quality indicators for the management of urinary incontinence in vulnerable community-dwelling elders. Ann Intern Med. 135(8) |
| Schnelle, JF., et al. 2003. A standardized quality assessment system to evaluate incontinence care in the nursing home. J Am Geriatr Soc. 51(12) |
| CAPHRI School for Public Health and Primary Care, Maastricht University 2015. About LPZ. |
| ICI-ICUD, 2013. Incontinence, 5th edition |
| CDC, 2009. Guideline for Prevention of Catheter-Associated Urinary Tract Infections. |
| EAU, 2015. Guidelines on Urinary Incontinence |
| NICE guideline, 2013. Urinary incontinence in women: the management of urinary incontinence in women. |
| ICI guideline, 2017. Incontinence, 6th edition |
| The Interstitial Cystitis Problem Index (ICPI) |
| O'Connell, B. et al., 2006. Development of screening and assessment tools for continence management in residential aged care. Prepared for the Department of Health and Ageing Australia. |

## Slide 46
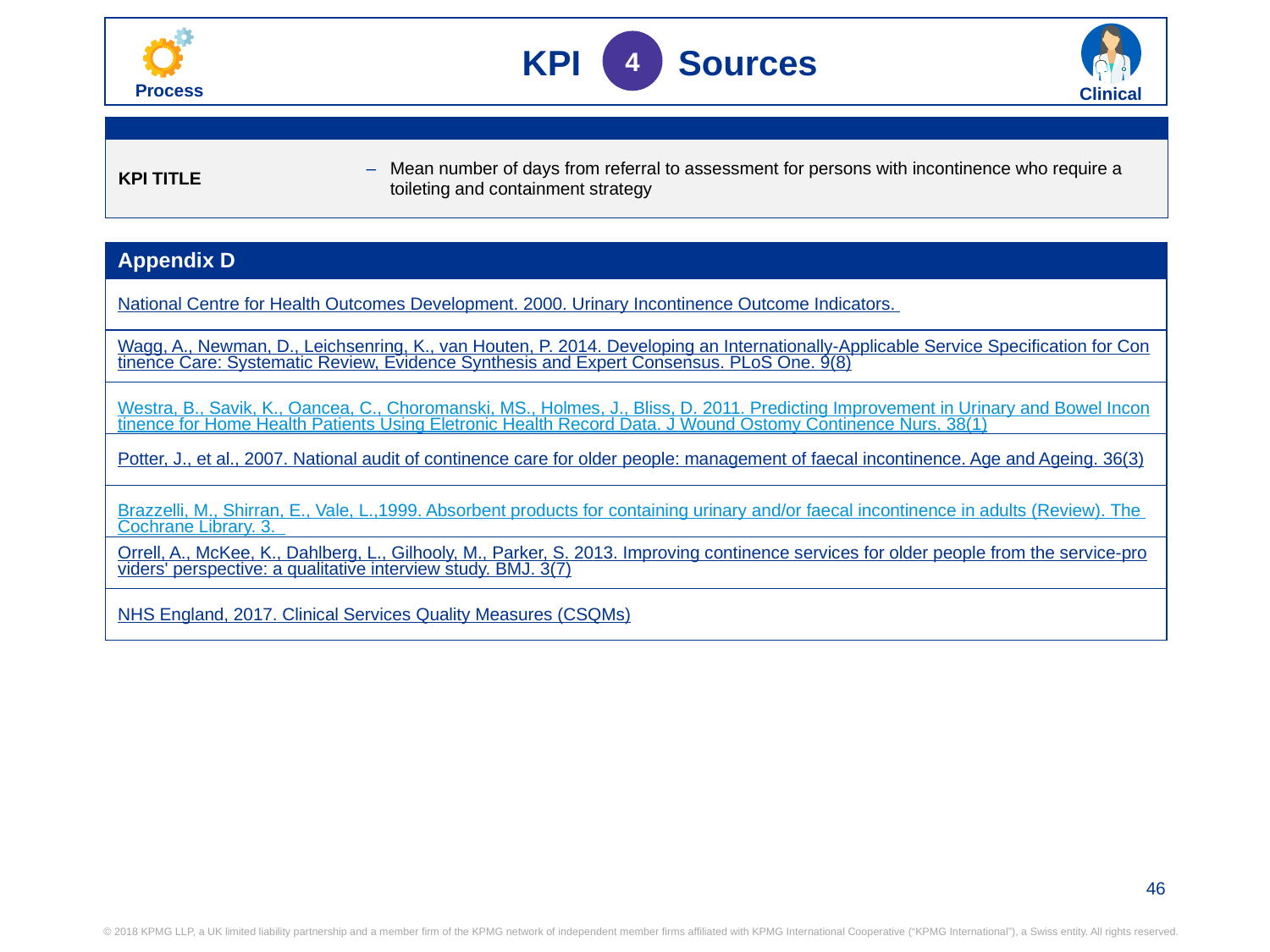

KPI Sources
4
Process
Clinical
| | |
| --- | --- |
| KPI TITLE | Mean number of days from referral to assessment for persons with incontinence who require a toileting and containment strategy |
| Appendix D |
| --- |
| National Centre for Health Outcomes Development. 2000. Urinary Incontinence Outcome Indicators. |
| Wagg, A., Newman, D., Leichsenring, K., van Houten, P. 2014. Developing an Internationally-Applicable Service Specification for Continence Care: Systematic Review, Evidence Synthesis and Expert Consensus. PLoS One. 9(8) |
| Westra, B., Savik, K., Oancea, C., Choromanski, MS., Holmes, J., Bliss, D. 2011. Predicting Improvement in Urinary and Bowel Incontinence for Home Health Patients Using Eletronic Health Record Data. J Wound Ostomy Continence Nurs. 38(1) |
| Potter, J., et al., 2007. National audit of continence care for older people: management of faecal incontinence. Age and Ageing. 36(3) |
| Brazzelli, M., Shirran, E., Vale, L.,1999. Absorbent products for containing urinary and/or faecal incontinence in adults (Review). The Cochrane Library. 3. |
| Orrell, A., McKee, K., Dahlberg, L., Gilhooly, M., Parker, S. 2013. Improving continence services for older people from the service-providers' perspective: a qualitative interview study. BMJ. 3(7) |
| NHS England, 2017. Clinical Services Quality Measures (CSQMs) |

## Slide 47
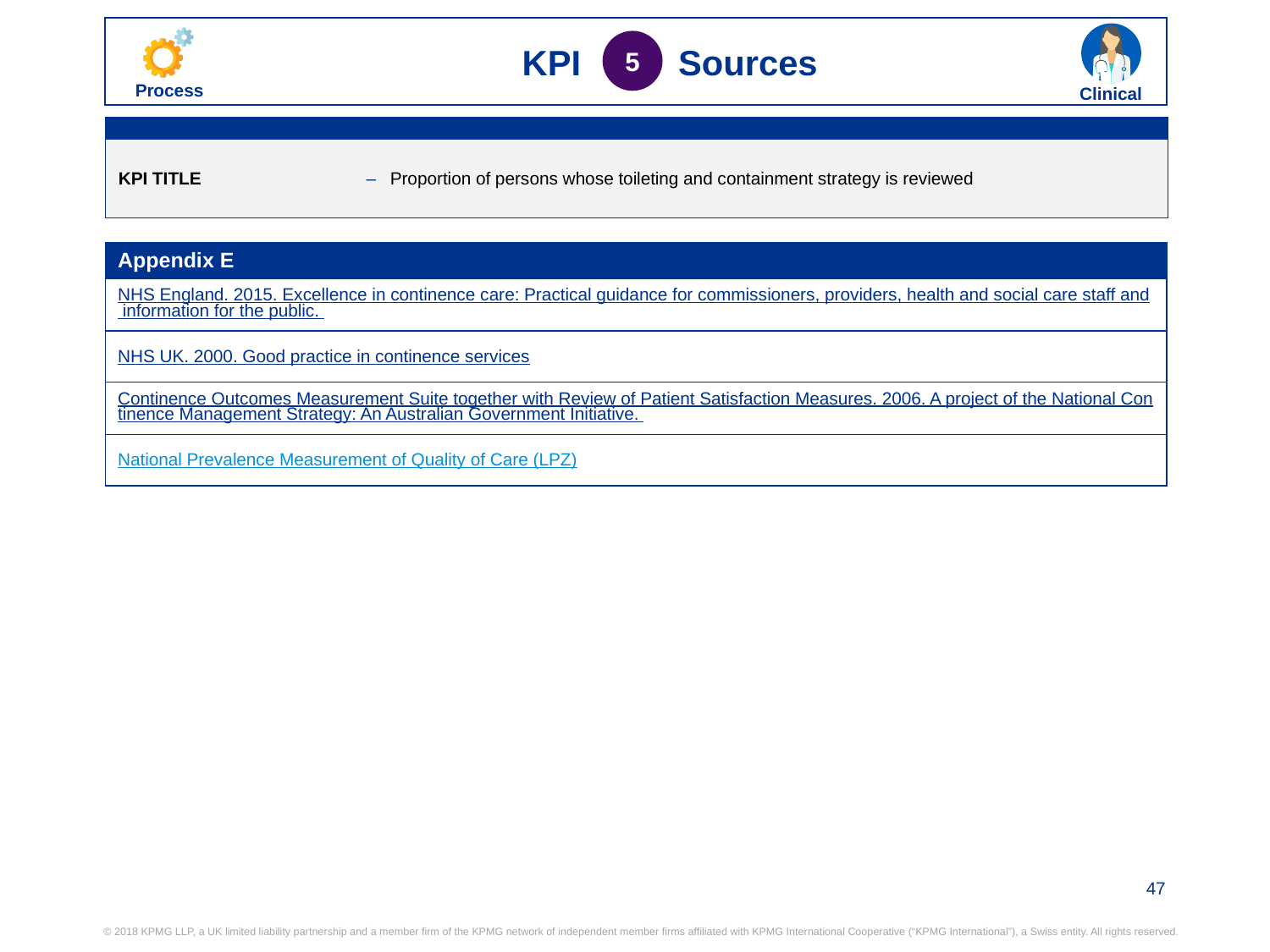

KPI Sources
5
Process
Clinical
| | |
| --- | --- |
| KPI TITLE | Proportion of persons whose toileting and containment strategy is reviewed |
| Appendix E |
| --- |
| NHS England. 2015. Excellence in continence care: Practical guidance for commissioners, providers, health and social care staff and information for the public. |
| NHS UK. 2000. Good practice in continence services |
| Continence Outcomes Measurement Suite together with Review of Patient Satisfaction Measures. 2006. A project of the National Continence Management Strategy: An Australian Government Initiative. |
| National Prevalence Measurement of Quality of Care (LPZ) |

## Slide 48
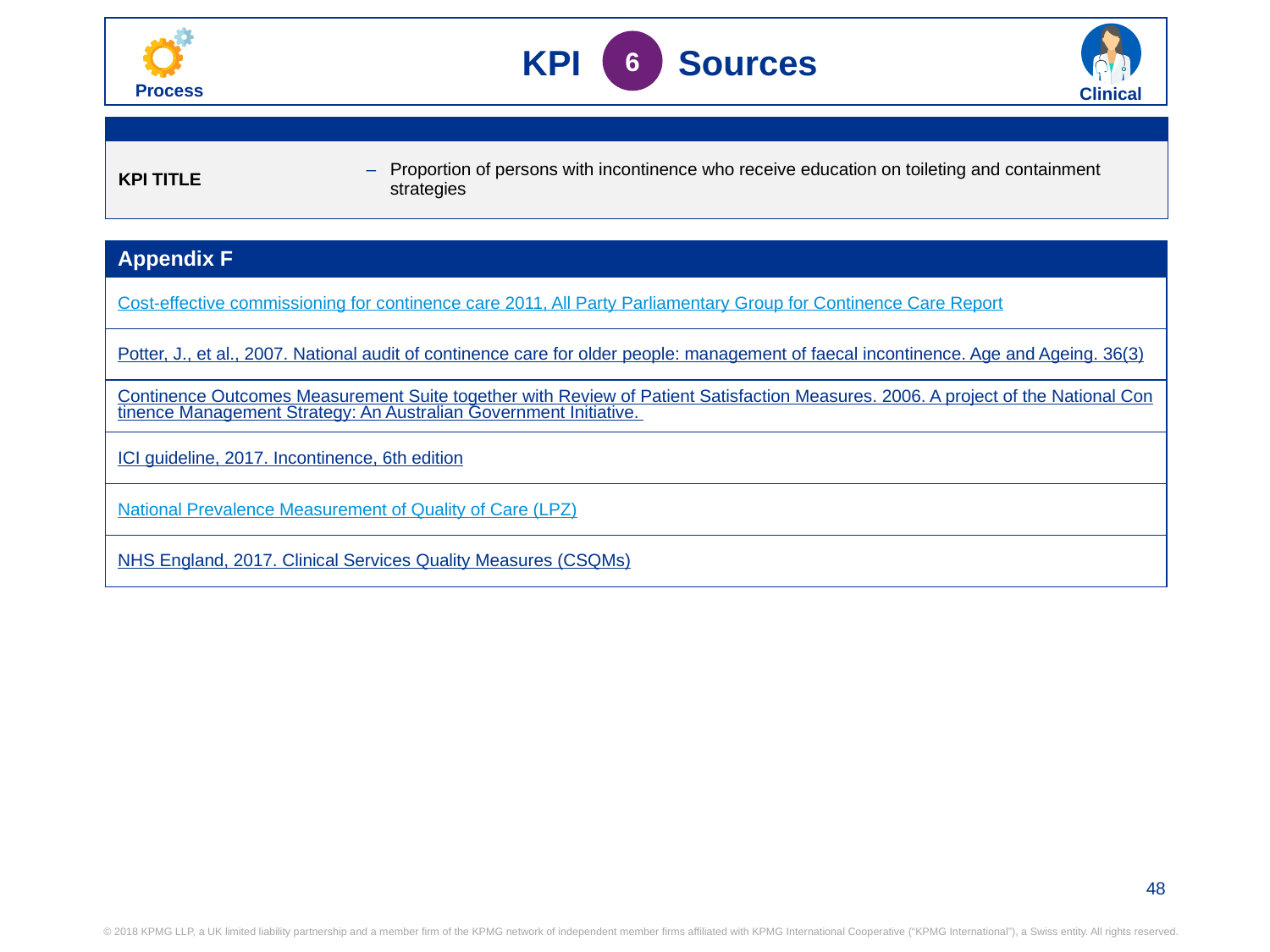

KPI Sources
6
Process
Clinical
| | |
| --- | --- |
| KPI TITLE | Proportion of persons with incontinence who receive education on toileting and containment strategies |
| Appendix F |
| --- |
| Cost-effective commissioning for continence care 2011, All Party Parliamentary Group for Continence Care Report |
| Potter, J., et al., 2007. National audit of continence care for older people: management of faecal incontinence. Age and Ageing. 36(3) |
| Continence Outcomes Measurement Suite together with Review of Patient Satisfaction Measures. 2006. A project of the National Continence Management Strategy: An Australian Government Initiative. |
| ICI guideline, 2017. Incontinence, 6th edition |
| National Prevalence Measurement of Quality of Care (LPZ) |
| NHS England, 2017. Clinical Services Quality Measures (CSQMs) |

## Slide 49
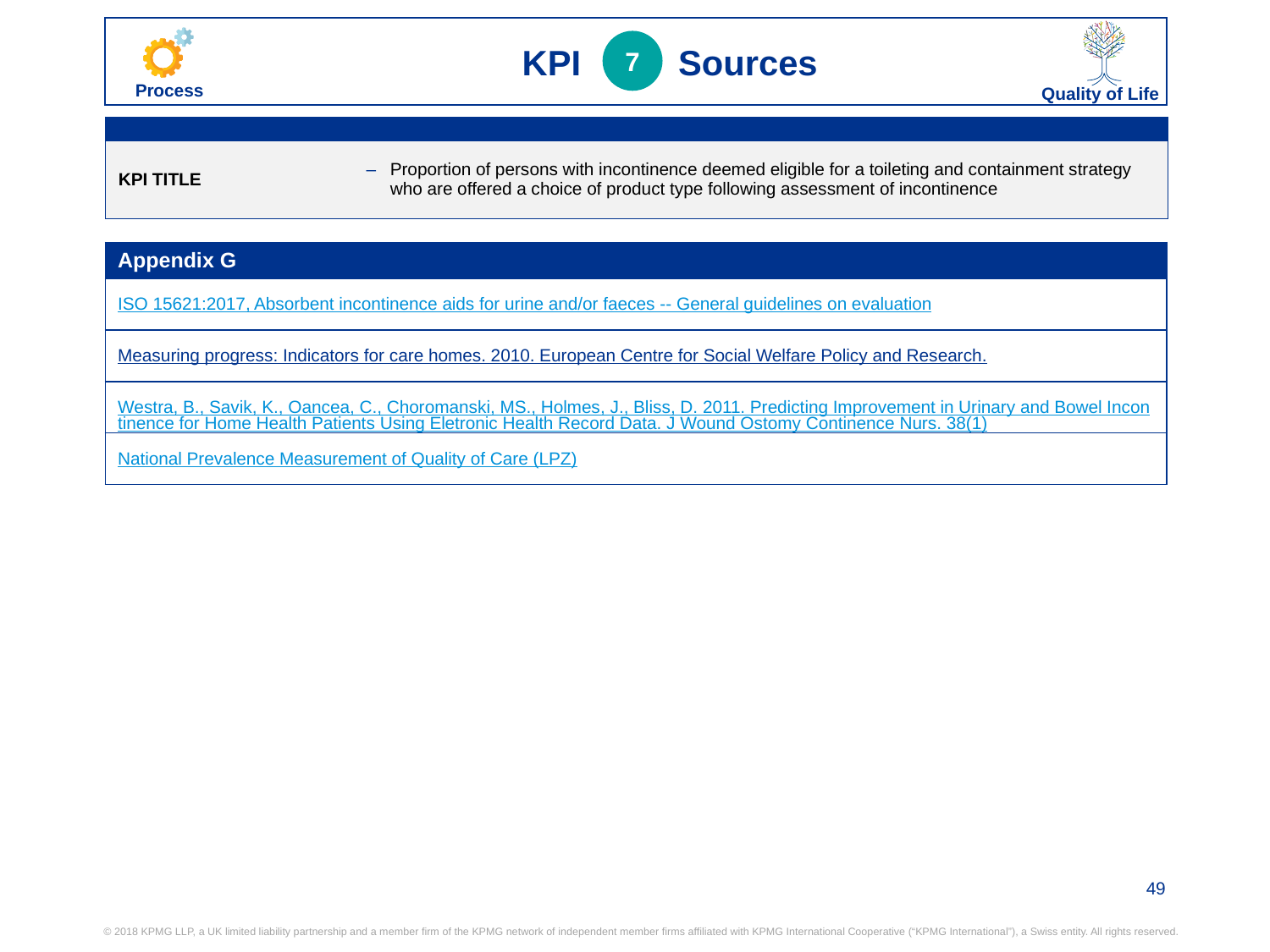

KPI Sources
7
Process
Quality of Life
| | |
| --- | --- |
| KPI TITLE | Proportion of persons with incontinence deemed eligible for a toileting and containment strategy who are offered a choice of product type following assessment of incontinence |
| Appendix G |
| --- |
| ISO 15621:2017, Absorbent incontinence aids for urine and/or faeces -- General guidelines on evaluation |
| Measuring progress: Indicators for care homes. 2010. European Centre for Social Welfare Policy and Research. |
| Westra, B., Savik, K., Oancea, C., Choromanski, MS., Holmes, J., Bliss, D. 2011. Predicting Improvement in Urinary and Bowel Incontinence for Home Health Patients Using Eletronic Health Record Data. J Wound Ostomy Continence Nurs. 38(1) |
| National Prevalence Measurement of Quality of Care (LPZ) |

## Slide 50
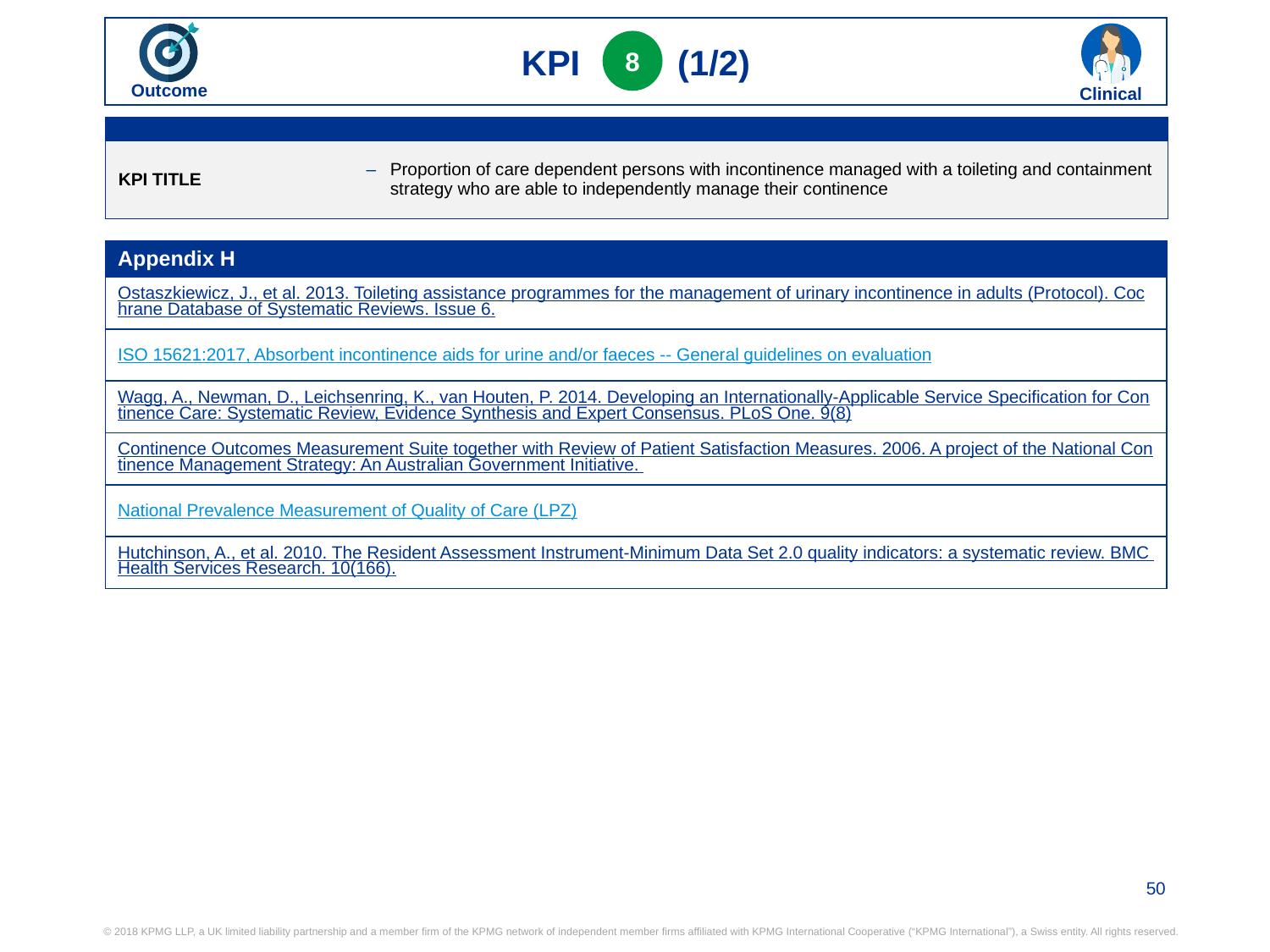

KPI (1/2)
8
Outcome
Clinical
| | |
| --- | --- |
| KPI TITLE | Proportion of care dependent persons with incontinence managed with a toileting and containment strategy who are able to independently manage their continence |
| Appendix H |
| --- |
| Ostaszkiewicz, J., et al. 2013. Toileting assistance programmes for the management of urinary incontinence in adults (Protocol). Cochrane Database of Systematic Reviews. Issue 6. |
| ISO 15621:2017, Absorbent incontinence aids for urine and/or faeces -- General guidelines on evaluation |
| Wagg, A., Newman, D., Leichsenring, K., van Houten, P. 2014. Developing an Internationally-Applicable Service Specification for Continence Care: Systematic Review, Evidence Synthesis and Expert Consensus. PLoS One. 9(8) |
| Continence Outcomes Measurement Suite together with Review of Patient Satisfaction Measures. 2006. A project of the National Continence Management Strategy: An Australian Government Initiative. |
| National Prevalence Measurement of Quality of Care (LPZ) |
| Hutchinson, A., et al. 2010. The Resident Assessment Instrument-Minimum Data Set 2.0 quality indicators: a systematic review. BMC Health Services Research. 10(166). |

## Slide 51
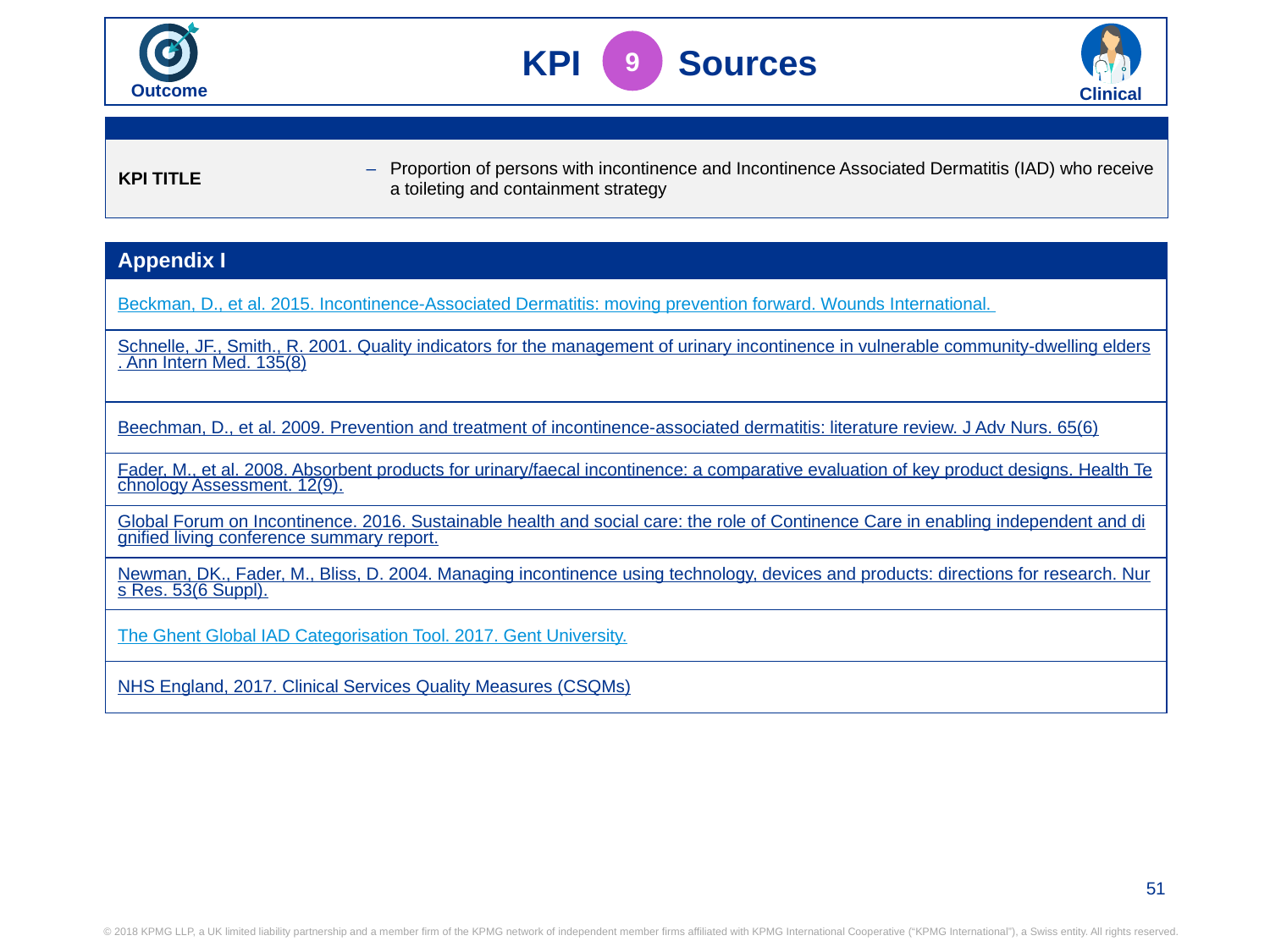

KPI Sources
9
Outcome
Clinical
| | |
| --- | --- |
| KPI TITLE | Proportion of persons with incontinence and Incontinence Associated Dermatitis (IAD) who receive a toileting and containment strategy |
| Appendix I |
| --- |
| Beckman, D., et al. 2015. Incontinence-Associated Dermatitis: moving prevention forward. Wounds International. |
| Schnelle, JF., Smith., R. 2001. Quality indicators for the management of urinary incontinence in vulnerable community-dwelling elders. Ann Intern Med. 135(8) |
| Beechman, D., et al. 2009. Prevention and treatment of incontinence-associated dermatitis: literature review. J Adv Nurs. 65(6) |
| Fader, M., et al. 2008. Absorbent products for urinary/faecal incontinence: a comparative evaluation of key product designs. Health Technology Assessment. 12(9). |
| Global Forum on Incontinence. 2016. Sustainable health and social care: the role of Continence Care in enabling independent and dignified living conference summary report. |
| Newman, DK., Fader, M., Bliss, D. 2004. Managing incontinence using technology, devices and products: directions for research. Nurs Res. 53(6 Suppl). |
| The Ghent Global IAD Categorisation Tool. 2017. Gent University. |
| NHS England, 2017. Clinical Services Quality Measures (CSQMs) |

## Slide 52
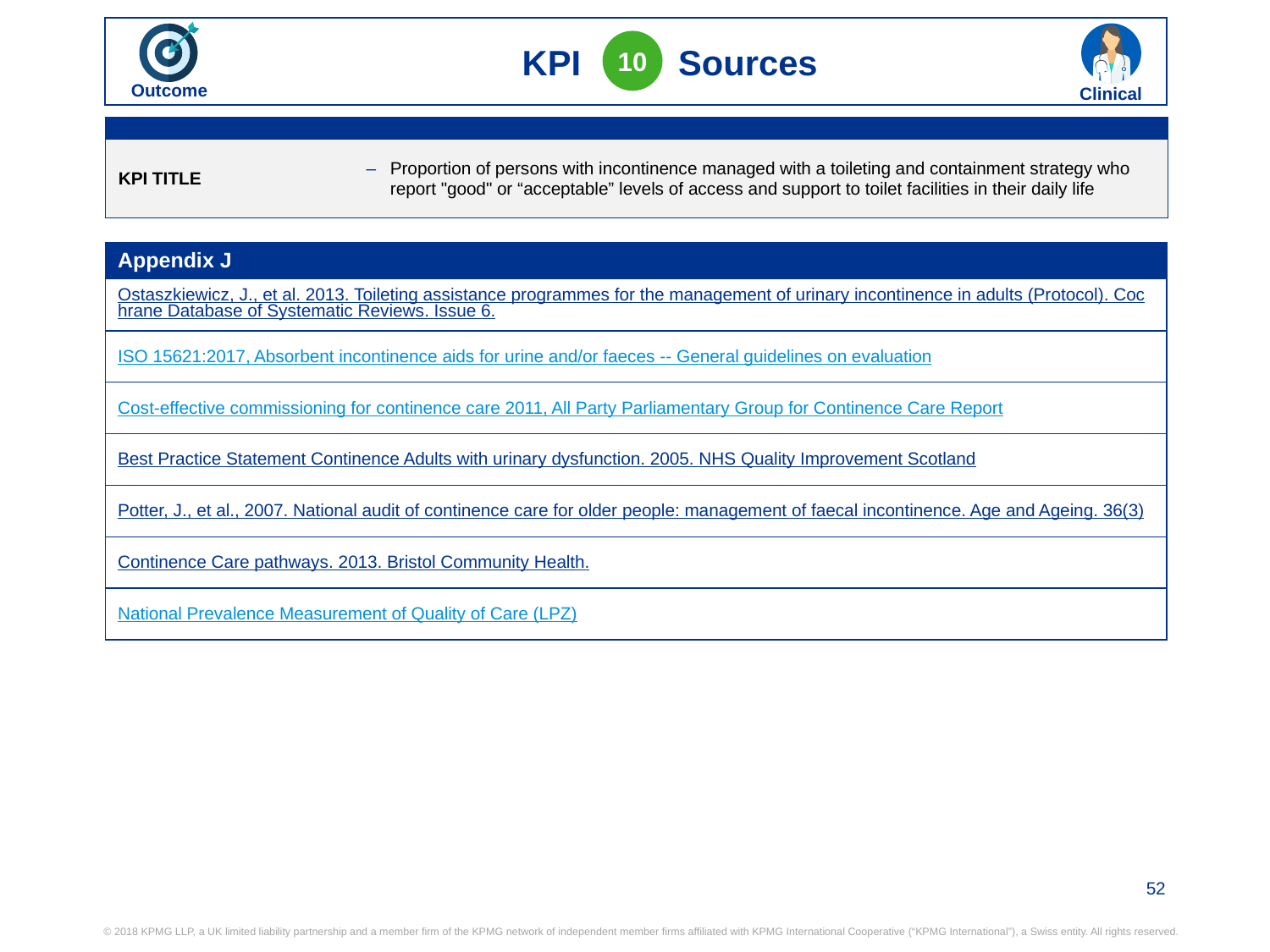

KPI Sources
10
Outcome
Clinical
| | |
| --- | --- |
| KPI TITLE | Proportion of persons with incontinence managed with a toileting and containment strategy who report "good" or “acceptable” levels of access and support to toilet facilities in their daily life |
| Appendix J |
| --- |
| Ostaszkiewicz, J., et al. 2013. Toileting assistance programmes for the management of urinary incontinence in adults (Protocol). Cochrane Database of Systematic Reviews. Issue 6. |
| ISO 15621:2017, Absorbent incontinence aids for urine and/or faeces -- General guidelines on evaluation |
| Cost-effective commissioning for continence care 2011, All Party Parliamentary Group for Continence Care Report |
| Best Practice Statement Continence Adults with urinary dysfunction. 2005. NHS Quality Improvement Scotland |
| Potter, J., et al., 2007. National audit of continence care for older people: management of faecal incontinence. Age and Ageing. 36(3) |
| Continence Care pathways. 2013. Bristol Community Health. |
| National Prevalence Measurement of Quality of Care (LPZ) |

## Slide 53
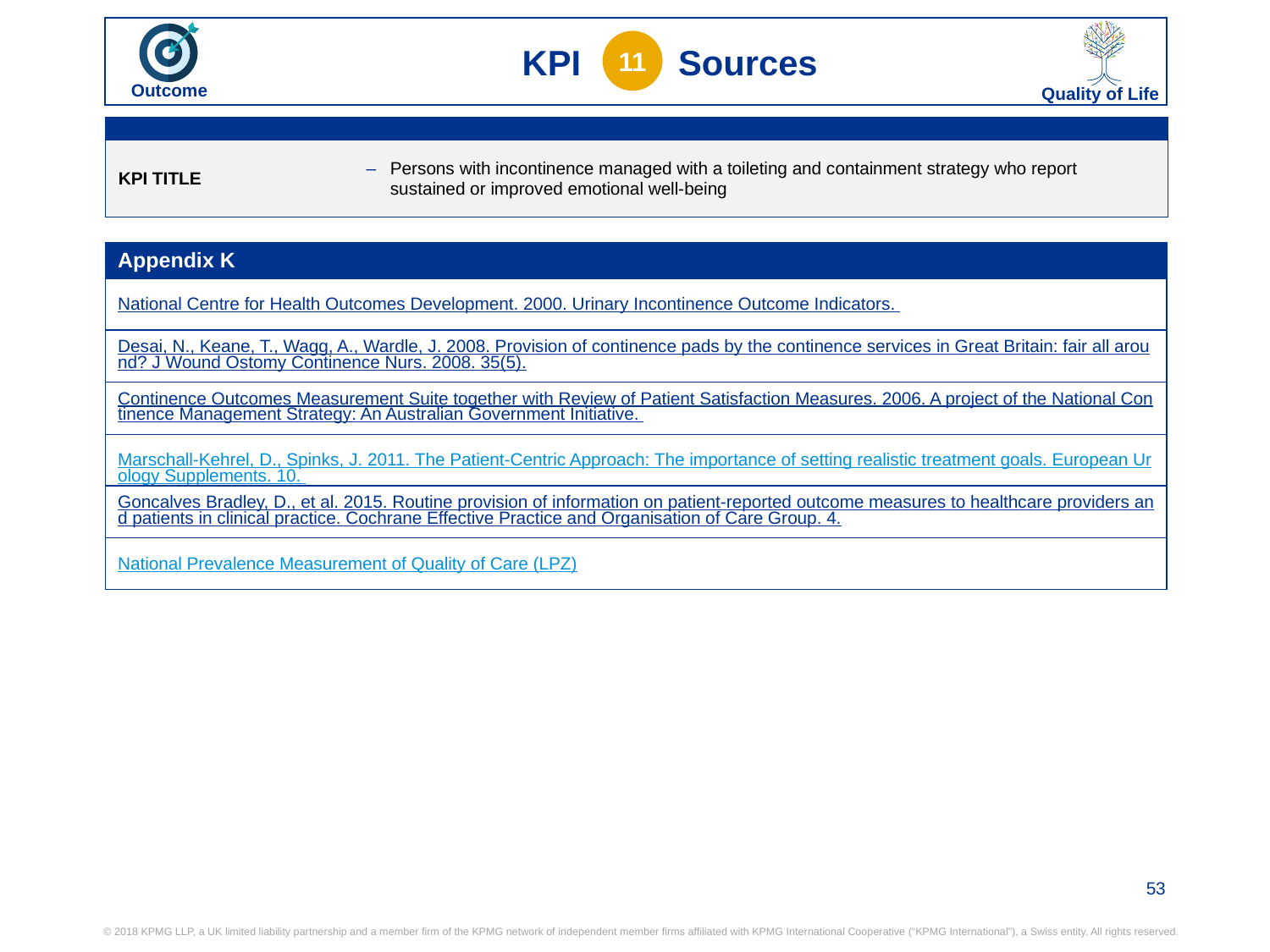

KPI Sources
11
Outcome
Quality of Life
| | |
| --- | --- |
| KPI TITLE | Persons with incontinence managed with a toileting and containment strategy who report sustained or improved emotional well-being |
| Appendix K |
| --- |
| National Centre for Health Outcomes Development. 2000. Urinary Incontinence Outcome Indicators. |
| Desai, N., Keane, T., Wagg, A., Wardle, J. 2008. Provision of continence pads by the continence services in Great Britain: fair all around? J Wound Ostomy Continence Nurs. 2008. 35(5). |
| Continence Outcomes Measurement Suite together with Review of Patient Satisfaction Measures. 2006. A project of the National Continence Management Strategy: An Australian Government Initiative. |
| Marschall-Kehrel, D., Spinks, J. 2011. The Patient-Centric Approach: The importance of setting realistic treatment goals. European Urology Supplements. 10. |
| Goncalves Bradley, D., et al. 2015. Routine provision of information on patient-reported outcome measures to healthcare providers and patients in clinical practice. Cochrane Effective Practice and Organisation of Care Group. 4. |
| National Prevalence Measurement of Quality of Care (LPZ) |

## Slide 54
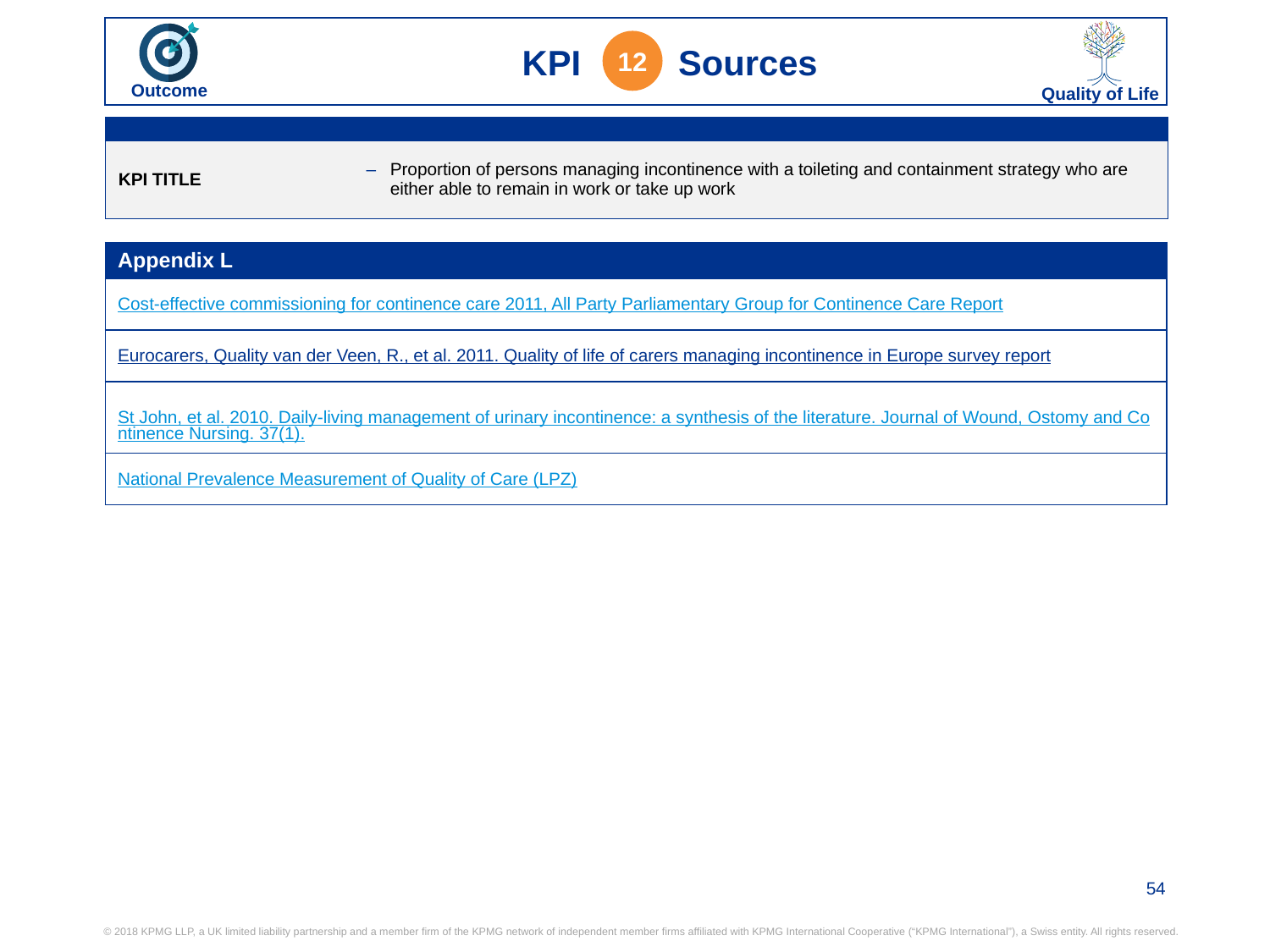

KPI Sources
12
Outcome
Quality of Life
| | |
| --- | --- |
| KPI TITLE | Proportion of persons managing incontinence with a toileting and containment strategy who are either able to remain in work or take up work |
| Appendix L |
| --- |
| Cost-effective commissioning for continence care 2011, All Party Parliamentary Group for Continence Care Report |
| Eurocarers, Quality van der Veen, R., et al. 2011. Quality of life of carers managing incontinence in Europe survey report |
| St John, et al. 2010. Daily-living management of urinary incontinence: a synthesis of the literature. Journal of Wound, Ostomy and Continence Nursing. 37(1). |
| National Prevalence Measurement of Quality of Care (LPZ) |

## Slide 55
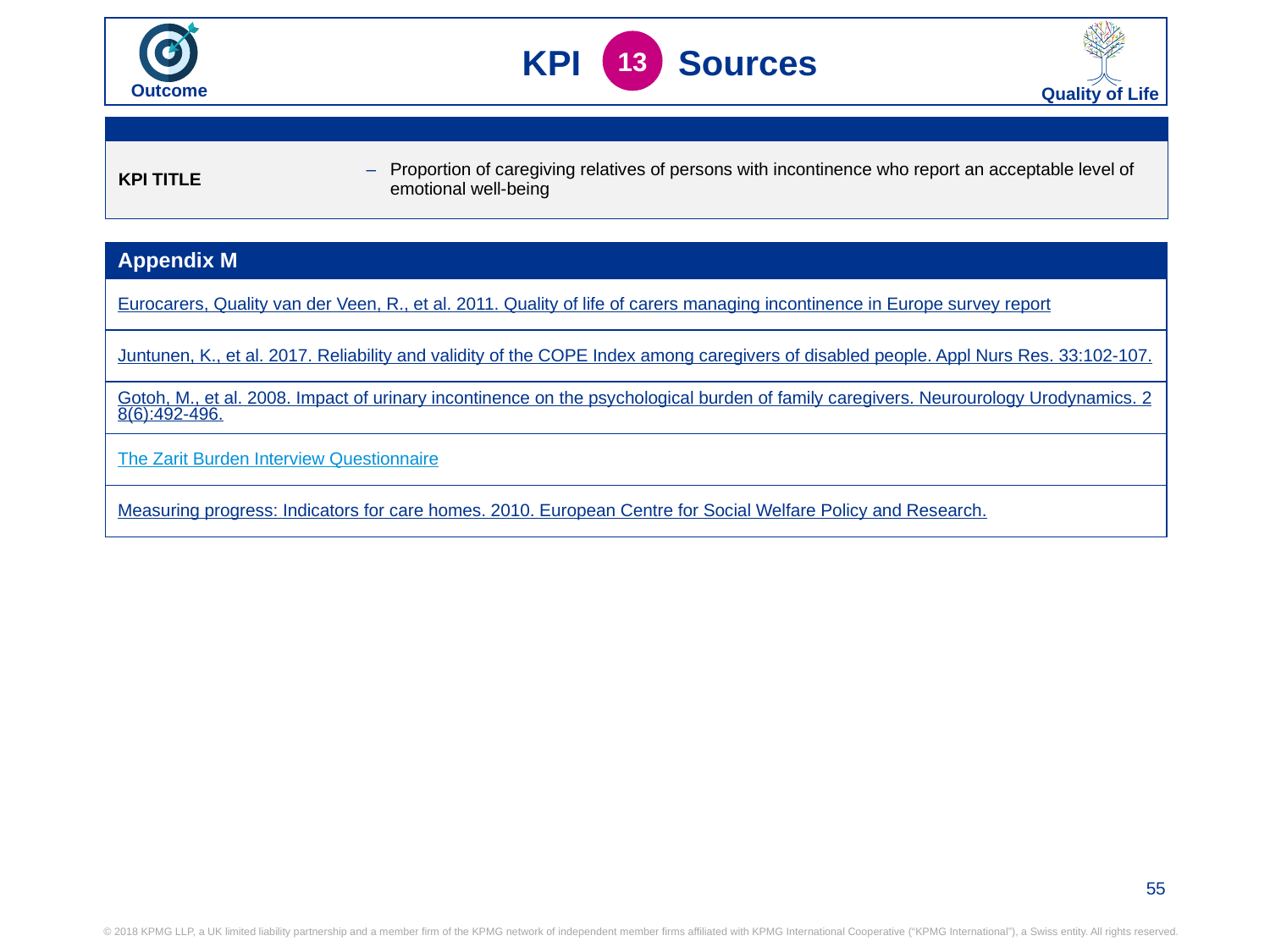

KPI Sources
13
Outcome
Quality of Life
| | |
| --- | --- |
| KPI TITLE | Proportion of caregiving relatives of persons with incontinence who report an acceptable level of emotional well-being |
| Appendix M |
| --- |
| Eurocarers, Quality van der Veen, R., et al. 2011. Quality of life of carers managing incontinence in Europe survey report |
| Juntunen, K., et al. 2017. Reliability and validity of the COPE Index among caregivers of disabled people. Appl Nurs Res. 33:102-107. |
| Gotoh, M., et al. 2008. Impact of urinary incontinence on the psychological burden of family caregivers. Neurourology Urodynamics. 28(6):492-496. |
| The Zarit Burden Interview Questionnaire |
| Measuring progress: Indicators for care homes. 2010. European Centre for Social Welfare Policy and Research. |

## Slide 56
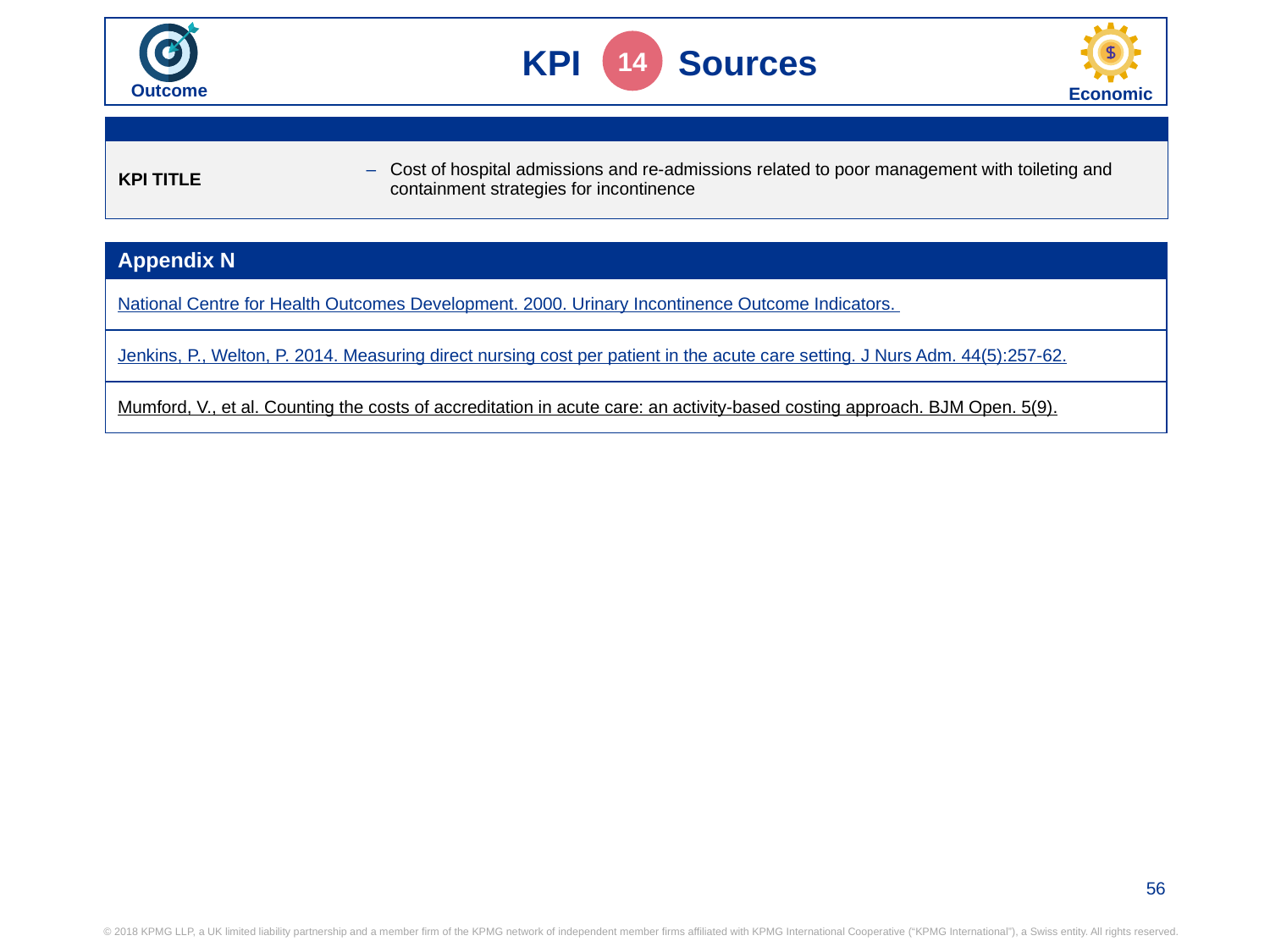

KPI Sources
14
Outcome
Economic
| | |
| --- | --- |
| KPI TITLE | Cost of hospital admissions and re-admissions related to poor management with toileting and containment strategies for incontinence |
| Appendix N |
| --- |
| National Centre for Health Outcomes Development. 2000. Urinary Incontinence Outcome Indicators. |
| Jenkins, P., Welton, P. 2014. Measuring direct nursing cost per patient in the acute care setting. J Nurs Adm. 44(5):257-62. |
| Mumford, V., et al. Counting the costs of accreditation in acute care: an activity-based costing approach. BJM Open. 5(9). |

## Slide 57
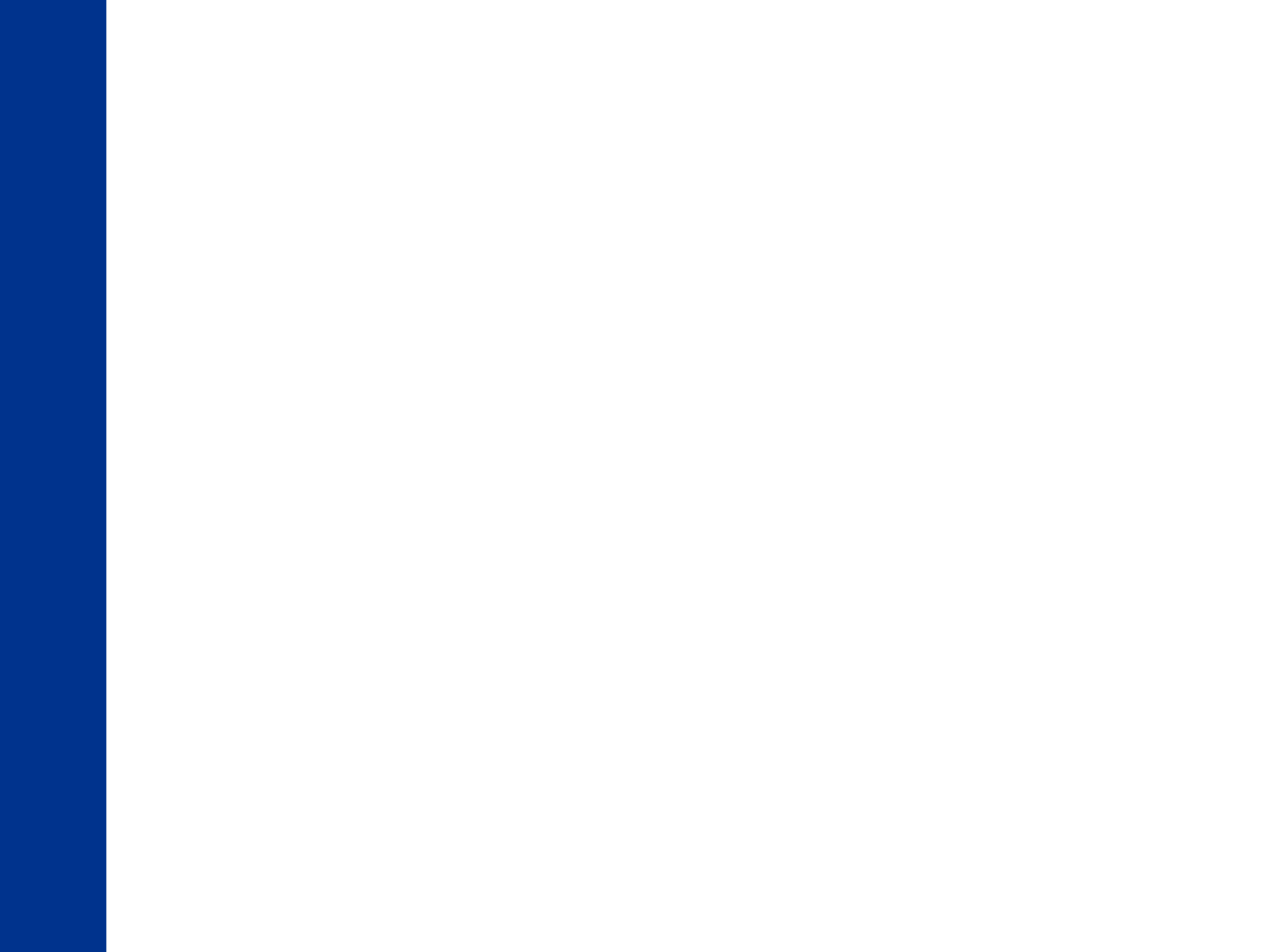

Supplement: Supplementary file 4 — Broad stakeholder engagement exercise key themes. C. Refined short list of 35 KPI titles. D. Detailed core KPI (PPTX 5.54 MB) [file 192_2018_3768_MOESM4_ESM.pptx]
